# Impact of preoperative weight-loss interventions on outcomes after elective non-bariatric surgery: meta-analysis

**DOI:** 10.1093/bjsopen/zrag001

**Published:** 2026-02-25

**Authors:** Danni Wang, Simon J A Buczacki, Qiufeng Gu, Zhengmei Liao, Yanli Jiang, Sam West, Dimitrios A Koutoukidis

**Affiliations:** Department of Health Promotion and Behaviour Sciences, Anhui Medical University, Hefei, China; Nuffield Department of Primary Care Health Sciences, University of Oxford, Oxford, UK; Nuffield Department of Surgical Sciences, University of Oxford, Oxford, UK; Department of Health Promotion and Behaviour Sciences, Anhui Medical University, Hefei, China; Department of Health Promotion and Behaviour Sciences, Anhui Medical University, Hefei, China; Department of Health Promotion and Behaviour Sciences, Anhui Medical University, Hefei, China; Nuffield Department of Primary Care Health Sciences, University of Oxford, Oxford, UK; Nuffield Department of Primary Care Health Sciences, University of Oxford, Oxford, UK

## Abstract

**Background:**

Obesity disproportionately affects patients awaiting elective non-bariatric surgery and complicates perioperative management. This systematic review aimed to assess the impact of weight-loss interventions on intraoperative and postoperative outcomes.

**Methods:**

MEDLINE, Embase, CINAHL, and Web of Science databases were searched from inception to October 2025 for trials on weight-loss interventions. Two reviewers independently screened the studies, extracted relevant data, and assessed risk of bias. Pooled mean differences (MDs), standardized mean differences (SMDs), and odds ratios (ORs) were obtained from random-effects meta-analyses.

**Results:**

Thirty-five studies with 9378 participants (mean(standard deviation) age 58(8) years; body mass index 35.6(6.4) kg/m^2^; 61% women) were included. The median duration of intervention was 8 (interquartile range 4–14) weeks. Preoperative weight-loss interventions were significantly associated with a reduction in overall postoperative complications (odd ratio (OR) 0.63, 95% confidence interval (c.i.) 0.43 to 0.93; *I*² = 32%) and in complications requiring medical intervention graded as Clavien–Dindo ≥ II (OR 0.66, 0.51 to 0.86; *I*² = 0%). Additionally, they were linked to a decreased risk of postoperative non-infectious wound-related complications (OR 0.38, 0.15 to 0.97; *I*^2^ = 0%), and with reduced intraoperative blood loss in gastrectomy (SMD −0.98, 95% c.i. −1.47 to −0.48; *I*^2^ = 0%) and hepatectomy (SMD −0.41, −0.82 to 0.00; *I*^2^ = 0%). Reductions in blood transfusion (OR 0.49, 0.31 to 0.79; *I*² = 0%), hospital readmission rates (OR 0.57, 0.47 to 0.70; *I*² = 0%), and length of hospital stay (SMD −0.08, −0.13 to −0.04; *I*² = 0%) were also noted. No association was observed for surgical site infection, venous thromboembolism, or return to the emergency department. Compared with standard care or no intervention, weight-loss interventions led to greater weight loss (MD −3.92 (95% c.i. −5.44 to −2.39) kg; *I^2^* = 91%), and fat mass loss (MD −4.78 (−6.49 to −3.06) kg; *I^2^* = 0%) but no change in lean mass (SMD −0.25, −0.51 to 0.01; *I^2^* = 0%). In a sensitivity analysis of studies at low risk of bias, the estimates and precision of most outcomes did not change materially.

**Conclusion:**

Despite heterogeneity in study design and surgical populations, the evidence consistently demonstrated that weight-loss interventions are feasible, safe, and can reduce postoperative complications across various surgical specialties alongside improving many outcomes.

## Teaser

A systematic review and meta-analysis was undertaken to evaluate the effects of weight-loss interventions in adults with overweight or obesity awaiting elective non-bariatric surgery. The findings suggest that such interventions are feasible and safe, and can reduce postoperative complications across various surgical specialties alongside improving many postoperative outcomes.

## Introduction

The prevalence of obesity has risen substantially over the past three decades^1^. In the USA and the UK, the prevalence of obesity in the general population is 40%^2^ and 29%^3^, respectively, and projected to rise even further by 2050^4^. However, the prevalence within populations awaiting surgery is higher, with 45, 67, and 69% of patients awaiting non-bariatric elective surgery, total joint arthroplasty, and ventral hernia repair, respectively, being affected by obesity^5–7^.

Obesity is an established risk factor for perioperative complications, including prolonged operating time, excessive intraoperative blood loss, increased impaired wound healing, venous thromboembolism, and extended hospital stay^5,7–10^. The underlying mechanisms likely involve excess adipose tissue increasing technical challenges for surgical access and visualization^11^. Additionally chronic low-grade inflammation disrupts immune responses and collagen synthesis that can impair wound healing^12^. Raised intra-abdominal pressure, systemic inflammation, adipokine imbalance promoting tissue factor expression, and a procoagulant state can increase the risk of venous thromboembolism^13^. Given these risks, preoperative weight-loss interventions have been explored across various elective surgical specialties as a potential strategy to improve perioperative outcomes^11,14,15^.

Although weight-loss interventions, including dietary, behavioural, and pharmacological interventions, are widely implemented before bariatric surgery, their effectiveness in non-bariatric elective surgery remains uncertain^16^. Existing systematic reviews^11,16–19^ have focused primarily on dietary interventions excluding pharmacotherapy or specific surgical settings, such as abdominal surgery^11^, gastrointestinal surgery^19^, ventral hernia repair^20^, and joint arthroplasty​^15^. Although a recent comprehensive review^21^ included both bariatric and non-bariatric procedures, it did not focus specifically on the non-bariatric elective setting. Moreover, several new studies^22–26^ have emerged since the publication of earlier reviews, warranting an updated synthesis. Furthermore, some reviews have incorporated studies encompassing a broad body mass index (BMI) spectrum, including individuals with normal BMI^16^, and patients in whom preoperative weight loss was not explicitly categorized as intentional^15^, potentially confounding its association with surgical outcomes. Importantly, existing reviews have rarely evaluated changes in body composition (such as fat mass or lean mass), and none to date have undertaken meta-analyses of these outcomes.

The aim of the study was to systematically review and meta-analyse the impact of preoperative weight-loss interventions on perioperative outcomes in non-bariatric elective surgery.

## Methods

The protocol was registered in advance in PROSPERO (CRD42024610636) and the review was conducted following the PRISMA guidelines for systematic reviews and meta-analyses^27^.

### Eligibility criteria

Studies evaluating weight-loss interventions in adult patients who were overweight and/or obese (as defined in the primary studies) awaiting non-bariatric elective surgery were considered eligible. Eligible study designs included randomized clinical trials (RCTs), non-randomized comparative studies, and single-arm trials. All types of elective surgery were eligible, including cancer-related procedures, except for bariatric surgery, body-contouring procedures, plastic surgery, and studies involving artificial reproductive technology. Studies that did not specifically focus on patients who were overweight or obese were excluded

Eligible interventions included behavioural weight-loss programmes (BWLPs) and pharmacotherapy approved for weight management. Dietary interventions that did not explicitly state weight loss as their primary goal and exercise-only interventions were excluded. In comparative studies, eligible comparator groups received either usual care without a specific preoperative weight-loss intervention or no intervention.

To be eligible for inclusion, trials were required to report at least one intraoperative or postoperative outcome. Intraoperative outcomes included but were not limited to operating time and blood loss. Postoperative outcomes encompassed: mortality; morbidity (for example classified using the Clavien–Dindo system); wound-related complications as both infectious (surgical site infection, SSI) and non-infectious (such as seroma, haematoma, non-healing wound) events; remote infections (for example urinary tract and chest infections); venous thromboembolism; hospital readmission; and length hospital of stay (LOS). Where reported, data on body composition outcomes were extracted (for example fat mass and lean mass).

### Literature search

Five databases from inception to 30 October 2025 were searched: MEDLINE (OvidSP) (1946 onwards), Embase (OvidSP) (1974 onwards), CINAHL (EBSCOhost) (1982 onwards), Science Citation Index, and Conference Proceedings Citation Index—Science (Web of Science Core Collection) (1900 onwards). The search strategy targeted titles, abstracts, author keywords, and subject headings for the authors’ key concepts of preoperative weight loss in non-bariatric surgery. Methodological search filters were applied to restrict to trials and observational studies^28^. The search was developed in MEDLINE and adapted with the aid of Polyglot^29^ for the other databases. No date or language limits were applied. Search results were exported to EndNote® (Clarivate Analytics, Philadelphia, PA, USA), where animal studies were excluded, and the remaining references were imported into Covidence, where duplicate records were removed. The search strategy (*supplementary methods*) was developed by an experienced librarian. Additionally, references were screened manually from systematic reviews on weight-loss interventions before elective non-bariatric surgery.

Among the five reviewers (D.W., Q.G., Z.L., Y.J., and D.A.K.), D.W. was paired with another reviewer for each study to independently screen the title, abstract, and full text using Covidence^30^, and to extract data into a pilot-tested and bespoke extraction file in Microsoft^®^ Excel (Microsoft, Redmond, WA, USA). Any discrepancies in screening and data extraction were resolved by discussion, or by referral to a third reviewer (D.A.K.). Study authors were contacted for further data and/or clariﬁcations, if needed.

### Risk of bias (quality) assessment

D.W. and another reviewer independently assessed risk of bias. The revised Cochrane risk-of-bias tool for randomized trials (RoB 2)^31^ was applied to RCTs, whereas Risk Of Bias In Non-randomized studies—of interventions (ROBINS-I)^32^ was used for non-randomized studies, including single-arm trials. To ensure consistency in risk-of-bias assessment across randomized and non-randomized studies, the RoB 2 and ROBINS-I categories were harmonized into three levels: low, moderate, and high risk. Studies rated as ‘low risk’ by both tools were classified as low risk. ‘Some concerns’ in RoB 2 and ‘moderate risk’ in ROBINS-I were grouped as moderate risk. ‘High risk’ in RoB 2 and ‘serious’ or ‘critical risk’ in ROBINS-I were classified as high risk (*supplementary methods*). A high risk of attrition bias was defined as a difference in attrition rates between trial arms of > 20% in trials with multiple groups. Publication bias was assessed by visual inspection of funnel plots. The certainty of evidence was assessed qualitatively based on risk-of-bias, result consistency (RoB 2, ROBINS-I), and study heterogeneity.

### Data analysis

Weight change is reported from baseline (before intervention) to elective surgery. Surgical outcomes are expressed as means for continuous variables and as event rates for categorical outcomes. Outcomes from the intervention group in comparative studies were also included in the single-arm meta-analysis alongside other non-comparative studies.

Meta-analyses were undertaken for all outcomes in both comparative and single-arm studies, using random-effects models defined *a priori*, given the heterogeneity in interventions and outcome assessments. Continuous outcomes are summarized as mean differences (MDs) when measured using consistent and interpretable units (for example body weight, fat mass). Standardized mean differences (SMDs) and standardized means were used when outcomes were reported on different scales (for example lean mass), or when variation in study populations, surgical procedures, and reporting precision limited direct comparability despite use of consistent units (for example blood loss, operating time, LOS). In single-arm studies, weight change is reported as a difference in mean, continuous surgical outcomes as standardized means, and event-based surgical outcomes as proportions, all with 95% confidence intervals. Statistical heterogeneity was assessed with the *I^2^* statistic. The strength of evidence was evaluated based on the precision of confidence intervals, indication of clinically meaningful improvements, and the degree of heterogeneity.

If studies reported only BMI without baseline weight, the mean baseline weight was estimated using the mean height by sex for the respective country^33^. If continuous surgical outcomes were not reported as mean(standard deviation, s.d.), these were estimated using methods outlined in the Cochrane Handbook^34^ and adopting the recommended approach^35,36^. If data were presented in figures and additional data were not available from the authors, these were extracted using WebPlotDigitizer^37^.

Three prespecified additional analyses were carried out: a sensitivity analysis to test whether effect sizes changed after exclusion of studies with a high or unclear risk of bias; a meta-regression analysis to examine the relationship between duration of intervention and weight change while adjusting for the different types of intervention, including total diet replacement (TDR) or very low-calorie diet (VLCD), partial diet replacement (PDR) or low-calorie diet (LCD), and dietary advice alone; and a subgroup analysis based on the types of elective procedure reported in the original studies (for example hernia repair, cholecystectomy, arthroplasty, hepatectomy, gastrectomy, mixed surgical approach). *Post hoc* subgroup analyses was performed based on mean age (≤ 55, 56–65, ≥ 65 years), sex (> 75, 25–75, ≤ 25% women), mean baseline BMI (overweight, obesity class Ⅰ, obesity class Ⅱ, obesity class III), duration of intervention (short-term: < 4 weeks; medium-term: 4–14 weeks; long-term: > 14 weeks), and disease type (cancer *versus* non-cancer surgery).

## Results

### Study selection and characteristics

**Fig. 1:**
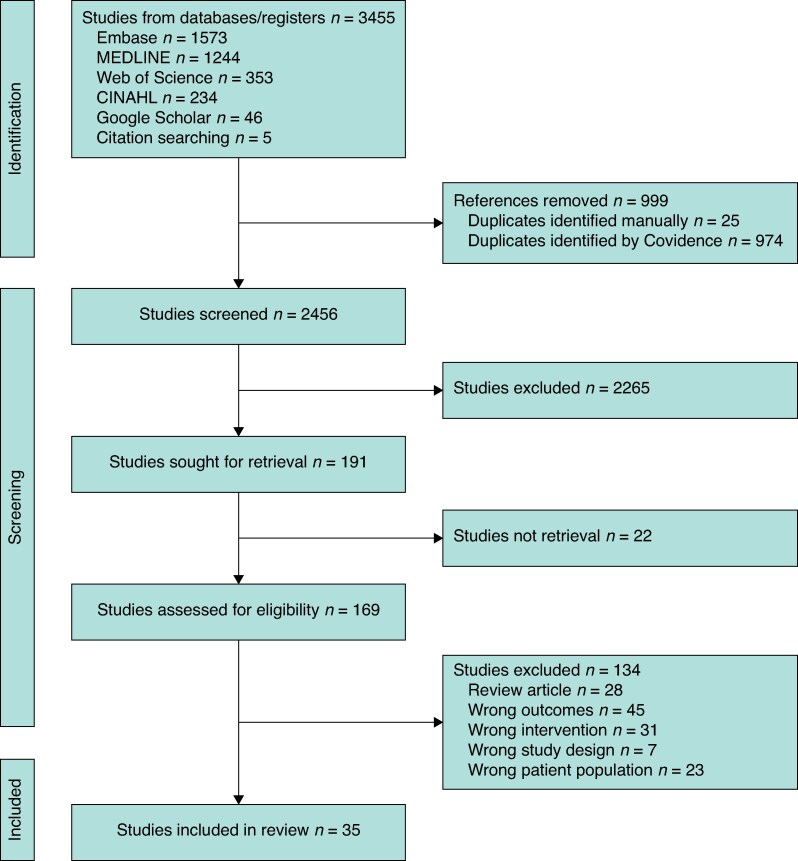
PRISMA flow chart showing selection of articles for review

The systematic search returned 3455 entries for screening and 169 full-text articles were assessed. Most studies were excluded because the intervention or surgical outcomes did not meet the inclusion criteria; 35 studies were included in the review (*Fig. 1*).

Overall, 9378 participants were included in the analyses. All included studies were conducted in high-income countries, with 15^23,25,38–50^ from the USA, 5^22,26,51–53^ from Australia, 3^54–56^ from Canada, 2^57,58^ from the UK, and 1 each from Spain^59^, Denmark^60^, and Finland^61^. Additionally, seven studies^24,62–67^ were based in Japan (*Table 1*).

**Table 1:** Characteristics of studies included in the analysis

Reference country	Year	Elective surgery	Total *n*	Women	Duration of intervention (weeks)	Weight-loss intervention(s)	Outcome measures
Griffin *et al.*^22^ Australia	2024	Gynaecology, ventral hernia repair, cholecystectomy, mixed surgical approach	51	44 (86%)	2–12	VLCD (TDR) *versus* standard care	BL, OT, LOS, SSI, RTED
Griffin *et al.*^26^ Australia	2024	Gynaecology, general, colorectal, upper gastrointestinal, mixed surgical approach	141	107 (75.9%)	13	LCD (TDR)	BL, OT, LOS, SSI, RTED
Hamilton-Reeves *et al.*^38^ USA	2021	Radical prostatectomy, robot-assisted	20	0 (0%)	8	LCD (PDR) *versus* standard care	BL, OT, LOS, non-SSI SSO
Liang *et al*.^39^ USA	2022	Ventral hernia repair, laparoscopic	118	83 (70.3%)	Up to 26	Multifactorial, interdisciplinary rehabilitation *versus* standard counselling	OT, LOS, non-SSI SSO, SSI
de Luis *et al.*^59^ Spain	2012	Hip and knee arthroplasty	40	33 (83%)	19(12)*	LCD (PDR) *versus* standard care	OT, LOS, SSI
Adrados *et al.*^40^ USA	2023	Total knee arthroplasty	700	n.r.	up to 52	Consultation	RTED
Rosen *et al.*^41^ USA	2015	Complex incisional hernia repair, laparotomy	25	20 (80%)	74(33)*†	VLCD	OT, Non-SSI SSO, SSI
Kashihara *et al.*^62^ Japan	2021	Colorectal resection, laparoscopic	120	47 (39.2%)	4(3)	LCD + exercise *versus* no intervention	OT, LOS, CD
Sun *et al.*^42^ USA	2017	Hernia repair, laparoscopic and laparotomy	414	4 (1.0%)	32(21)*†	Consultation *versus* no intervention	LOS, RTED
Burnand *et al.*^57^ UK	2016	Cholecystectomy, laparoscopic	46	42 (91%)	2	VLCD *versus* standard care	OT, LOS, Non-SSI SSO
Wilson *et al.*^51^ Australia	2020	Radical prostatectomy, robot-assisted	43	0 (0%)	4(1)*	LCD + exercise	Non-SSI SSO
Maruyama *et al.*^64^ Japan	2021	Oesophagectomy or gastrectomy, endoscopic surgery	5	n.r.	4(1)*	LCD	BL, OT, LOS, non-SSI SSO, CD
Liljensøe *et al.*^60^ Denmark	2019	Total knee arthroplasty	76	54 (71%)	8	VLCD *versus* standard care	Non-SSI SSO, RTED
Aubrey *et al.*^54^ Canada	2021	Gynaecology, laparoscopic and laparotomy	49	49 (100%)	12–26	LCD + pharmacological (AOM)	BL, OT, LOS
Saito *et al.*^65^ Japan	2023	Hepatectomy, laparotomy	32	8 (25%)	3(2)*	LCD + exercise *versus* no intervention	BL, OT, LOS, CD, BT
Rechenmacher *et al.*^23^ USA	2024	Total knee arthroplasty	90	68 (76%)	Up to 78†	VLCD or LCD + exercise	RTED
Kashihara *et al.*^63^ Japan	2021	Gastrectomy, laparoscopic	22	6 (27%)	4(1)*	LCD + exercise *versus* no intervention	BL, OT, LOS, CD
Lingamfelter *et al.*^43^ USA	2020	Total joint arthroplasty	133	80 (60.2%)	18(17)*	Consultation	Non-SSI SSO
Turcotte *et al.*^25^ USA	2024	Hiatal hernia repair, laparoscopic	134	104 (77.6%)	2	LCD (TDR)	OT, LOS, SSI, non-SSI SSO
Barth *et al.*^44^ USA	2019	Partial hepatectomy, laparotomy	60	27 (45%)	1	VLCD *versus* standard care	BL, OT, LOS, BT
Hollis *et al.*^52^ Australia	2020	Laparoscopic cholecystectomy, hernia repair, laparoscopic	46	29 (63%)	8	VLCD *versus* standard care	OT, LOS, SSI
Yoshiya *et al.*^24^ Japan	2024	Living-donor liver transplantation, laparotomy	63	22 (35%)	Donor: 10(6)* Recipient: 14(25)*†	Donors: LCD + exercise Recipients: exercise	BL, OT
Ssentongo *et al.*^45^ USA	2020	Complex ventral hernia repair, laparotomy	230	132 (57.4%)	14	Consultation	OT, LOS, non-SSI SSO
Maskal *et al.*^46^ USA	2022	Hernia repair, laparoscopic and laparotomy	191	125 (65.4%)	20(10)*†	Consultation *versus* no intervention	LOS, non-SSI SSO, SSI, BT, RTED
Inoue *et al.*^66^ Japan	2019	Gastrectomy, laparoscopic	33	7 (21%)	3	PDR	BL, OT, non-SSI SSO, CD
Imai *et al.*^67^ Japan	2021	Gynaecology, laparoscopic	16	16 (100%)	6(2)*†	LCD + exercise	Non-SSI SSO, SSI
Doyle *et al.*^55^ Canada	2016	Living donor liver transplantation, laparotomy	69	40 (58%)	8(3)*	LCD (TDR) *versus* no intervention	LOS, SSO, CD
Pekkarinen and Mustajoki^61^ Finland	1997	Hernia repair, gynaecology, total arthroplasty, *et al*, mixed surgical approach	30	18 (60%)	14(4)*	VLCD (TDR)	Non-SSI SSO, SSI
McKechnie *et al.*^56^ Canada	2024	Colorectal resection	190	88 (46.3%)	3.4(1)*	LCD (TDR) *versus* no intervention	Non-SSI SSO, SSI, CD
Koutoukidis *et al.*^58^ UK	2025	Colorectal cancer resection	71	28 (39%)	4.7	LCD (TDR) *versus* usual care	OT, LOS, BL, SSO, SSI, non-SSI SSO, CD
Morgan *et al.*^47^ USA	2025	Robotically assisted radical prostatectomy	29	0 (0%)	n.r.	LCD + exercise	OT, BL, LOS, SSO
Spurzem *et al.*^48^ USA	2025	Hernia repair	46	29 (63%)	27.4(17)*	GLP-1 + lifestyle changes *versus* non-use	SSI, CD, reoperation, recurrence
Kim *et al.*^49^, USA	2025	Total joint arthroplasty	5950	3975 (66.8%)	13	GLP-1 *versus* non-use	LOS, SSO, CD
Spurzem *et al.*^50^ USA	2025	Hernia repair	70	46 (66%)	35.6(21)*	GLP-1 + lifestyle changes	SSI, CD, reoperation, recurrence
Ayres *et al.*^53^ Australia	2025	Hysterectomy	25	25 (100%)	5.4	VLCD (TDR)	BL, LOS, CD

Values are *n* (%) unless otherwise stated; *values are mean(s.d.). †If duration of intervention was not reported, the value indicates the time from the start of the intervention to surgery. Dietary terminology was standardized by defining very low-calorie diet (VLCD) as ≤ 800 kcal/day and low-calorie diet (LCD) as 900–1200 kcal/day. Although some studies used different labels, these definitions were applied consistently. Interventions described only as ‘calorie-restricted diet’ without reported caloric intake were classified as LCD. TDR, total diet replacement; BL, blood loss; OT, operating time; LOS, length of hospital stay; SSI, surgical site infection; RETD, return to emergency department; PDR, partial diet replacement; non-SSI SSO, non-infectious surgical site occurrence; n.r., not reported; CD, Clavien–Dindo classification; AOM, Anti-Obesity Medication; BT, blood transfusion; SSO, surgical site occurrence; GLP, glucagon-like peptide.

The mean(s.d.) age of participants was 58(8) years; approximately 61.8% were women, and the mean(s.d.) baseline BMI was 35.6(6.4) kg/m². Diabetes, hypertension, dyslipidaemia, obstructive sleep apnoea, and current smoking affected 46, 53, 41, 18, and 12% of participants among 17, 11, 6, 3, and 11 studies, respectively, that reported on these characteristics (*Table S1*).

Four studies investigated pharmacological weight-loss interventions before elective surgery. One cohort study^49^ compared preoperative glucagon-like peptide (GLP) 1 receptor agonist use *versus* non-use in patients with morbid obesity undergoing primary total knee arthroplasty. Two studies examined GLP-1 receptor agonists prescribed for preoperative weight loss alongside lifestyle-change interventions in patients awaiting elective hernia repair, one comparative^48^ and one single-arm^50^ study. Another study^54^ evaluated a BWLP combined with weight-management medication in women with obesity undergoing gynaecological oncology procedures (mostly GLP-1 analogues and less often extended release naltrexone–bupropion). All other studies focused exclusively on BWLPs alone. Eight RCTs^22,39,44,52,57–60^ and seven non-RCTs^38,42,46,56,62,63,65^ tested BWLPs against usual care or placebo, whereas the remaining studies were single-arm trials assessing BWLPs before operation. Among BWLPs, nine studies^26,41,44,52,53,55,57,60,61^ tested VLCD or TDR, and 16^22,24,25,38,47,51,54,56,58,59,62–67^ tested LCD or PDR with or without an exercise component. The remainder tested general weight-loss dietary advice. (*Tables S1* and *S2*). The median duration of intervention was 8 (interquartile range 4–14) weeks.

Nineteen studies examined specific surgical procedures, including hernia repair (8)^25,39,41,42,45,46,48,50^, liver resection or transplantation (4)^24,44,55,65^, and gastrointestinal resections (7), comprising gastrectomy or oesophagectomy (3)^63,64,66^, laparoscopic cholecystectomy (1)^57^, or colorectal resection (3)^56,58,62^. Orthopaedic procedures, specifically knee or hip arthroplasty, were investigated in six studies^23,40,43,49,59,60^. Gynaecological procedures^53,54,67^ and prostatectomy^38,47,51^ were each examined in 3 studies. The remaining four studies assessed a range of elective procedures: one^52^ included both cholecystectomies and hernia repairs , and the other three^22,26,61^ encompassed a wide variety of procedures across general, orthopaedic, gynaecological, and cardiovascular specialties. The number of participants per surgical type ranged from 80 for gastrointestinal resections to 6920 for orthopaedic procedures, including those from studies that reported multiple procedures (*Table 1* and *Table S3*).

Among the 29 studies, operating time was the most frequently reported intraoperative outcome (21, including 12 comparative studies), followed by blood loss (14, including 7 comparative studies). Among the 14 studies reporting blood loss, 11 involved cancer-related operations, whereas 3 focused on hepatectomy. Postoperative outcome reporting varied widely, with LOS being the most commonly assessed (23 studies), followed by SSIs (13) and non-infectious wound complications (12). The Clavien–Dindo classification was originally reported in 12 studies, and return to the emergency department in 6 (*Table 1*)

### Intraoperative and postoperative outcomes

**Fig. 2:**
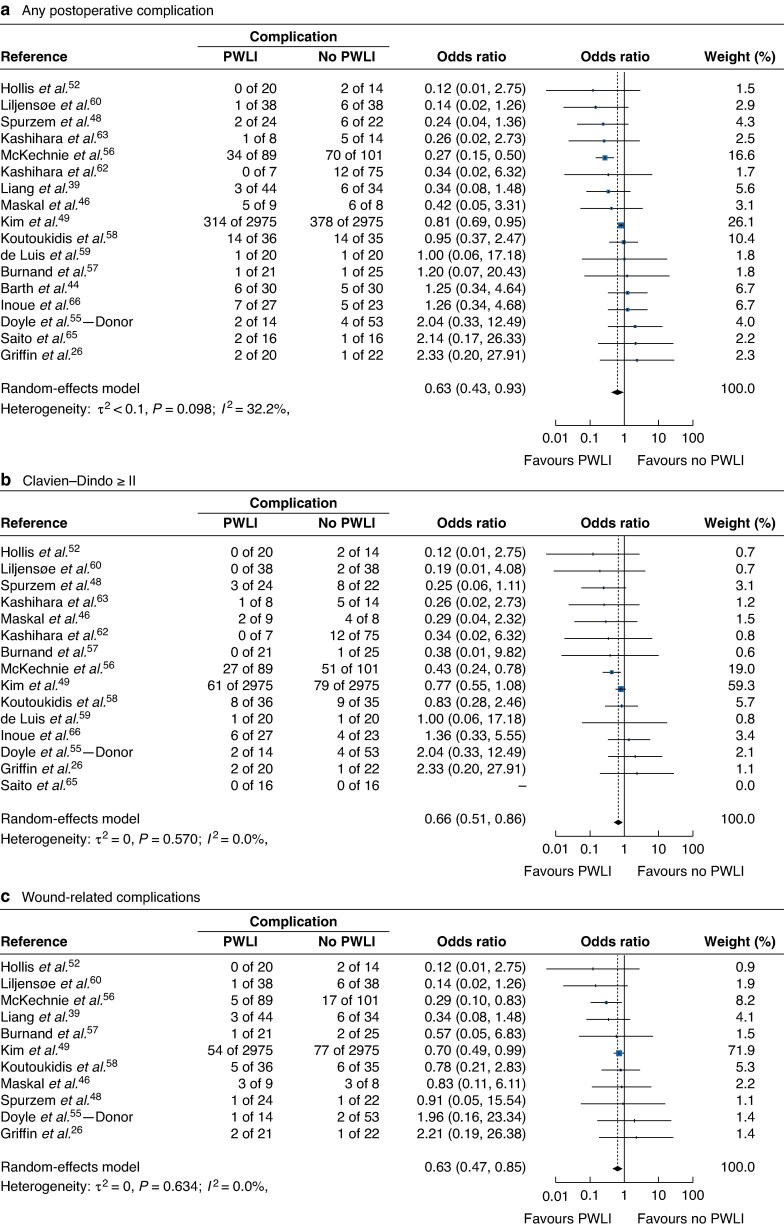
Association between weight-loss interventions and postoperative complications **a** Any postoperative complication, **b** complications requiring medical intervention graded as Clavien–Dindo ≥ II, and **c** wound-related complications. Odds ratios are shown with 95% confidence intervals. PWLI, preoperative weight-loss intervention.

**Fig. 3:**
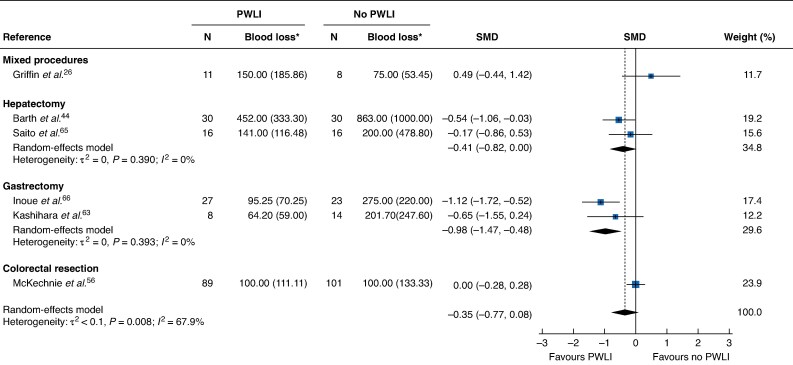
Association between weight-loss interventions and intraoperative blood loss *Values are mean(s.d.). Standardized mean differences (SMDs) are shown with 95% confidence intervals. PWLI, preoperative weight-loss intervention.

There was imprecise evidence that preoperative weight-loss interventions were associated with a statistically significant reduction in overall postoperative complications (odds ratio (OR) 0.63, 95% confidence interval (c.i.) 0.43 to 0.93; *I*² = 32%, 17 studies) (*Fig. 2a*) and in complications requiring medical intervention graded as Clavien–Dindo ≥ II (OR 0.66, 0.51 to 0.86; *I*² = 0%, 15 studies) (*Fig. 2b*). Additionally, a consistent but imprecise effect was observed for any wound-related complications (OR 0.63, 0.47 to 0.85; *I²* = 0%, 11 studies) (*Fig. 2c*) and non-infectious wound-related complications (OR 0.38, 0.15 to 0.97; *I*² = 0%, 6 studies) (*Fig. S1*). Overall, there was no evidence of an effect of weight-loss interventions on blood loss (SMD −0.35, 95% c.i. −0.77 to 0.08). However, subgroup analyses by surgical type showed evidence of a reduction in intraoperative blood loss in both gastrectomy (SMD −0.98, −1.47 to −0.48) and hepatectomy (SMD −0.41, −0.82 to 0.00) (*Fig. 3*). A significant reduction in operating time was reported in one study of cholecystectomy (SMD −1.26, −1.90 to −0.62) (Fig. S1). No clear evidence of association was found for operating time when data were pooled across surgical types for operating time (SMD −0.08, −0.35 to 0.19; *I*² = 60%, 12 studies), but a reduction in blood transfusion was noted (OR 0.49, 0.31 to 0.79; *I²* = 0%, 4 studies) (*Figs S2* and *S3*).

No evidence of improvement was found for other postoperative outcomes, including SSI (OR 0.70, 0.34 to 1.46; *I²* = 8%, 6 studies), Clavien–Dindo grade ≥ III complications (OR 0.67, 0.17 to 2.60; *I²* = 0%, 5 studies), venous thromboembolism (OR 0.85, 0.53 to 1.35; *I²* = 0%, 6 studies), and return to the emergency department (OR 0.56, 0.12 to 2.55; *I²* = 0%, 4 studies). Statistically significant reductions in hospital readmission rates (OR 0.57, 0.47 to 0.70; *I²* = 0%, 6 studies) and LOS (SMD −0.08, −0.13 to −0.04; *I²* = 0%, 15 studies) were noted (*Figs S4*–*S9*).

In single-arm studies, intraoperative outcomes included pooled standardized mean blood loss of 1.12 (95% c.i. 1.00 to 1.24; *I²* = 19%, 15 studies), with pooled standardized means ranging from 0.81 to 2.14. The pooled standardized mean operating time was 3.27 (2.70 to 3.84; *I²* = 100%, 23 studies), with pooled standardized means ranging from 1.56 to 6.75 (*Figs S10* and *S11*). Postoperative outcomes showed a pooled proportion of non-infectious wound-related complications of 0.03 (95% c.i. 0.02 to 0.05; *I²* = 0%, 12 studies), SSI of 0.04 (0.02 to 0.09; *I²* = 0%, 14 studies), and wound-related complications of 0.07 (0.04 to 0.11; *I²* = 92%, 20 studies). The pooled standardized mean LOS was 1.86 (1.16 to 2.56; *I²* = 100%, 23 studies) (*Figs S12*–*S15*).

### Change in weight and body composition

**Fig. 4:**
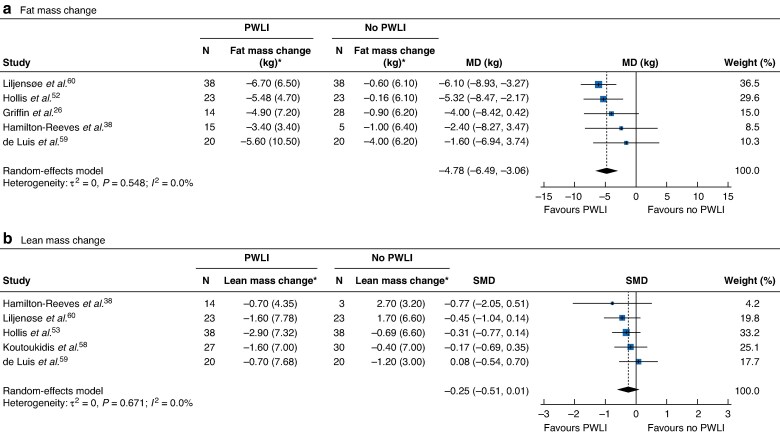
Association between weight-loss intervention and fat mass and lean mass change **a** Fat mass change and **b** lean mass change. *Values are mean(s.d.). Mean differences (MDs) and standardized mean differences (SMDs) are shown with 95% confidence intervals. PWLI, preoperative weight-loss intervention.

Compared with standard care or no intervention, weight-loss interventions were associated with greater weight change (MD −3.92 (95% c.i. −5.44 to −2.39) kg; *I^2^* = 91%, 12 studies), ranging from −0.77 kg^39^ to −9.47 kg^60^ across 12 comparative studies (*Fig. S16*). Weight-loss interventions led to more fat mass loss (MD −4.78 (−6.49 to −3.06) kg; *I^2^* = 0%, 5 studies), whereas no statistically significant changes were observed in lean mass indices (SMD −0.25, 95% c.i. −0.51 to 0.01; *I^2^* = 0%, 5 studies) (*Fig. 4*).

Among the five comparative studies, lean mass was reported as non-bone lean body mass^38^, fat-free mass^58,59^, muscle mass^52^, lean mass^60^, and appendicular skeletal muscle mass^51^. Additionally, one single-arm study^66^ reported skeletal muscle. Weight-loss interventions also led to significantly greater reductions in waist circumference (MD −4.96 (95% c.i. −8.43 to −1.49) cm; *I*^2^ = 85%, 5 studies) (*Fig. S17*). In the single-arm analysis of weight change, 34 arms from 32 studies demonstrated significant weight loss from baseline to surgery (difference in mean −7.38 (−8.53 to −6.24) kg; *I²* = 98%) (*Fig. S18*). Nine study arms demonstrated a significant reduction in fat mass (difference in mean −4.18 (−5.75 to −2.62) kg; *I²* = 95%) and waist circumference (difference in mean −5.70 (−7.23 to −4.16) cm; *I²* = 92%) from baseline to surgery (*Figs S19* and *S20*). Five arms showed a significant change in lean mass (standardized mean change −0.20, 95% c.i. −0.26 to −0.15; *I²* = 0%) (*Fig. S21*).

### Exploratory analysis

**Fig. 5:**
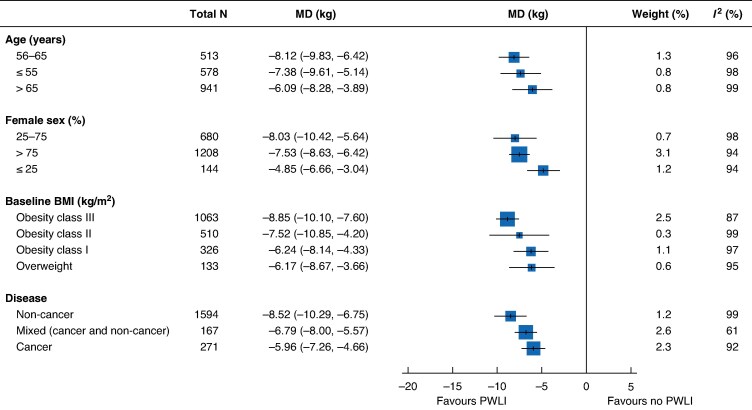
Forest plot showing subgroup analyses of weight change by age, sex, baseline BMI, and cancer status Mean differences (MD) are shown with 95% confidence intervals. BMI, body mass index; PWLI, preoperative weight-loss intervention.

In *post hoc* subgroup analysis, weight loss differed significantly by the proportion of female participants (*P* = 0.030), with studies including a lower proportion of women (≤ 25%) showing smaller reductions in bodyweight (*Fig. 5* and *Fig. S22*). There was no evidence that weight loss differed based on BMI, age, or cancer status (*Figs S23*–*S25*).

**Fig. 6:**
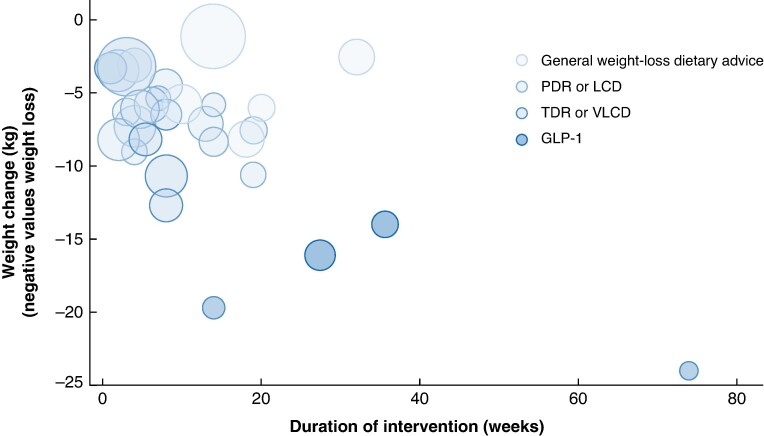
Meta-regression of weight change on duration of intervention Each circle represents an individual study, with size proportional to its relative weight in the meta-analysis. *Negative values indicate weight loss. Weight change (kg) = –0.21 × duration of intervention (weeks) – 0.68. PDR, partial diet replacement; LCD, low-calorie diet; TDR, total diet replacement; VLCD, very low-calorie diet; GLP, glucagon-like peptide.

*Post hoc* meta-regression analysis revealed a significant association between longer duration of intervention and greater weight loss, with every additional week of the intervention being associated with an additional weight change of −0.21 (95% c.i. −0.33 to −0.09) kg (*P* < 0.001) (*Fig. 6* and *Table S4*). Compared with general weight-loss dietary advice, GLP-1 intervention achieved significantly greater weight loss (β = −7.76 kg, 95% c.i. −12.98 to −2.53; *P* = 0.004), followed by TDR or VLCD (β = −6.37 kg, −9.90 to −2.85; *P* < 0.001), and then PDR or LCD (β = −4.18 kg, −7.48 to −0.87; *P* = 0.013) after adjusting for duration of intervention (*Table S4*).

In subgroup analyses, weight-loss interventions were associated with a significant reduction in overall postoperative complications among less invasive procedures, including laparoscopic and robotic surgery (OR 0.48, 95% c.i. 0.27 to 0.84; *I²* = 21%, 8 studies), but no association was observed in more invasive procedures, namely laparotomy (OR 0.87, 0.39 to 1.98; *I²* = 6%, 5 studies) and joint arthroplasty (OR 0.68, 0.23 to 1.46; *I²* = 18%, 3 studies; *Fig. S26*).

### Risk of bias and sensitivity analyses

*Table S5* shows a summary of the risk-of-bias assessments. Six studies were judged to be at low risk of bias across all domains, 14 as moderate, and 15 as high risk. In an analysis limited to studies with a low risk of bias, operating time remained non-significant, whereas LOS no longer showed a statistically significant reduction (*Figs S27* and *S28*). For intraoperative blood loss, the overall effect estimate remained non-significant (SMD −0.47, 95% c.i. −1.24 to 0.31; 3 studies), but subgroup analyses showed strengthened associations. The effect in gastrectomy increased in magnitude (SMD from −0.98 to −1.12), and the association in hepatectomy became statistically significant (SMD from −0.41 to −0.54) (*Fig. S29*). For weight change, the pooled estimate shifted from −3.92 to −5.42 kg (*Fig. S30*). Subgroup analysis of risk of bias in single-arm studies indicated that studies with a low risk of bias reported less weight loss than those with a moderate or high risk (*Fig. S31*).

On examination of funnel plots for blood loss, LOS, and weight change (*Fig. S32*), there was no evidence of publication bias. In contrast, statistical evidence of publication bias was detected for operating time (*P* = 0.030). A formal Grading of Recommendations Assessment, Development and Evaluation assessment was not performed but, based on study quality and heterogeneity, the certainty of the evidence was generally considered to be low to moderate.

## Discussion

This systematic review and meta-analysis demonstrated that adults who are overweight or obese awaiting elective non-bariatric surgery can have weight-loss interventions that lead primarily to fat loss without concomitant loss of lean mass. They are associated with clinically meaningful reductions in postoperative complications and complications requiring medical intervention. Additionally, there was evidence of reductions in wound-related complications, non-infectious wound complications, blood transfusion, hospital readmissions, and LOS. Subgroup analyses by surgical type revealed evidence of significant reductions in intraoperative blood loss in patients undergoing gastrectomy and hepatectomy. There was no evidence that weight-loss interventions were associated with reductions in other surgical complications, such as SSI, venous thromboembolism, or return to the emergency department. Fifteen studies were assessed as having a high risk of bias, whereas 14 were considered to have a moderate risk. Nevertheless, sensitivity analyses indicated that excluding studies with high and moderate risk of bias did not substantially alter the estimates.

Cochrane systematic review methodologies were adhered to, ensuring robust study selection and data synthesis. Inclusion of both RCTs and well designed observational studies balanced internal validity (RCTs) and external validity. This approach minimized data loss and inconsistencies, allowing inclusion of the most complete data set possible from the original studies. Although the inclusion of non-randomized designs may have introduced bias, sensitivity analyses excluding studies with a moderate or high risk of bias revealed consistent results, supporting the findings. The data set incorporated 9378 participants from 8 high-income countries. However, the absence of studies from low- and middle-income settings precludes the generalizability of the findings to low-resource settings.

The association between preoperative weight loss and a reduction in overall postoperative complications, including those graded as Clavien–Dindo > II, reflects a clinically relevant reduction of surgical risk in patients with overweight/obesity. Complications necessitating medical or procedural intervention, as captured by the Clavien–Dindo classification, are particularly meaningful from both patient-centred and health system perspectives. Intentional weight loss before surgery may reduce systemic inflammation, and improve surgical access and visualization^11,12^, thereby enhancing postoperative recovery and reducing the incidence of events requiring pharmacological, endoscopic, or surgical management. These benefits appeared more evident in less invasive procedures, whereas no clear association was observed for more invasive operations, which may reflect differences in complication profiles and the limited data available. Although these mechanisms are biologically plausible and supported by previous studies, existing evidence has been largely derived from bariatric surgery populations^21^. The findings extend this evidence base by demonstrating similar benefits in non-bariatric elective procedures, thereby strengthening the rationale for preoperative weight optimization across a broader range of surgical contexts.

The observed reduction in any wound complications and in non-infectious wound complications following preoperative weight-loss interventions is consistent with known impairments in wound healing among patients with obesity^12^. This reduction may reflect mitigation of the inflammatory status typically seen in obesity following weight-loss interventions. However, the meta-analysis did not provide evidence that preoperative weight-loss interventions reduce the risk of SSI or venous thromboembolism, both of which have been well documented to occur at higher rates in patients with obesity compared with those of healthy weight^5,9,10^.

Despite a non-significant overall effect size, subgroup analyses demonstrated significant reductions in intraoperative blood loss in gastrectomy and hepatectomy, based on five comparative studies, including four cancer-related procedures. Minimizing blood loss is critical in oncological surgery, as increased blood loss heightens the risk of blood transfusion, which may induce immunosuppression and facilitate malignant cell dissemination^11^. Weight-loss interventions have been shown to reduce visceral fat, thereby facilitating dissection and the visualization of key anatomical structures, such as Calot’s triangle in laparoscopic cholecystectomy^57^. Additionally, robust evidence indicates that weight-loss interventions are associated with reduced liver steatosis^68^, potentially leading to improved liver manoeuvrability^44^. These benefits are particularly relevant for abdominal operations, where enhanced liver retraction, mobilization, and manipulation can contribute to technical ease, potentially reducing both blood loss and operating time^11^.

The findings have shown that intentional weight-loss interventions in this population lead to weight loss that is primarily driven by reductions in excess fat mass. Although body composition outcomes were not reported consistently, pooled analysis of five studies found no statistically significant association between weight-loss interventions and changes in lean mass. This, coupled with a lack of evidence for worsening of any surgical outcome and some improvements, should provide reassurance that weight-loss interventions do not unintentionally harm this population.

In the US, the American Association of Hip and Knee Surgeons^40^ recommends preoperative weight loss for patients with class III obesity before total knee arthroplasty to reduce perioperative complications. The present results suggest that those with less severe obesity also lose a clinically meaningful amount of weight and therefore may observe similar benefits in surgical outcomes. Furthermore, the results presented support the recent UK guidelines^69^ suggesting that clinicians should consider very low-energy diets for people with obesity who need to lose weight rapidly, so that they can make surgery safer. Clinicians may use these findings to counsel patients on the potential surgical benefits of preoperative weight loss and offer them effective interventions.

This study has limitations, including heterogeneity in study designs and surgical populations. Although the review included weight-loss interventions that were heterogeneous in terms of intensity, duration, and weight loss achieved, an assumption was made that weight loss was the primary factor influencing surgical outcomes. Sensitivity analyses to explore the type of intervention or the impact of baseline BMI were only possible for weight loss as outcome. SMDs were pooled where possible to account for the multiple different types of surgery performed and comparisons were made with usual care. The substantial variation in baseline BMI across studies likely reflects the broad spectrum of elective procedures and could be investigated further with individual-participant data meta-analyses. Despite these differences, the overall relative reduction in complications remained significant, with low heterogeneity (*I²* = 32% for any postoperative complication, *I²* = 0% for Clavien–Dindo grade ≥ II complications). Among the six studies published in 2025, three evaluated GLP-1-based weight-loss interventions before elective surgery, indicating that pharmacological preoperative pathways are rapidly emerging. Most of the included BWLPs varied in intensity and format, including total or partial meal replacements, energy-restricted diets, and general dietary advice. Longer and more intensive programmes led to greater weight loss but there were limited data on whether greater weight loss leads to better outcomes.

Evidence remains limited across many surgical specialties, and procedure-specific benefits and risks of preoperative weight loss require further study. The findings of this study support the benefits of preoperative weight loss in reducing postoperative complications. Implementing such interventions before surgery requires time and must consider the clinical context. In colorectal cancer, for example, evidence from a meta-analysis^70^ showed no worsening of outcomes if the interval between diagnosis and surgery was < 6 weeks. The median intervention period in the present review was 8 weeks, suggesting that even a 6-week interval should allow sufficient time for preoperative optimization. Future research should examine the timing and suitable candidate populations for preoperative weight loss across surgical specialties, aiming to maximize potential benefits while minimizing potential risks associated with treatment delay.

Although some pooled estimates were statistically significant, the corresponding 95% confidence intervals were relatively wide and, in some instances, approached the line of no effect. This limited precision in effect estimates likely reflects small sample sizes and low event counts in the included studies. Most trials had small samples and reported only short-term outcomes, with few data beyond hospital discharge. Future studies should include long-term follow-up to assess the durability of weight loss and its association with postoperative and functional outcomes. Future research is required to determine the optimal preoperative weight-loss strategy for specific surgical procedures. Standardized assessment of body composition measures would be welcomed. Large-scale high-quality RCTs in diverse populations are needed to test definitively whether weight-loss interventions improve intraoperative and postoperative outcomes.

## Supplementary Material

zrag001_Supplementary_Data

## Data Availability

Data collection forms and extracted data sets were stored locally and can be provided upon request.
