# Supplementary material for: Impact of preoperative weight-loss interventions on outcomes after elective non-bariatric surgery: meta-analysis
Source: BJS Open. 2026 Feb 25;10(1):zrag001. doi: 10.1093/bjsopen/zrag001 (PMC13223571; doi:10.1093/bjsopen/zrag001)
Supplement: zrag001_Supplementary_Data [file zrag001_supplementary_data.docx]

**The impact of preoperative weight loss interventions on outcomes after elective non-bariatric surgery: a systematic review and meta-analysis**

Danni Wang^1,2^*, Simon J.A. Buczacki ^3^, Qiufeng Gu^1^, Zhengmei Liao^1^, Yanli Jiang^1^, Sam West^2^, Dimitrios A Koutoukidis^2^*

^1^ Department of Health Promotion and Behaviour Sciences, Anhui Medical University, Hefei, China

^2^ Nuffield Department of Primary Care Health Sciences, University of Oxford, Oxford, United Kingdom

^3^ Nuffield Department of Surgical Sciences, University of Oxford, Oxford, UK

*Correspondence: Danni Wang, Ph.D., Department of Health Promotion and Behavioral Sciences, School of Public Health, Anhui Medical University, Hefei 230032, Anhui, China; E-mail: wangdanni@ahmu.edu.cn; Telephone/Fax: +86-551-65161282.

* These authors contributed equally as co-corresponding authors

Supplementary Materials – Index

**Supplementary Methods**

| Methods S 1 Search strategy | [***page 4-15***](#Meth) |
| --- | --- |

**Supplementary Figures and Tables**

| Supplementary Table 1 | [*page 16-17*](#Ts1) |
| --- | --- |
| Supplementary Table 2 | [*page 18-21*](#Ts2) |
| Supplementary Table 3 | [*page 22*](#Ts3) |
| Supplementary Figure 1 | [*page 23*](#Fs1) |
| Supplementary Figure 2 | [*page 23*](#Fs2) |
| Supplementary Figure 3 | [*page 24*](#Fs3) |
| Supplementary Figure 4 | [*page 24*](#Fs4) |
| Supplementary Figure 5 | [*page 25*](#Fs5) |
| Supplementary Figure 6 | [*page 25*](#Fs6) |
| Supplementary Figure 7 | [*page 25*](#Fs8) |
| Supplementary Figure 8 | [*page 26*](#Fs8) |
| Supplementary Figure 9 | [*page 26*](#Fs9) |
| Supplementary Figure 10 | [*page 27*](#Fs10) |
| Supplementary Figure 11 | [*page 27*](#Fs11) |
| Supplementary Figure 12 | [*page 28*](#Fs12) |
| Supplementary Figure 13 | [*page 28*](#Fs13) |
| Supplementary Figure 14 | [*page 29*](#Fs14) |
| Supplementary Figure 15 | [*page 30*](#Fs15) |
| Supplementary Figure 16 | [*page 30*](#Fs16) |
| Supplementary Figure 17 | [*page 31*](#Fs17) |
| Supplementary Figure 18 | [*page 31*](#Fs18) |
| Supplementary Figure 19 | [*page 32*](#Fs19) |
| Supplementary Figure 20 | [*page 32*](#Fs20) |
| Supplementary Figure 21 | [*page 32*](#Fs21) |
| Supplementary Figure 22 | [*page 33*](#Fs22) |
| Supplementary Figure 23 | [*page 34*](#Fs23) |
| Supplementary Figure 24 | [*page 35*](#Fs24) |
| Supplementary Figure 25 | [*page 36*](#Fs25) |
| Supplementary Table 4 | [*page 37*](#Ts4) |
| Supplementary Figure 26 | [*page 37*](#Fs26) |
| Supplementary Table 5 | [*Page38-39*](#Ts5) |
| Supplementary Figure 27 | [*page 40*](#Fs27) |
| Supplementary Figure 28 | [*page 41*](#Fs28) |
| Supplementary Figure 29 | [*page 41*](#Fs29) |
| Supplementary Figure 30 | [*page 42*](#Fs30) |
| Supplementary Figure 31 | [*page 43*](#Fs31) |
| Supplementary Figure 32 | [*page 44*](#Fs32) |
| Supplementary Table 6 | [*page45-47*](#Ts6) |

Methods S 1 Search strategy

**Medline**

| #▲ | Searches |
| --- | --- |
| 1 | body weight changes/ or weight loss/ |
| 2 | body mass index/ or sagittal abdominal diameter/ or exp waist circumference/ or waist-height ratio/ |
| 3 | ((bodyweight or weight) adj3 (loss or losing or lose or lost or reduc* or manage* or control)).ti,ab,kf. |
| 4 | (bmi or body mass index).ti,kf. or ((bmi or body mass index) adj3 (chang* or reduc* or low* or decreas*)).ab. or (waist adj2 (circumference or hip ratio?)).ti,ab,kf. or (abdom* adj circumference).ti,ab,kf. |
| 5 | obesity/ or obesity, morbid/ or obesity, abdominal/ |
| 6 | (obes* or overweight).ti,kf. |
| 7 | 1 or 2 or 3 or 4 or 5 or 6 |
| 8 | diet therapy/ or caloric restriction/ or exp diet, carbohydrate-restricted/ or diet, fat-restricted/ or exp diet, high-protein/ or diet, reducing/ or dietary approaches to stop hypertension/ |
| 9 | exp Obesity Management/ |
| 10 | Obesity/dh, th |
| 11 | Weight reduction programs/ |
| 12 | ((low* fat or reduc* fat? or low* calorie* or reduc* calorie* or low* energy or reduc* energy or hypocalor* or calori* restrict* or calorie control* or low* glyc* or reduc* glyc* or low* carbohydrate? or reduc* carbohydrate? or high* protein? or increas* protein? or paleo* or intermittent fasting or meal replacement? or replacement meal?) adj2 (diet? or plan* or regim* or program* or intervention?)).ti,ab,kf. |
| 13 | ((weight loss or weight control or weight management or weight reduction) adj2 (diet? or plan* or regim* or program* or intervention?)).ti,ab,kf. |
| 14 | exp Anti-Obesity Agents/ |
| 15 | Obesity/dt |
| 16 | ((antiobesity or anti-obesity) adj3 (medication? or medicine? or drug? or agent?)).ti,kf. |
| 17 | (liraglutide or saxenda or victoza or semaglutide or ozempic or wegovy or tirzepatide or exenatide or bydureon or byetta or lixisenatide or albiglutide or tanzeum or eperzan or dulaglutide or trulicity or phentermine or adipex-p or suprenza or topiramate or topamax or qsymia or lorcaserin or belviq or (naltrexone and bupropion) or sibutramine or rimonabant or benzphetamine or diethylpropion or tenuate or phendimetrazine or bontril or orlistat or xenical).ti,ab,kf. |
| 18 | 8 or 9 or 10 or 11 or 12 or 13 or 14 or 15 or 16 or 17 |
| 19 | Preoperative Period/ or Preoperative Care/ |
| 20 | (preoperat* or pre-operat* or presurg* or pre-surg* or preprocedur* or pre-procedur* or (("before" or prior) adj5 (surg* or operat* or procedur*)) or "prior to").ti,ab,kf. |
| 21 | 19 or 20 |
| 22 | surgical procedures, operative/ or exp cardiovascular surgical procedures/ or cytoreduction surgical procedures/ or exp decompression, surgical/ or deep brain stimulation/ or digestive system surgical procedures/ or biliary tract surgical procedures/ or exp cholecystectomy/ or cholecystostomy/ or choledochostomy/ or portoenterostomy, hepatic/ or sphincterotomy, endoscopic/ or sphincterotomy, transduodenal/ or exp colectomy/ or exp enterostomy/ or esophagectomy/ or esophagoplasty/ or esophagostomy/ or fundoplication/ or exp gastrectomy/ or gastropexy/ or gastrostomy/ or heller myotomy/ or hemorrhoidectomy/ or hepatectomy/ or liver transplantation/ or pancreas transplantation/ or pancreatectomy/ or pancreaticoduodenectomy/ or pancreaticojejunostomy/ or peritoneovenous shunt/ or exp proctectomy/ or dissection/ or elective surgical procedures/ or electrosurgery/ or exp endocrine surgical procedures/ or fasciotomy/ or laparotomy/ or exp lymph node excision/ or exp mastectomy/ or metastasectomy/ or exp microsurgery/ or exp minimally invasive surgical procedures/ or minor surgical procedures/ or exp myotomy/ or exp obstetric surgical procedures/ or exp neurosurgical procedures/ or exp ophthalmologic surgical procedures/ or exp oral surgical procedures/ or exp orthopedic procedures/ or exp ostomy/ or exp otorhinolaryngologic surgical procedures/ or pelvic exenteration/ or exp plastic surgery procedures/ or exp prophylactic surgical procedures/ or exp prosthesis implantation/ or splenectomy/ or exp surgery, computer-assisted/ or symphysiotomy/ or exp thoracic surgical procedures/ or exp transplantation/ or exp urogenital surgical procedures/ |
| 23 | (surg* or operat* or procedure?).ti,kf. |
| 24 | ((nonbariatric or non-bariatric) adj2 (surg* or procedure?)).ab. |
| 25 | 22 or 23 or 24 |
| 26 | 7 and 18 and 21 and 25 |
| 27 | ((preoperat* or pre-operat* or presurg* or pre-surg* or preprocedur* or pre-procedur*) adj5 (weight loss or weight control or weight management or weight reduction)).ti,ab,kf. |
| 28 | 26 or 27 |
| 29 | exp randomized controlled trial/ |
| 30 | clinical trial/ or comparative study/ or multicenter study/ |
| 31 | controlled clinical trial.pt. |
| 32 | randomized.ab. |
| 33 | placebo.ab. |
| 34 | drug therapy.fs. |
| 35 | randomly.ab. |
| 36 | trial.ab. |
| 37 | groups.ab. |
| 38 | observational study/ |
| 39 | cohort studies/ or follow-up studies/ or longitudinal studies/ or prospective studies/ or retrospective studies/ |
| 40 | (((cohort or prospective or retrospective or "follow up" or longitudinal) adj stud*) or cohort analysis).ti,ab,kf. |
| 41 | 29 or 30 or 31 or 32 or 33 or 34 or 35 or 36 or 37 or 38 or 39 or 40 |
| 42 | (baboon$1 or bovine$1 or canine$1 or cat$1 or chimpanzee$1 or cow$1 or dog$1 or feline$1 or goat$1 or hens or macque$1 or mice or monkey$1 or (mouse adj2 model$1) or murine$1 or ovine or pig$1 or porcine or (non-human adj2 primate$1) or sheep or rabbit$1 or rat or rats or rattus or rhesus or rodent$1 or zebrafish).ti. |
| 43 | 41 not 42 |
| 44 | 28 and 43 |
| 45 | (obesity surg* or metabolic surg* or weight loss surg* or gastric sleeve or intragastric sleeve or sleeve gastr* or ileal sleeve or gastric band* or intragastric band* or gastric bypass* or intragastric bypass* or jejuno* bypass* or ileal bypass* or gastric balloon* or intragastric balloon* or roux-en-y or biliopancreatic diversion or bilio-pancreatic diversion or duodenal bypass*).ti. |
| 46 | (bariatric surg* not (nonbariatric or non-bariatric)).ti. |
| 47 | 45 or 46 |
| 48 | 44 not 47 |

**Embase**

| #▲ | Searches |
| --- | --- |
| 1 | body weight change/ or body weight loss/ |
| 2 | anthropometric parameters/ or abdominal circumference/ or body adiposity index/ or body fat percentage/ or body mass/ or hip circumference/ or sagittal abdominal diameter/ or waist circumference/ or waist hip ratio/ or waist to height ratio/ or weight height ratio/ or weight chart/ or bmi chart/ |
| 3 | ((bodyweight or weight) adj3 (loss or losing or lose or lost or reduc* or manage* or control)).ti,ab,kf. |
| 4 | (bmi or body mass index).ti,kf. or ((bmi or body mass index) adj3 (chang* or reduc* or low* or decreas*)).ab. or (waist adj2 (circumference or hip ratio?)).ti,ab,kf. or (abdom* adj circumference).ti,ab,kf. |
| 5 | obesity/ or abdominal obesity/ or morbid obesity/ |
| 6 | (obes* or overweight).ti,kf. |
| 7 | 1 or 2 or 3 or 4 or 5 or 6 |
| 8 | diet therapy/ or exp dash diet/ or exp diet restriction/ or exp low calorie diet/ or low fat diet/ or modified atkins diet/ or exp protein diet/ or exp ketogenic diet/ |
| 9 | Obesity Management/ |
| 10 | obesity/dm, th |
| 11 | weight loss program/ |
| 12 | ((low* fat or reduc* fat? or low* calorie* or reduc* calorie* or low* energy or reduc* energy or hypocalor* or calori* restrict* or calorie control* or low* glyc* or reduc* glyc* or low* carbohydrate? or reduc* carbohydrate? or high* protein? or increas* protein? or paleo* or intermittent fasting or meal replacement? or replacement meal?) adj2 (diet? or plan* or regim* or program* or intervention?)).ti,ab,kf. |
| 13 | ((weight loss or weight control or weight management or weight reduction) adj2 (diet? or plan* or regim* or program* or intervention?)).ti,ab,kf. |
| 14 | exp antiobesity agent/ |
| 15 | obesity/dt |
| 16 | ((antiobesity or anti-obesity) adj3 (medication? or medicine? or drug? or agent?)).ti,kf. |
| 17 | (liraglutide or saxenda or victoza or semaglutide or ozempic or wegovy or tirzepatide or exenatide or bydureon or byetta or lixisenatide or albiglutide or tanzeum or eperzan or dulaglutide or trulicity or phentermine or adipex-p or suprenza or topiramate or topamax or qsymia or lorcaserin or belviq or (naltrexone and bupropion) or sibutramine or rimonabant or benzphetamine or diethylpropion or tenuate or phendimetrazine or bontril or orlistat or xenical).ti,ab,kf. |
| 18 | 8 or 9 or 10 or 11 or 12 or 13 or 14 or 15 or 16 or 17 |
| 19 | preoperative care/ or preoperative period/ or preoperative exercise/ |
| 20 | (preoperat* or pre-operat* or presurg* or pre-surg* or preprocedur* or pre-procedur* or (("before" or prior) adj5 (surg* or operat* or procedur*)) or "prior to").ti,ab,kf. |
| 21 | 19 or 20 |
| 22 | surgery/ or exp breast surgery/ or exp cancer surgery/ or exp cardiovascular surgery/ or exp ear nose throat surgery/ or elective surgery/ or general surgery/ or geriatric surgery/ or exp "head and neck surgery"/ or major surgery/ or minimally invasive surgery/ or minor surgery/ or exp neurosurgery/ or open surgery/ or exp orthopedic surgery/ or exp pelvis surgery/ or exp plastic surgery/ or reconstructive surgery/ or exp thorax surgery/ or exp transplantation/ or exp urologic surgery/ or gastrointestinal surgery/ or exp antireflux operation/ or exp anus surgery/ or exp intestine surgery/ or exp pancreas surgery/ or exp stomach surgery/ or exp vagotomy/ or abdominal surgery/ or abdominal wall closure/ or exp biliary tract surgery/ or hernioplasty/ or herniorrhaphy/ or herniotomy/ or exp laparoscopy/ or laparotomy/ or exp liver surgery/ or omentectomy/ or omentoplasty/ or peritoneum lavage/ or exp spleen surgery/ or exp endocrine surgery/ |
| 23 | (surg* or operat* or procedure?).ti,kf. |
| 24 | ((nonbariatric or non-bariatric) adj2 (surg* or procedure?)).ab. |
| 25 | 22 or 23 or 24 |
| 26 | 7 and 18 and 21 and 25 |
| 27 | ((preoperat* or pre-operat* or presurg* or pre-surg* or preprocedur* or pre-procedur*) adj5 (weight loss or weight control or weight management or weight reduction)).ti,ab,kf. |
| 28 | 26 or 27 |
| 29 | exp randomized controlled trial/ |
| 30 | Controlled clinical trial/ |
| 31 | random$.ti,ab. |
| 32 | randomization/ |
| 33 | intermethod comparison/ |
| 34 | placebo.ti,ab. |
| 35 | (compare or compared or comparison).ti. |
| 36 | ((evaluated or evaluate or evaluating or assessed or assess) and (compare or compared or comparing or comparison)).ab. |
| 37 | (open adj label).ti,ab. |
| 38 | ((double or single or doubly or singly) adj (blind or blinded or blindly)).ti,ab. |
| 39 | double blind procedure/ |
| 40 | parallel group$1.ti,ab. |
| 41 | (crossover or cross over).ti,ab. |
| 42 | [((assign$ or match or matched or allocation) adj5 (alternate or group$1 or intervention$1 or patient$1 or subject$1 or participant$1)).ti,ab.](https://ezproxy-prd.bodleian.ox.ac.uk:2483/ovid-a/ovidweb.cgi?&S=DGLOFPGFPGEBIFGIJPPJBHBFJPFIAA00&R=30&Search+Annotations+Options=SA) |
| 43 | [(assigned or allocated).ti,ab.](https://ezproxy-prd.bodleian.ox.ac.uk:2483/ovid-a/ovidweb.cgi?&S=DGLOFPGFPGEBIFGIJPPJBHBFJPFIAA00&R=32&Search+Annotations+Options=SA) |
| 44 | (controlled adj7 (study or design or trial)).ti,ab. |
| 45 | (volunteer or volunteers).ti,ab. |
| 46 | human experiment/ |
| 47 | trial.ti. |
| 48 | Clinical study/ or Case control study/ or Family study/ or Longitudinal study/ or Retrospective study/ or Prospective study/ or Cohort analysis/ |
| 49 | (((Cohort or case control or "follow up" or observational or epidemiologic*) adj (study or studies)) or cohort analysis).ti,ab,kf. |
| 50 | or/29-49 |
| 51 | (random$ adj sampl$ adj7 ("cross section$" or questionnaire$1 or survey$ or database$1)).ti,ab. not (comparative study/ or controlled study/ or randomi?ed controlled.ti,ab. or randomly assigned.ti,ab.) |
| 52 | Cross-sectional study/ not (exp randomized controlled trial/ or controlled clinical study/ or controlled study/ or randomi?ed controlled.ti,ab. or control group$1.ti,ab.) |
| 53 | Systematic review.ti,ab. not (trial or study).ti. |
| 54 | (nonrandom$ not random$).ti,ab. |
| 55 | random field$.ti,ab. |
| 56 | (random cluster adj3 sampl$).ti,ab. |
| 57 | (review.ab. and review.pt.) not trial.ti. |
| 58 | we searched.ab. and (review.ti. or review.pt.) |
| 59 | update review.ab. |
| 60 | (databases adj4 searched).ab. |
| 61 | (rat or rats or mouse or mice or swine or porcine or murine or sheep or lambs or pigs or piglets or rabbit or rabbits or cat or cats or dog or dogs or cattle or bovine or monkey or monkeys or trout or marmoset$1).ti. and animal experiment/ |
| 62 | Animal experiment/ not (human experiment/ or human/) |
| 63 | or/51-62 |
| 64 | 50 not 63 |
| 65 | 28 and 64 |
| 66 | (obesity surg* or metabolic surg* or weight loss surg* or gastric sleeve or intragastric sleeve or sleeve gastr* or ileal sleeve or gastric band* or intragastric band* or gastric bypass* or intragastric bypass* or jejuno* bypass* or ileal bypass* or gastric balloon* or intragastric balloon* or roux-en-y or biliopancreatic diversion or bilio-pancreatic diversion or duodenal bypass*).ti. |
| 67 | (bariatric surg* not (nonbariatric or non-bariatric)).ti. |
| 68 | 66 or 67 |
| 69 | 65 not 68 |

**CINAHL**

| #▲ | Searches |
| --- | --- |
| S17 | S15 NOT S16 |
| S16 | ((TI "obesity surg*") OR (TI "metabolic surg*") OR (TI "weight loss surg*") OR (TI "gastric sleeve") OR (TI "intragastric sleeve") OR (TI "sleeve gastr*") OR (TI "ileal sleeve") OR (TI "gastric band*") OR (TI "intragastric band*") OR (TI "gastric bypass*") OR (TI "intragastric bypass*") OR (TI "jejuno* bypass*") OR (TI "ileal bypass*") OR (TI "gastric balloon*") OR (TI "intragastric balloon*") OR (TI roux-en-y) OR (TI "biliopancreatic diversion") OR (TI "bilio-pancreatic diversion") OR (TI "duodenal bypass*")) or ((TI "bariatric surg*") NOT ((TI nonbariatric) OR (TI non-bariatric))) |
| S15 | S13 OR S14 |
| S14 | (((TX preoperat*) OR (TX pre-operat*) OR (TX presurg*) OR (TX pre-surg*) OR (TX preprocedur*) OR (TX pre-procedur*)) N5 ((TX "weight loss") OR (TX "weight control") OR (TX "weight management") OR (TX "weight reduction"))) |
| S13 | S3 AND S6 AND S9 AND S12 |
| S12 | S10 OR S11 |
| S11 | ((TI surg*) OR (TI operat*) OR (TI procedure#)) OR (((AB nonbariatric) OR (AB non-bariatric)) N2 ((AB surg*) OR (AB procedure#))) |
| S10 | (MH "Surgery, Operative") OR (MH "Ablation Techniques") OR (MH "Arteriovenous Shunt, Surgical") OR (MH "Cerebrospinal Fluid Shunts+") OR (MH "Choledochostomy") OR (MH "Cholecystostomy") OR (MH "Pancreaticojejunostomy") OR (MH "Pericardial Window Techniques") OR (MH "Peritoneovenous Shunt") OR (MH "Portasystemic Shunt, Surgical") OR (MH "Vasovasostomy") OR (MH "Decompression, Surgical+") OR (MH "Laparotomy") OR (MH "Limb Salvage") OR (MH "Lymph Node Excision+") OR (MH "Mastectomy+") OR (MH "Metastasectomy") OR (MH "Microsurgery+") OR (MH "Minimally Invasive Procedures+") OR (MH "Myotomy+") OR (MH "Neurosurgery+") OR (MH "Orthopedic Surgery+") OR (MH "Ostomy+") OR (MH "Prostheses and Implants+") OR (MH "Surgery, Cardiovascular+") OR (MH "Surgery, Computer-Assisted+") OR (MH "Surgery, Elective+") OR (MH "Surgery, Endocrine+") OR (MH "Eye Surgery+") OR (MH "Surgery, Laparoscopic+") OR (MH "Surgery, Ob-Gyn+") OR (MH "Surgery, Oral+") OR (MH "Surgery, Otorhinolaryngologic+") OR (MH "Surgery, Plastic+") OR (MH "Surgery, Podiatric+") OR (MH "Surgery, Prophylactic+") OR (MH "Surgery, Reconstructive+") OR (MH "Surgery, Urogenital+") OR (MH "Thoracic Surgery+") OR (MH "Transplantation+") OR (MH "Appendectomy") OR (MH "Biliary Tract Surgical Procedures+") OR (MH "Surgery, Digestive System") OR (MH "Enterostomy+") OR (MH "Esophagoplasty") OR (MH "Fundoplication") OR (MH "Gastroenterostomy") OR (MH "Gastroplasty") OR (MH "Gastrostomy") OR (MH "Heller Myotomy") OR (MH "Hemorrhoidectomy") OR (MH "Hepatectomy") OR (MH "Liver Transplantation") OR (MH "Pancreas Transplantation+") OR (MH "Pancreatectomy") OR (MH "Pancreaticoduodenectomy") |
| S9 | S7 OR S8 |
| S8 | ((TI preoperat* OR AB preoperat* OR SU preoperat*) OR (TI pre-operat* OR AB pre-operat* OR SU pre-operat*) OR (TI presurg* OR AB presurg* OR SU presurg*) OR (TI pre-surg* OR AB pre-surg* OR SU pre-surg*) OR (TI preprocedur* OR AB preprocedur* OR SU preprocedur*) OR (TI pre-procedur* OR AB pre-procedur* OR SU pre-procedur*) OR (((TI before OR AB before OR SU before) OR (TI prior OR AB prior OR SU prior)) N5 ((TI surg* OR AB surg* OR SU surg*) OR (TI operat* OR AB operat* OR SU operat*) OR (TI procedur* OR AB procedur* OR SU procedur*))) OR (TI "prior to" OR AB "prior to" OR SU "prior to")) |
| S7 | (MH "Preoperative Care") OR (MH "Preoperative Education") OR (MH "Preoperative Period+") |
| S6 | S4 OR S5 |
| S5 | (((TI "low* fat" OR AB "low* fat" OR SU "low* fat") OR (TI "reduc* fat#" OR AB "reduc* fat#" OR SU "reduc* fat#") OR (TI "low* calorie*" OR AB "low* calorie*" OR SU "low* calorie*") OR (TI "reduc* calorie*" OR AB "reduc* calorie*" OR SU "reduc* calorie*") OR (TI "low* energy" OR AB "low* energy" OR SU "low* energy") OR (TI "reduc* energy" OR AB "reduc* energy" OR SU "reduc* energy") OR (TI hypocalor* OR AB hypocalor* OR SU hypocalor*) OR (TI "calori* restrict*" OR AB "calori* restrict*" OR SU "calori* restrict*") OR (TI "calorie control*" OR AB "calorie control*" OR SU "calorie control*") OR (TI "low* glyc*" OR AB "low* glyc*" OR SU "low* glyc*") OR (TI "reduc* glyc*" OR AB "reduc* glyc*" OR SU "reduc* glyc*") OR (TI "low* carbohydrate#" OR AB "low* carbohydrate#" OR SU "low* carbohydrate#") OR (TI "reduc* carbohydrate#" OR AB "reduc* carbohydrate#" OR SU "reduc* carbohydrate#") OR (TI "high* protein#" OR AB "high* protein#" OR SU "high* protein#") OR (TI "increas* protein#" OR AB "increas* protein#" OR SU "increas* protein#") OR (TI paleo* OR AB paleo* OR SU paleo*) OR (TI "intermittent fasting" OR AB "intermittent fasting" OR SU "intermittent fasting") OR (TI "meal replacement#" OR AB "meal replacement#" OR SU "meal replacement#") OR (TI "replacement meal#" OR AB "replacement meal#" OR SU "replacement meal#")) N2 ((TI diet# OR AB diet# OR SU diet#) OR (TI plan* OR AB plan* OR SU plan*) OR (TI regim* OR AB regim* OR SU regim*) OR (TI program* OR AB program* OR SU program*) OR (TI intervention# OR AB intervention# OR SU intervention#))) OR (((TI "weight loss" OR AB "weight loss" OR SU "weight loss") OR (TI "weight control" OR AB "weight control" OR SU "weight control") OR (TI "weight management" OR AB "weight management" OR SU "weight management") OR (TI "weight reduction" OR AB "weight reduction" OR SU "weight reduction")) N2 ((TI diet# OR AB diet# OR SU diet#) OR (TI plan* OR AB plan* OR SU plan*) OR (TI regim* OR AB regim* OR SU regim*) OR (TI program* OR AB program* OR SU program*) OR (TI intervention# OR AB intervention# OR SU intervention#))) OR (((TI antiobesity) OR (TI anti-obesity)) N3 ((TI medication#) OR (TI medicine#) OR (TI drug#) OR (TI agent#))) OR ((TI liraglutide OR AB liraglutide OR SU liraglutide) OR (TI saxenda OR AB saxenda OR SU saxenda) OR (TI victoza OR AB victoza OR SU victoza) OR (TI semaglutide OR AB semaglutide OR SU semaglutide) OR (TI ozempic OR AB ozempic OR SU ozempic) OR (TI wegovy OR AB wegovy OR SU wegovy) OR (TI tirzepatide OR AB tirzepatide OR SU tirzepatide) OR (TI exenatide OR AB exenatide OR SU exenatide) OR (TI bydureon OR AB bydureon OR SU bydureon) OR (TI byetta OR AB byetta OR SU byetta) OR (TI lixisenatide OR AB lixisenatide OR SU lixisenatide) OR (TI albiglutide OR AB albiglutide OR SU albiglutide) OR (TI tanzeum OR AB tanzeum OR SU tanzeum) OR (TI eperzan OR AB eperzan OR SU eperzan) OR (TI dulaglutide OR AB dulaglutide OR SU dulaglutide) OR (TI trulicity OR AB trulicity OR SU trulicity) OR (TI phentermine OR AB phentermine OR SU phentermine) OR (TI adipex-p OR AB adipex-p OR SU adipex-p) OR (TI suprenza OR AB suprenza OR SU suprenza) OR (TI topiramate OR AB topiramate OR SU topiramate) OR (TI topamax OR AB topamax OR SU topamax) OR (TI qsymia OR AB qsymia OR SU qsymia) OR (TI lorcaserin OR AB lorcaserin OR SU lorcaserin) OR (TI belviq OR AB belviq OR SU belviq) OR ((TI naltrexone OR AB naltrexone OR SU naltrexone) AND (TI bupropion OR AB bupropion OR SU bupropion)) OR (TI sibutramine OR AB sibutramine OR SU sibutramine) OR (TI rimonabant OR AB rimonabant OR SU rimonabant) OR (TI benzphetamine OR AB benzphetamine OR SU benzphetamine) OR (TI diethylpropion OR AB diethylpropion OR SU diethylpropion) OR (TI tenuate OR AB tenuate OR SU tenuate) OR (TI phendimetrazine OR AB phendimetrazine OR SU phendimetrazine) OR (TI bontril OR AB bontril OR SU bontril) OR (TI orlistat OR AB orlistat OR SU orlistat) OR (TI xenical OR AB xenical OR SU xenical)) |
| S4 | (MH "Diet Therapy") OR (MH "DASH Diet") OR (MH "Diet, Ketogenic") OR (MH "Diet, Nordic") OR (MH "Diet, Paleolithic") OR (MH "Diet, Reducing") OR (MH "Restricted Diet+") OR (MH "Obesity/DT/DH/TH") OR (MH "Weight Reduction Programs") OR (MH "Antiobesity Agents+") |
| S3 | S1 OR S2 |
| S2 | (((TI bodyweight OR AB bodyweight OR SU bodyweight) OR (TI weight OR AB weight OR SU weight)) N3 ((TI loss OR AB loss OR SU loss) OR (TI losing OR AB losing OR SU losing) OR (TI lose OR AB lose OR SU lose) OR (TI lost OR AB lost OR SU lost) OR (TI reduc* OR AB reduc* OR SU reduc*) OR (TI manage* OR AB manage* OR SU manage*) OR (TI control OR AB control OR SU control))) OR ((TI bmi) OR (TI "body mass index")) ,kf. OR (((AB bmi) OR (AB "body mass index")) N3 ((AB chang*) OR (AB reduc*) OR (AB low*) OR (AB decreas*))) OR ((TI waist OR AB waist OR SU waist) N2 ((TI circumference OR AB circumference OR SU circumference) OR (TI "hip ratio#" OR AB "hip ratio#" OR SU "hip ratio#"))) OR ((TI abdom* OR AB abdom* OR SU abdom*) W1 (TI circumference OR AB circumference OR SU circumference)) |
| S1 | (MH "Obesity") OR (MH "Obesity, Morbid") OR (MH "Body Weight Changes") OR (MH "Weight Loss") OR (MH "Body Mass Index") OR (MH "Waist Circumference") OR (MH "Waist-Hip Ratio") |

**Web of Science**

| #▲ | Searches |
| --- | --- |
| 1 | TS=((bodyweight OR weight) NEAR/3 (loss OR losing OR lose OR lost OR reduc* OR manage* OR control)) OR TS=(bmi OR "body mass index") OR TS=(waist NEAR/2 (circumference OR "hip ratio$")) OR TS=(abdom* NEAR/0 circumference) OR TS=(obes* OR overweight) Editions: WOS.SCI,WOS.ISTP |
| 2 | TS=(("low* fat" OR "reduc* fat$" OR "low* calorie*" OR "reduc* calorie*" OR "low* energy" OR "reduc* energy" OR hypocalor* OR "calori* restrict*" OR "calorie control*" OR "low* glyc*" OR "reduc* glyc*" OR "low* carbohydrate$" OR "reduc* carbohydrate$" OR "high* protein$" OR "increas* protein$" OR paleo* OR "intermittent fasting" OR "meal replacement$" OR "replacement meal$") NEAR/2 (diet$ OR plan* OR regim* OR program* OR intervention$)) OR TS=(("weight loss" OR "weight control" OR "weight management" OR "weight reduction") NEAR/2 (diet$ OR plan* OR regim* OR program* OR intervention$)) OR TI=((antiobesity OR anti-obesity) NEAR/3 (medication$ OR medicine$ OR drug$ OR agent$)) OR TS=(liraglutide OR saxenda OR victoza OR semaglutide OR ozempic OR wegovy OR tirzepatide OR exenatide OR bydureon OR byetta OR lixisenatide OR albiglutide OR tanzeum OR eperzan OR dulaglutide OR trulicity OR phentermine OR adipex-p OR suprenza OR topiramate OR topamax OR qsymia OR lorcaserin OR belviq OR (naltrexone AND bupropion) OR sibutramine OR rimonabant OR benzphetamine OR diethylpropion OR tenuate OR phendimetrazine OR bontril OR orlistat OR xenical) Editions: WOS.SCI,WOS.ISTP |
| 3 | TS=(preoperat* OR pre-operat* OR presurg* OR pre-surg* OR preprocedur* OR pre-procedur* OR ((before OR prior) NEAR/5 (surg* OR operat* OR procedur*)) OR "prior to") Editions: WOS.SCI,WOS.ISTP |
| 4 | TS=(surg* OR operat* OR procedure$) Editions: WOS.SCI,WOS.ISTP |
| 5 | #4 AND #3 AND #2 AND #1 Editions: WOS.SCI,WOS.ISTP |
| 6 | TS=((preoperat* OR pre-operat* OR presurg* OR pre-surg* OR preprocedur* OR pre-procedur*) NEAR/5 ("weight loss" OR "weight control" OR "weight management" OR "weight reduction")) Editions: WOS.SCI,WOS.ISTP |
| 7 | #6 OR #5 Editions: WOS.SCI,WOS.ISTP |
| 8 | (TS=(((cohort OR prospective OR retrospective OR "follow up" OR longitudinal) NEAR/0 stud*) OR "cohort analysis") OR TS=(random* or blind* or allocat* or assign* or trial* or placebo* or crossover* or cross-over*)) NOT TS=(animals or animal or mice or mus or mouse or murine or woodmouse or rats or rat or murinae or muridae or cottonrat or cottonrats or hamster or hamsters or cricetinae or rodentia or rodent or rodents or pigs or pig or swine or swines or piglets or piglet or boar or boars or sus scrofa or ferrets or ferret or polecat or polecats or mustela putorius or guinea pigs or guinea pig or cavia or callithrix or marmoset or marmosets or cebuella or hapale or octodon or chinchilla or chinchillas or gerbillinae or gerbil or gerbils or jird or jirds or merione or meriones or rabbits or rabbit or hares or hare or diptera or flies or fly or dipteral or drosphila or drosophilidae or cats or cat or carus or felis or nematoda or nematode or nematoda or nematode or nematodes or sipunculida or dogs or dog or canine or canines or canis or sheep or sheeps or mouflon or mouflons or ovis or goats or goat or capra or capras or rupicapra or chamois or haplorhini or monkey or monkeys or anthropoidea or anthropoids or saguinus or tamarin or tamarins or leontopithecus or hominidae or ape or apes or pan or paniscus or pan paniscus or bonobo or bonobos or troglodytes or pan troglodytes or gibbon or gibbons or siamang or siamangs or nomascus or symphalangus or chimpanzee or chimpanzees or prosimians or bush baby or prosimian or bush babies or galagos or galago or pongidae or gorilla or gorillas or pongo or pygmaeus or pongo pygmaeus or orangutans or pygmaeus or lemur or lemurs or lemuridae or horse or horses or pongo or equus or cow or calf or bull or chicken or chickens or gallus or quail or bird or birds or quails or poultry or poultries or fowl or fowls or reptile or reptilia or reptiles or snakes or snake or lizard or lizards or alligator or alligators or crocodile or crocodiles or turtle or turtles or amphibian or amphibians or amphibia or frog or frogs or bombina or salientia or toad or toads or epidalea calamita or salamander or salamanders or eel or eels or fish or fishes or pisces or catfish or catfishes or siluriformes or arius or heteropneustes or sheatfish or perch or perches or percidae or perca or trout or trouts or char or chars or salvelinus or fathead minnow or minnow or cyprinidae or carps or carp or zebrafish or zebrafishes or goldfish or goldfishes or guppy or guppies or chub or chubs or tinca or barbels or barbus or pimephales or promelas or poecilia reticulata or mullet or mullets or seahorse or seahorses or mugil curema or atlantic cod or shark or sharks or catshark or anguilla or salmonid or salmonids or whitefish or whitefishes or salmon or salmons or sole or solea or sea lamprey or lamprey or lampreys or pumpkinseed or sunfish or sunfishes or tilapia or tilapias or turbot or turbots or flatfish or flatfishes or sciuridae or squirrel or squirrels or chipmunk or chipmunks or suslik or susliks or vole or voles or lemming or lemmings or muskrat or muskrats or lemmus or otter or otters or marten or martens or martes or weasel or badger or badgers or ermine or mink or minks or sable or sables or gulo or gulos or wolverine or wolverines or minks or mustela or llama or llamas or alpaca or alpacas or camelid or camelids or guanaco or guanacos or chiroptera or chiropteras or bat or bats or fox or foxes or iguana or iguanas or xenopus laevis or parakeet or parakeets or parrot or parrots or donkey or donkeys or mule or mules or zebra or zebras or shrew or shrews or bison or bisons or buffalo or buffaloes or deer or deers or bear or bears or panda or pandas or wild hog or wild boar or fitchew or fitch or beaver or beavers or jerboa or jerboas or capybara or capybaras) Editions: WOS.SCI,WOS.ISTP |
| 9 | #8 AND #7 Editions: WOS.SCI,WOS.ISTP |
| 10 | TI=("obesity surg*" OR "metabolic surg*" OR "weight loss surg*" OR "gastric sleeve" OR "intragastric sleeve" OR "sleeve gastr*" OR "ileal sleeve" OR "gastric band*" OR "intragastric band*" OR "gastric bypass*" OR "intragastric bypass*" OR "jejuno* bypass*" OR "ileal bypass*" OR "gastric balloon*" OR "intragastric balloon*" OR roux-en-y OR "biliopancreatic diversion" OR "bilio-pancreatic diversion" OR "duodenal bypass*") OR TI=("bariatric surg*" NOT (nonbariatric OR non-bariatric)) Editions: WOS.SCI,WOS.ISTP |
| 11 | (#9) NOT #10 Editions: WOS.SCI,WOS.ISTP |

| Table S 1. Additional baseline characteristics of included studies | | | | | | | | | | |
| --- | --- | --- | --- | --- | --- | --- | --- | --- | --- | --- |
| **Date** | **Author year** | **Age** | **BMI** | **Female** | **Diabetes %** | **Hypertension %** | **Dyslipidaemia%** | **Obstructive sleep apnoea%** | **Active smoker%** | **Total N** |
| 2024 | Griffin et al, 2024a Australia | 48 ± 13 | 36 ± 5 | 44 | 8 | 31 | NR | 16 | 16 | 51 |
| 2024 | Griffin et al, 2024b Australia | 48 ± 13 | 42 ± 6 | 107 | 16 | 37 | NR | 19 | 13 | 141 |
| 2021 | Hamilton-Reeves et al, 2021USA | 60 ± 5 | 30 ± 3 | 0 | NR | NR | NR | NR | 10 | 20 |
| 2022 | Liang et al, 2018 USA | 50 ± 10 | 37 ± 3 | 83 | 31 | NR | NR | NR | 6 | 118 |
| 2012 | de Luis et al, 2012 Spain | 65 ± 9 | 39 ± 5 | 33 | NR | NR | NR | NR | NR | 40 |
| 2023 | Adrados et al, 2023 USA | NR | 42 ± 2 | NR | NR | NR | NR | NR | NR | 700 |
| 2015 | Rosen et al, 2015 USA | 54 ± 9 | 49 ± 10 | 20 | 28 | NR | NR | NR | NR | 25 |
| 2021 | Kashihara et al, 2021a Japan | 67 ± 12 | 27 ± 5 | 47 | NR | NR | NR | NR | NR | 120 |
| 2017 | Sun et al, 2017 USA | 60 ± 13 | 27 ± 5 | 4 | 15 | 47 | 47 | NR | 20 | 414 |
| 2016 | Burnand et al, 2014 UK | 46 ± 12 | 35 ± 3 | 42 | NR | NR | NR | NR | NR | 46 |
| 2020 | Wilson et al, 2020 Australia | 66 ± 7 | 29 ± 3 | 0 | NR | NR | NR | NR | NR | 43 |
| 2021 | Maruyama et al, 2021 Japan | 58 ± 16 | 32 ± 3 | NR | 20 | 60 | 40 | NR | NR | 5 |
| 2019 | Liljensoe et al, 2019 Denmark | 65 ± 9 | 31 ± 4 | 54 | 30 | 59 | NR | NR | 11 | 76 |
| 2021 | Aubrey et al, 2021 Canada | 58 ± 10 | 48 ± 7 | 49 | 51 | 59 | NR | NR | NR | 49 |
| 2023 | Saito et al, 2023 Japan | 73 ± 8 | 28 ± 3 | 8 | NR | NR | NR | NR | NR | 32 |
| 2024 | Rechenmacher et al, 2024 USA | 65 ± 8 | 39 ± 5 | 68 | NR | NR | NR | NR | NR | 90 |
| 2021 | Kashihara et al, 2021b Japan | 67 ± 7 | 30 ± 5 | 6 | NR | NR | NR | NR | NR | 22 |
| 2020 | Lingamfelter et al, 2020 USA | 63 ± 9 | 43 ± 2 | 80 | NR | NR | NR | NR | NR | 133 |
| 2024 | Turcotte et al, 2024 USA | 66 ± 9 | 32 ± 4 | 104 | 10 | 59 | 51 | NR | 5 | 134 |
| 2019 | Barth et al, 2019 USA | 56 ± 12 | 33 ± 7 | 27 | 17 | NR | NR | NR | NR | 60 |
| 2020 | Hollis et al, 2020 Australia | 50 ± 13 | 41 ± 6 | 29 | NR | NR | NR | NR | NR | 46 |
| 2024 | Yoshiya 2024 Japan - donor weight reduction right lobe graft | 46 ± 11 | 27 ± 4 | 10 | NR | NR | NR | NR | NR | 25 |
| 2024 | Yoshiya 2024 Japan - recipient weight reduction group | 57 ± 9 | 30 ± 4 | 10 | NR | NR | NR | NR | NR | 22 |
| 2024 | Yoshiya 2024 Japan - donor weight reduction left lobe graft | 39 ± 5 | 27 ± 4 | 2 | NR | NR | NR | NR | NR | 16 |
| 2020 | Ssentongo et al, 2020 USA | 54 ± 8 | 36 ± 6 | 132 | 27 | 64 | 34 | 17 | 11 | 230 |
| 2022 | Maskal et al, 2022 USA | 56 ± 15 | 46 ± 9 | 125 | 41 | 62 | 35 | NR | 11 | 191 |
| 2019 | Inoue et al, 2019 Japan | 71 ± 10 | 26 ± 2 | 7 | 15 | 73 | 18 | NR | NR | 33 |
| 2021 | Imai et al, 2021 Japan | 50 ± 7 | 35 ± 4 | 16 | 63 | NR | NR | NR | NR | 16 |
| 2016 | Doyle et al, 2016 Canada | 41 ± 12 | 33 ± 4 | 10 | NR | NR | NR | NR | NR | 69 |
| 1997 | Pekkarinen et al, 1997 Finland | 50 ± 10 | 44 ± 2 | 18 | NR | NR | NR | NR | NR | 30 |
| 2024 | McKechnie et al, 2024 Canada | 67 ± 11 | 34± 4 | 88 | NR | NR | NR | NR | 15 | 190 |
| 2025 | Koutoukidis et al, 2025 UK | 64 ± 9 | 35 ± 5 | 28 | 20 | 46 | NR | NR | NR | 71 |
| 2025 | Morgan et al, 2025 USA | 60 ± 7 | 31 ± 4 | 0 | 24 | NR | NR | NR | 8 | 29 |
| 2025 | Spurzem et al 2025a USA | 59 ± 12 | 38 ± 4 | 29 | NR | NR | NR | NR | NR | 46 |
| 2025 | Kim et al, 2025 USA | 62 ± 8 | ≥ 40 | 3975 | 53 | NR | NR | NR | 48 | 5950 |
| 2025 | Spurzem et al, 2025b USA | 58 ± 11 | 39 ± 6 | 46 | NR | NR | NR | NR | NR | 70 |
| 2025 | Ayres et al, 2025 Australia | 56 ± 6 | 45 ± 7 | 25 | NR | NR | NR | NR | NR | 25 |
| Mean (SD) or N (%) | | 58 (8) | 35.6 (6.4) | 5301 (61%) | 3513 (46%) | 741 (53%) | 415 (41%) | 75 (18%) | 3046 (41%) | 9378 |
| Data are reported as mean ± SD or proportion of total N. Age in years and BMI in kg/m^2^. NR: Not reported. | | | | | | | | | | |

| Table S 2. Characteristics of the interventions of the included studies | | | | | | | | | |
| --- | --- | --- | --- | --- | --- | --- | --- | --- | --- |
| **Date** | **Author year** | **Intervention** | **Provider** | **Delivery and  Intensity** | **Energy intake prescription** | **Duration** | **Comparison** | **Commercial formula** | **Rich in protein** |
| 2024 | Griffin et al, 2024a Australia | Commercial VLCD meal replacement, physical activity was encouraged | Dietitian | TDR | 800kcal/day, VLCD | 2-12 weeks | standard care | Optifast or Optislim | Additional protein-rich food(s) |
| 2024 | Griffin et al, 2024b Australia | VLCD meal replacement products | Dietitian | TDR | Phase 1-3: 800-1,200 kcal/day, VLCD | 13 weeks (median) | NA | Optifast or Optislim | Additional protein-rich foods to meet individualized protein requirements (0.8–1 g/kg of adjusted ideal body weight per day). |
| 2021 | Hamilton-Reeves et al, 2021USA | Lifestyle Coaching+ self-selected foods along with meal replacements | Staff in the clinical and translational science unit | PDR | 1000 -1200kcal/day, LCD | 8.3 (3.5 -16) weeks | standard care | Medifast 5&2&2 | NR |
| 2022 | Liang et al, 2018 USA | Nutritional counselling. Meetings and exercise programs (rehabilitation): received instructions and a DVD with a number of different exercises | Multidisciplinary team of surgical specialists | 4-part education meetings | Targeted ~1 kg per week weight loss | Up to 6 months | standard counselling | NA | NR |
| 2012 | de Luis et al, 2012 Spain | Hypocaloric commercial formula | Staff in the clinical nutrition unit | PDR | 1109.3 kcal/day, LCD | 130.2 ± 80.8 days | no commercial diet replacement | Optisource | 63g |
| 2023 | Adrados et al, 2023 USA | Weight loss consultation | Surgeons | Unclear | BMI <40 before performing TKA | Within 1 year prior to surgery | NA | NA |  |
| 2015 | Rosen et al, 2015 USA | A protein sparing modified fast diet | Medical weight loss specialist | Unclear | <800 kcal/day | 17 (6-36) months | NA | NA | The PSMF is based on a diet of 1.2–1.4 g/kg/day of protein |
| 2021 | Kashihara et al, 2021 Japan | Preoperative weight loss program (PWLP): caloric restriction + exercise | Staff in the department of rehabilitation | Unclear | 1200 kcal/day | 29.6 (15–­70) days | No PWLP | NA | NR |
| 2017 | Sun et al, 2017 USA | VA-sponsored medical weight loss program, outside weight loss programs, self-guided weight loss | Veterans Affairs Medical Center | Unclear | NR | 226.1 ± 145.9 days | Nontrial | NA | NR |
| 2016 | Burnand et al, 2014 UK | The VLCD is a previously well used diet based on commercially available low-calorie shakes. | Dietician | Unclear | 800 kcal/day | 2 weeks | A diet sheet to follow | Slimfast | NR |
| 2020 | Wilson et al, 2020 Australia | Personalized exercise plans. Three very low-calorie diet meal replacement products consisting of shakes, bars, or soups. | Supervised by certified allied health professionals | TDR | 803 -1004 kcal/day | 29 [24–35] days | NA | KicStart; Optifast; Dr MaCleod’s | Optimisation of daily protein (1.0–1.07 g/kg body weight/d according to age related recommendations) |
| 2021 | Maruyama et al, 2021 Japan | Commercial formula | Nutritionist | PDR | 770-940 kcal/day | 29 ± 9.46 days | NA | FD (Obe Cure®, USCure) | Rich in protein |
| 2019 | Liljensoe et al, 2019 Denmark | LCD commercially available formula foods | Dietician | TDR | 810 kcal/day | 8 weeks | standard care | Cambridge Weight Plan® | NR |
| 2021 | Aubrey et al, 2021 Canada | LCD + anti-obesity medication (AOM) | Obesity Medicine specialist | TDR/PDR | 1000-1200 kcal/d | 12 weeks to 6 months | NA | Optifast 900 | NR |
| 2023 | Saito et al, 2023 Japan | Preoperative weight loss program (WLP): diet + exercise | Doctors, nutritionists and physical therapists | Unclear | 1000 kcal/day | 21 ± 12 days | No intervention | NA | The protein mass was calculated as SBW x 1.2 g/day, including branched chain amino acid to maintain skeletal muscle mass. |
| 2024 | Rechenmacher et al, 2024 USA | The intervention included dietary plans (low-calorie, low-carbohydrate, very-low-calorie meal replacement), along with medication optimization, behavioural therapy, and exercise plans. | Staff in the institution’s weight loss clinic | Unclear | NR | 18 months prior to TKA | NA | NA | NR |
| 2021 | Kashihara et al, 2021 Japan | Pre-operative weight loss program (PWLP): diet and exercise | Department of Rehabilitation | Unclear | 1200 kcal/day | 26.3 ± 6.6 days | No intervention | NA | NR |
| 2020 | Lingamfelter et al, 2020 USA | Provided individualized meal plans and recommendations for diet changes | Registered dietician | Unclear | NR | 129±116 days | NA | NA | NR |
| 2024 | Turcotte et al, 2024 USA | A standardized Preoperative Weight Management Protocol. All patients received  written education materials consisting of detailed instructions on a hypocaloric diet, | Multidisciplinary team of surgeons and dieticians | TDR | 1200 kcal/day | 2 weeks | NA | NA | NR |
| 2019 | Barth et al, 2019 USA | 5 units of commercial formula plus an unlimited volume of calorie-free fluids per day | Dietitian | TDR | 800 kcal/day | 1 week | Standard care | Optifast 800 | 70g |
| 2020 | Hollis et al, 2020 Australia | Commercial VLCD | Research dietitian | TDR | 700 to 800 kcal/day | 8 weeks | standard care | Optifast | Intake with >0.75 g/kg adjusted body weight protein. |
| 2024 | Yoshiya 2024 Japan | Donors: LCD + exercise Recipients: exercise | Rehabilitation department and nutrition department | Unclear | 1000 kcal/day | Recipients: 2.2 ± 1.4 months Donors: 3.2 ± 5.8 months | NA | NA | NR |
| 2020 | Ssentongo et al, 2020 USA | Provided with the message that diet and exercise was important in preparation for operation | NR | Unclear | NR | 95 days (median) | NA | NA | NR |
| 2022 | Maskal et al, 2022 USA | Online coaching | Surgeon and health navigator | Unclear | NR | 4.5 (1.3-8.3) month | No intervention | NA | NR |
| 2019 | Inoue et al, 2019 Japan | Patients replaced one of three daily meals with commercial VLCD | Dietitian | PDR | NR | 20 days | NA | ObeCure® | NR |
| 2021 | Imai et al, 2021 Japan | Diet + exercise | Dietitian physiotherapist | PDR | 1200 kcal/day | 39.1±11.4 days (4 weeks) | NA | NA | 70 g proteins |
| 2016 | Doyle et al, 2016 Canada | Four Optifast shakes (225kCal/ per serving) were consumed per day in place of meals. | Dietitian | TDR | 900 kcal/day | 7.3 (4-15) weeks | NA | Optifast900 | 90 g proteins |
| 1997 | Pekkarinen et al, 1997 Finland | The VLCD preparation used was Modifast. The daily dose was three sachets | Unclear | TDR | 458 kcal/day | 14.4 (7-24) weeks | NA | Modifast | 52 g of protein |
| 2024 | McKechnie et al, 2024 Canada | Optifast 900 liquid formulation program for 2–4 weeks, two cups of low-kcal vegetables per day | Unclear | TDR | 900 kcal daily, VLED | 3.4 ± 1.4 weeks | No intervention | Optifast 900 | NR |
| 2025 | Koutoukidis et al, 2025 UK | LCD (TDR) vs. usual care | dietitian | TDR | 800 kcal/day | 33 (25-43) days | Usual care: local standard care pathway | Habitual Health, Ltd. | 76g protein/day |
| 2025 | Morgan et al, 2025 USA | calorie-restricted diet and exercise intervention | NR | Unclear | aiming for ~1 kg of weight loss/week between diagnosis and surgery | NR | NA | NA | NR |
| 2025 | Spurzem et al 2025a USA | Patients were prescribed GLP-1 agonists specifically for weight loss in addition to lifestyle changes | A multidisciplinary weight loss team comprised of surgeons, obesity medicine physicians, and registered dieticians | GLP-1 agonists administered continuously during the full preoperative period | asked to achieve a BMI of 33 kg/m2 or less prior to hernia repair. | 6.3 ± 4.0 months | without GLP-1 agonists | NR | NR |
| 2025 | Kim et al, 2025 USA | GLP-1 | NR (prescription medication; provider type not specified) | GLP-1 prescribed and filled preoperatively（individual drug formulation not detailed） | NR | at least 3 months before and after the date of the TKA. | without GLP-1 RA Use | NR | NA |
| 2025 | Spurzem et al, 2025b USA | lifestyle changes + GLP-1 agonists | A multidisciplinary weight loss team | GLP-1 agonists used continuously preoperatively alongside lifestyle modification; dose/frequency not reported | NR | Mean 8.2 ± 4.9 months from GLP-1 initiation to surgery | NR | NR | NR |
| 2025 | Ayres et al, 2025 Australia | VLCD | Dietitian-led program with involvement of gynaecologic oncologists, study nurses, and investigators | TDR | 800 kcal/day | 4–6 weeks (protocol-defined) | NA | Optifast | 0.8 g/kg adjusted ideal body weight |
| Total diet replacement (TDR)， Partial diet replacement (PDR) | | | | | | | | | |

| Table S 3. Surgical characteristics of included studies | | | | | | | | | | | |
| --- | --- | --- | --- | --- | --- | --- | --- | --- | --- | --- | --- |
| **Date** | **Author year** | **Hernia repair** | **Gynae**  **cology** | **Orthopaedic Surgery** | **Gastro**  **esophageal Surgery** | **Colorectal surgery** | **Cholecys**  **tectomy** | **Hepa**  **tectomy** | **Prosta**  **tectomy** | **Other** | **Cancer** |
| 2024 | Griffin et al, 2024a Australia | 8 | 25 | - | - | - | 18 | - | - | - | Mix |
| 2024 | Griffin et al, 2024b Australia | 12 | 54 | 7 | 16 | 19 | 31 | - | - | 2 | Mix |
| 2021 | Hamilton-Reeves et al, 2017 USA | - | - | - | - | - | - | - | 17 | - | Cancer patients |
| 2022 | Liang et al, 2018 USA | 93 | - | - | - | - | - | - | - | - | Non-cancer patients |
| 2012 | de Luis et al, 2012 Spain | - | - | 20 | - | - | - | - | - | - | Non-cancer patients |
| 2023 | Adrados et al, 2023 USA | - | - | 700 | - | - | - | - | - | - | Non-cancer patients |
| 2015 | Rosen et al, 2015 USA | 25 | - | - | - | - | - | - | - | - | Non-cancer patients |
| 2021 | Kashihara et al, 2021a Japan | - | - | - | - | 82 | - | - | - | - | Cancer patients |
| 2017 | Sun et al, 2017 USA | 414 | - | - | - | - | - | - | - | - | Non-cancer patients |
| 2016 | Burnand et al, 2014 UK | - | - | - | - | - | 46 | - | - | - | Non-cancer patients |
| 2020 | Wilson et al, 2020 Australia | - | - | - | - | - | - | - | 43 | - | Cancer patients |
| 2021 | Maruyama et al, 2021 Japan | - | - | - | 5 | - | - | - | - | - | Cancer patients |
| 2019 | Liljensoe et al, 2019 Denmark | - | - | 76 | - | - | - | - | - | - | Non-cancer patients |
| 2021 | Aubrey et al, 2021 Canada | - | 43 | - | - | - | - | - | - | - | Cancer patients |
| 2023 | Saito et al, 2023 Japan | - | - | - | - | - | - | 32 | - | - | Cancer patients |
| 2024 | Rechenmacher et al, 2024 USA | - | - | 90 | - | - | - | - | - | - | Non-cancer patients |
| 2021 | Kashihara et al, 2021b Japan | - | - | - | 22 | - | - | - | - | - | Cancer patients |
| 2020 | Lingamfelter et al, 2020 USA | - | - | 71 | - | - | - | - | - | - | Non-cancer patients |
| 2024 | Turcotte et al, 2024 USA | 134 | - | - | - | - | - | - | - | - | Non-cancer patients |
| 2019 | Barth et al, 2019 USA | - | - | - | - | - | - | 60 | - | - | Mix |
| 2020 | Hollis et al, 2020 Australia | 19 | - | - | - | - | 27 | - | - | - | Non-cancer patients |
| 2024 | Yoshiya et al, 2024 Japan | - | - | - | - | - | - | 63 | - | - | Mix |
| 2020 | Ssentongo et al, 2020 USA | 230 | - | - | - | - | - | - | - | - | Non-cancer patients |
| 2022 | Maskal et al, 2022 USA | 17 | - | - | - | - | - | - | - | - | Non-cancer patients |
| 2019 | Inoue et al, 2019 Japan | - | - | - | 33 | - | - | - | - | - | Cancer patients |
| 2021 | Imai et al, 2021 Japan | - | 10 | - | - | - | - | - | - | - | Cancer patients |
| 2016 | Doyle et al, 2016 Canada | - | - | - | - | - | - | 16 | - | - | Mix |
| 1997 | Pekkarinen et al, 1997 Finland | 6 | 5 | 6 | 4 | - | 1 | - | - | 4 | Mix |
| 2024 | McKechnie et al, 2024 Canada | - | - | - | - | 190 | - | - | - | - | Mix |
| 2025 | Koutoukidis et al, 2025 UK | - | - | - | - | 71 | - | - | - | - | Cancer patients |
| 2025 | Morgan et al, 2025 USA | - | - | - | - | - | - | - | 29 | - | Cancer patients |
| 2025 | Spurzem et al 2025a USA | 46 | - | - | - | - | - | - | - | - | Non-cancer patients |
| 2025 | Kim et al, 2025 USA | - | - | 5950 | - | - | - | - | - | - | Non-cancer patients |
| 2025 | Spurzem et al, 2025b USA | 70 | - | - | - | - | - | - | - | - | Non-cancer patients |
| 2025 | Ayres et al, 2025 Australia | - | 25 | - | - | - | - | - | - | - | Cancer patients |
| **Total** | | 1074 | 162 | 6920 | 80 | 362 | 123 | 171 | 89 | 6 | - |
| If a study did not specify cancer status but mentioned that the surgeries included cancer cases, it was categorized as 'Mix' in the cancer classification. | | | | | | | | | | | |

| 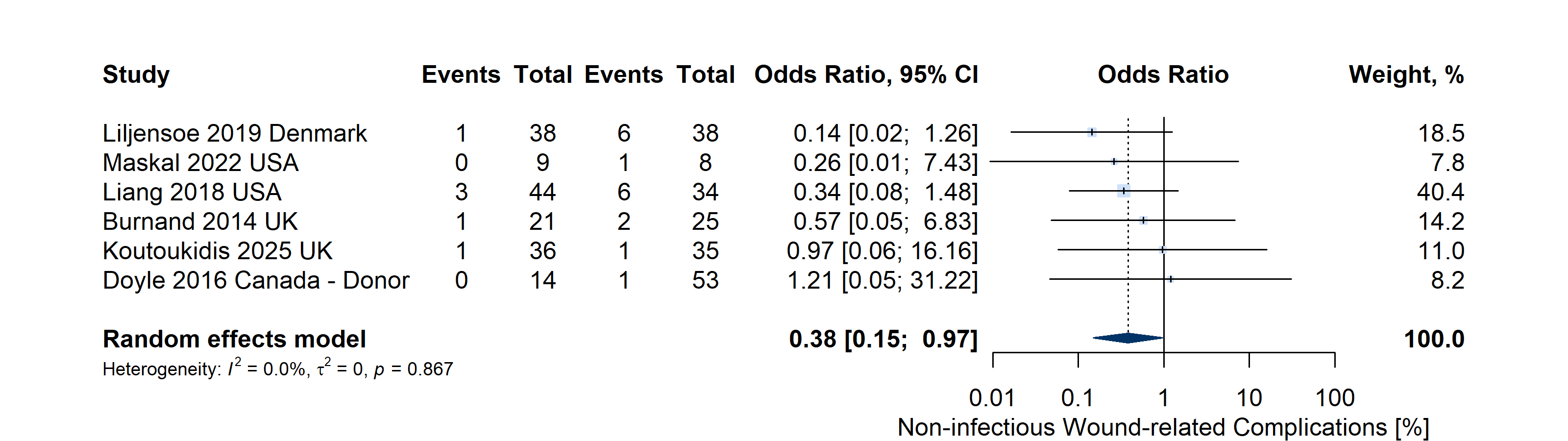 |
| --- |
| Figure S 1. Association between weight loss interventions on non-infectious wound-related complications  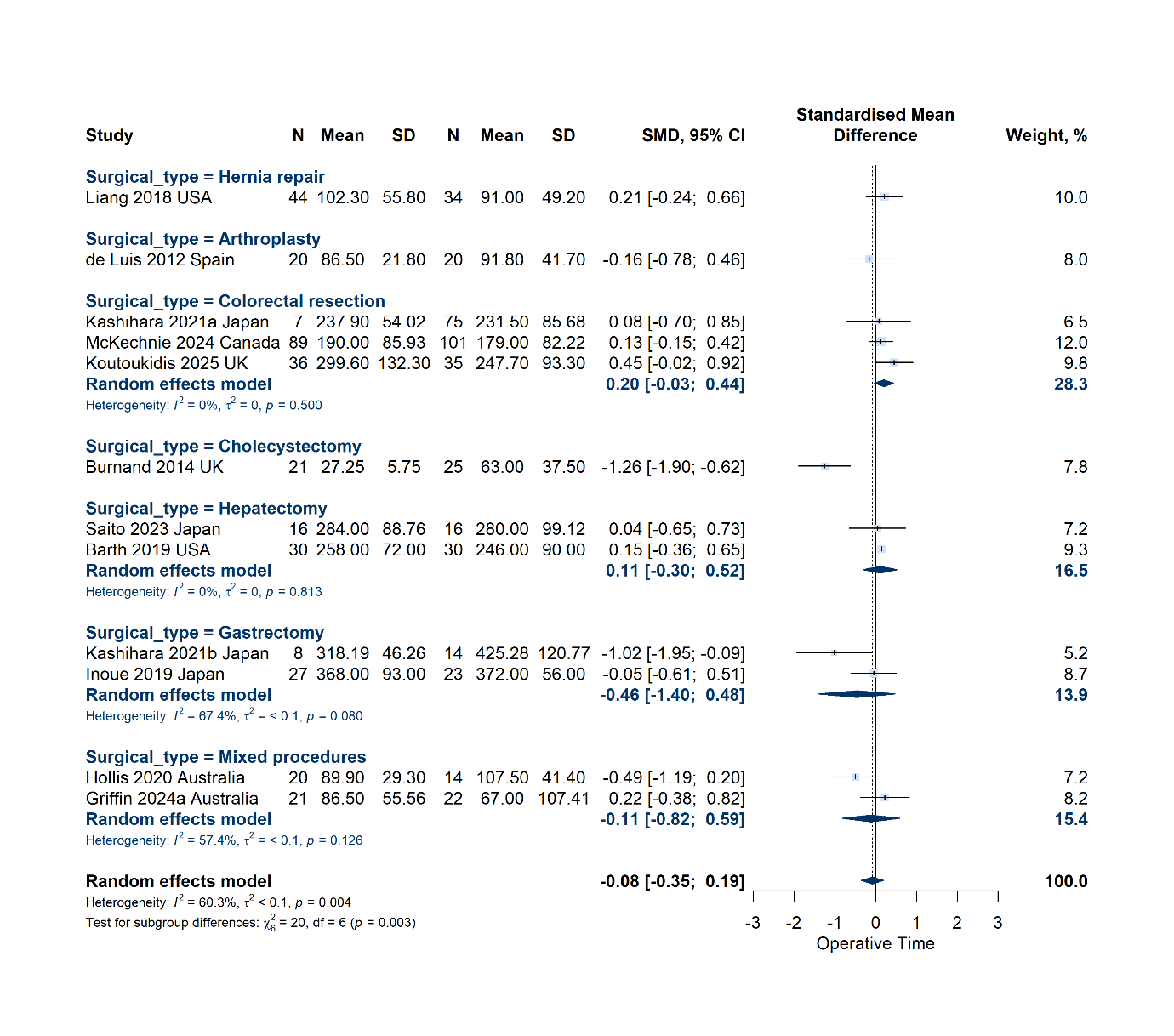  Figure S 2. Association between weight loss interventions and operative time  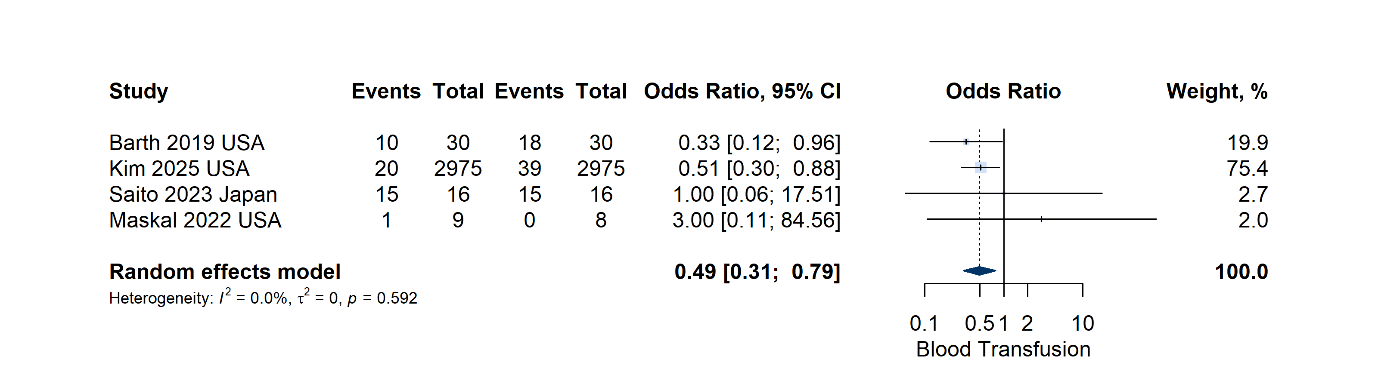 |

Figure S 3. Association between weight loss interventions and blood transfusion risk

| 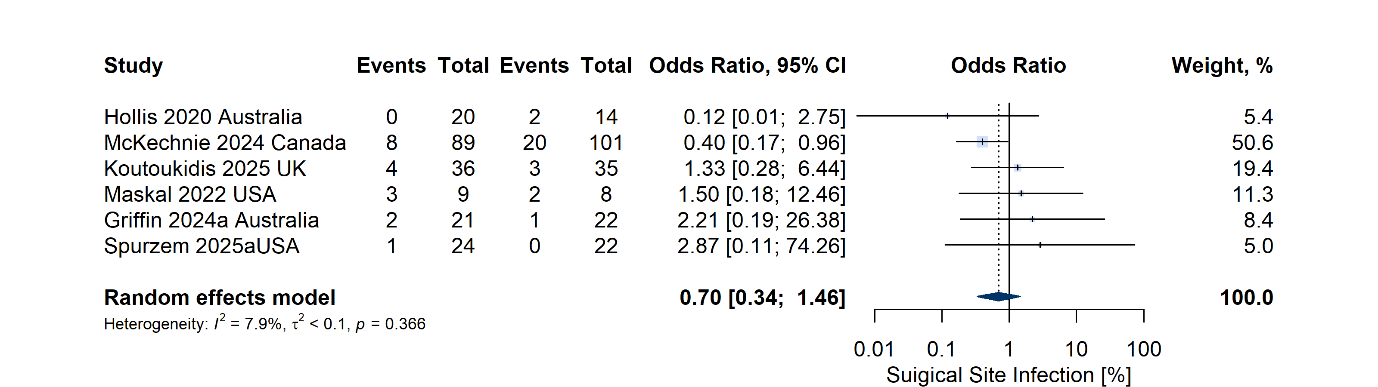 |
| --- |
| Figure S 4. Association between weight loss interventions and surgical site infection |

| **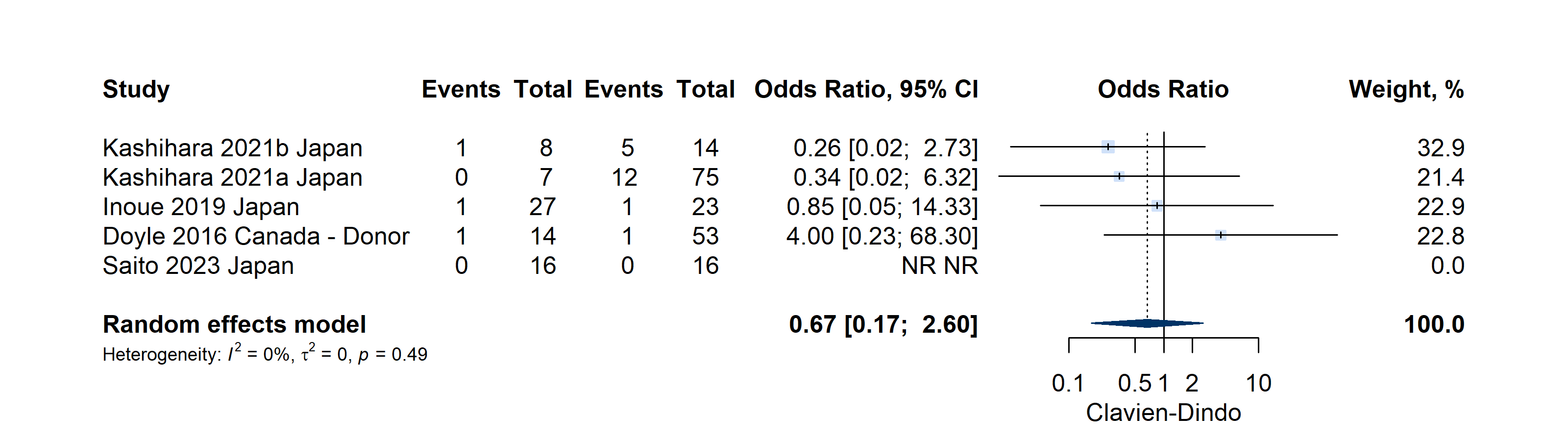** |
| --- |
| Figure S 5. Association between weight loss interventions and Clavien-Dindo≥3 |

| 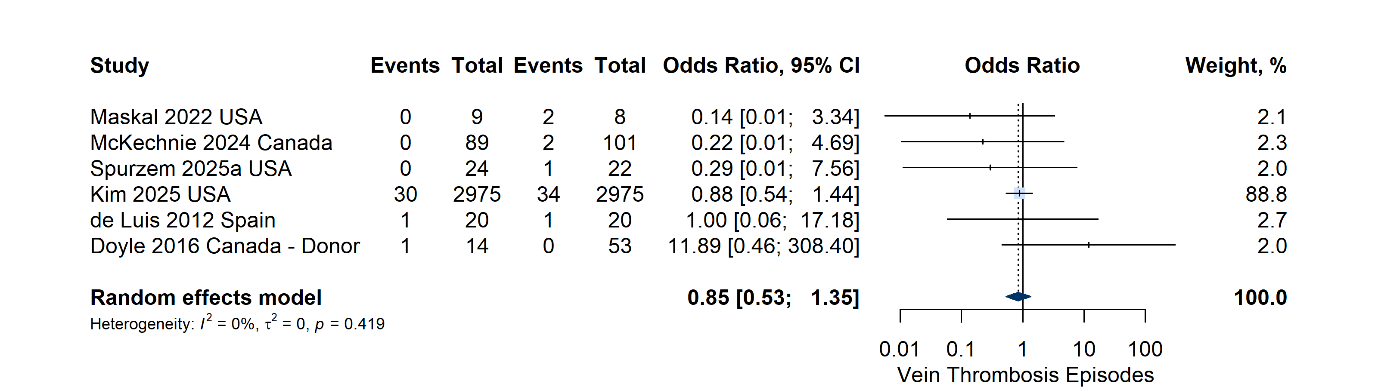 |
| --- |
| Figure S 6. Association between weight loss interventions and vein thrombosis episodes |

| 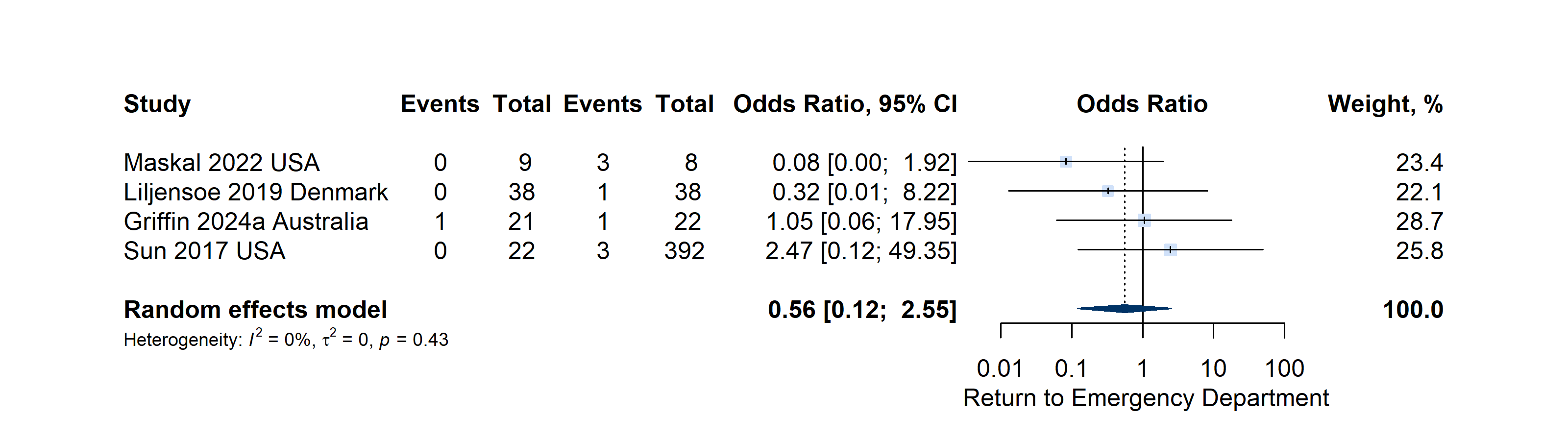 |
| --- |
| Figure S 7. Association between weight loss interventions and emergency department |

| 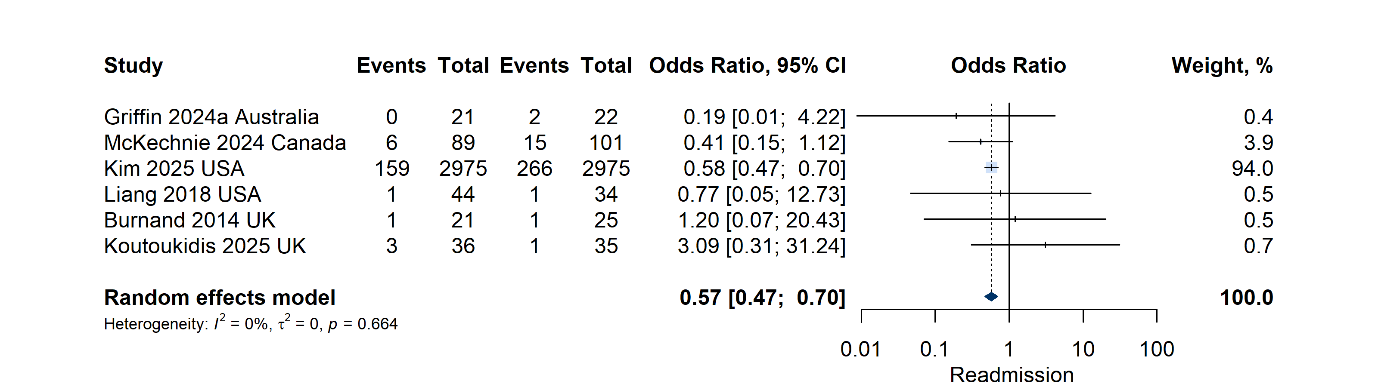 |
| --- |
| Figure S 8. Association between weight loss interventions and hospital readmission risk |
| 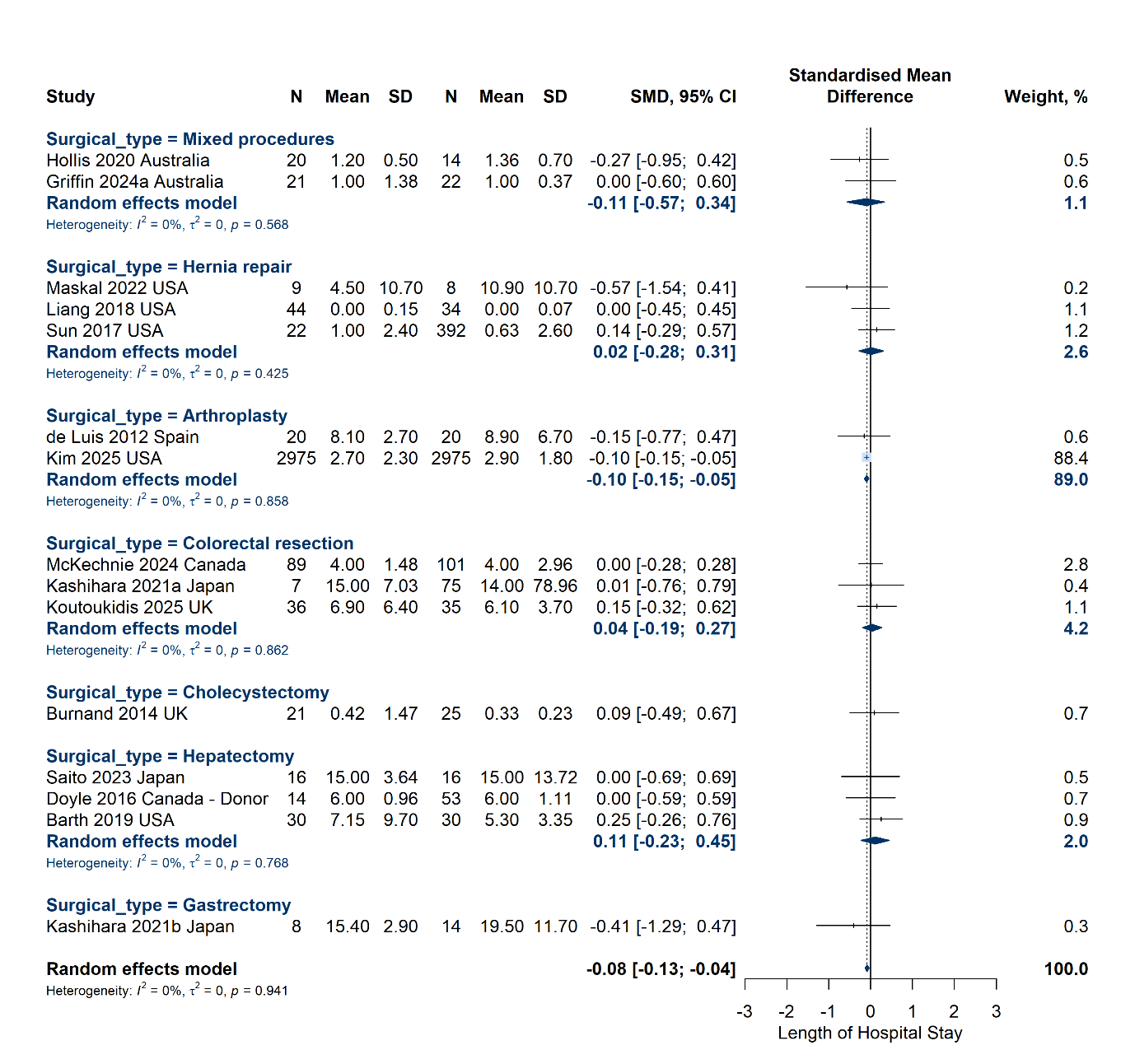 |
| Figure S 9. Association between weight loss interventions and length of hospital stay |


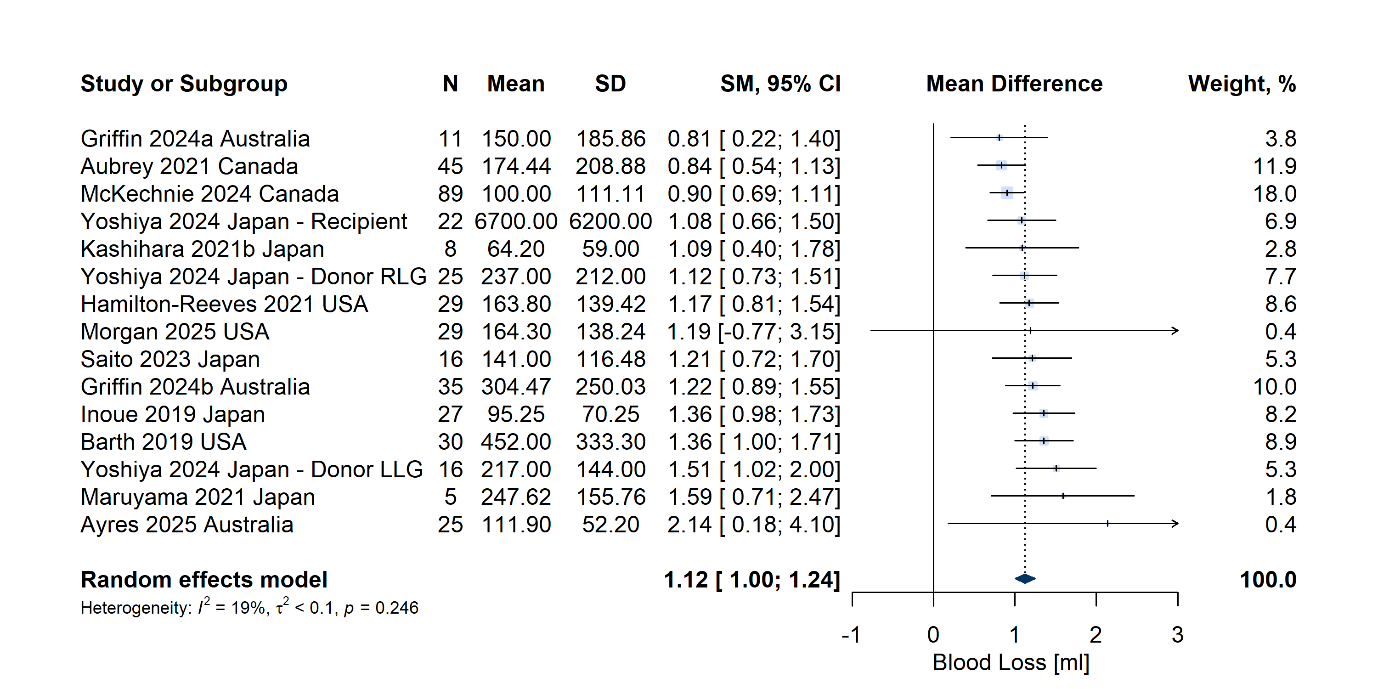


| Figure S 10. Pooled blood loss in single-arm studies  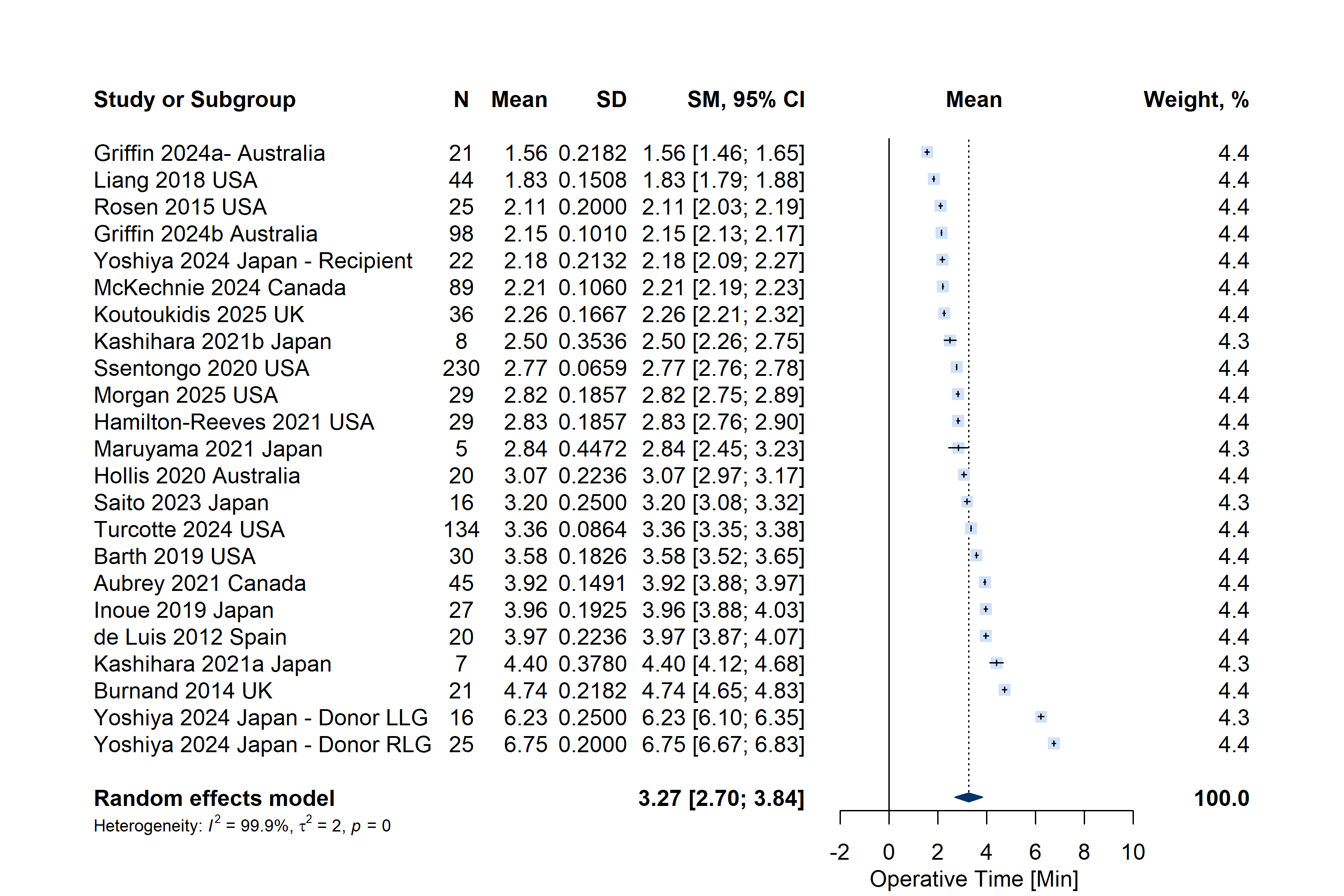  Figure S 11. Pooled operative time in single-arm studies |
| --- |
|  |
| 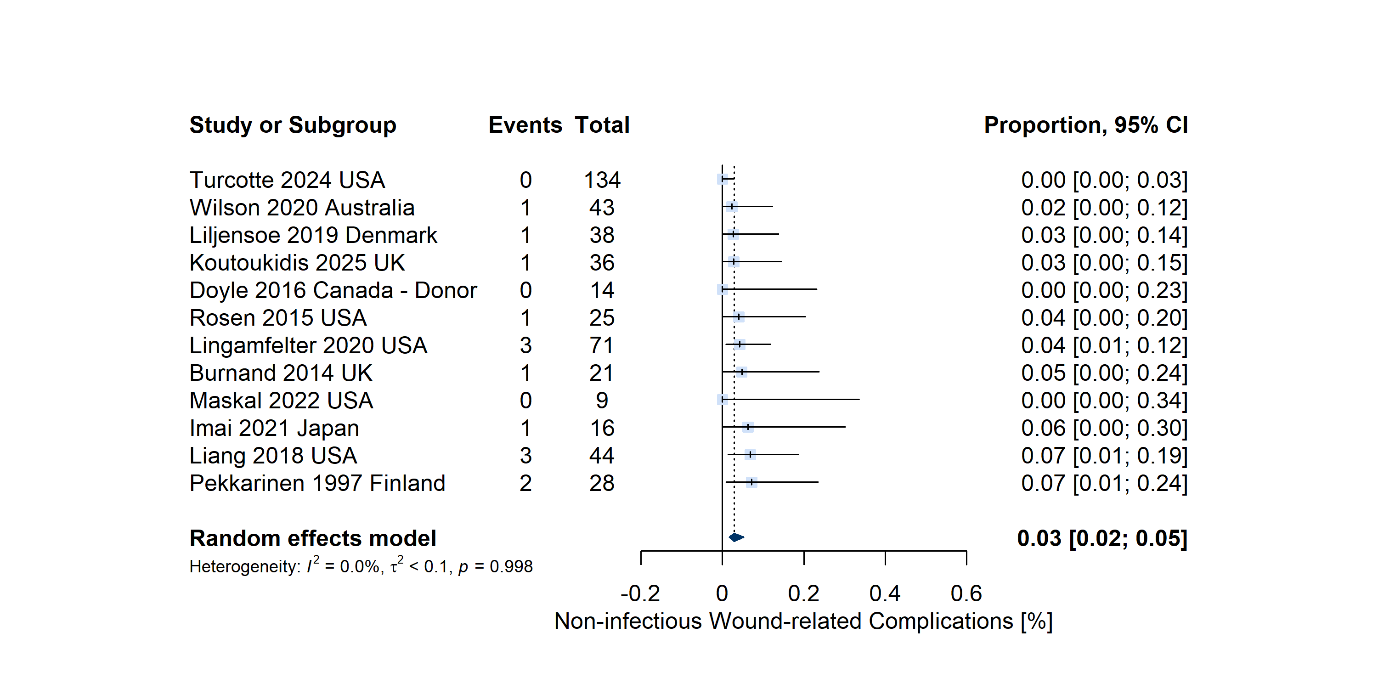 |
| Figure S 12. Pooled non-infectious wound-related complications rate in single-arm studies |


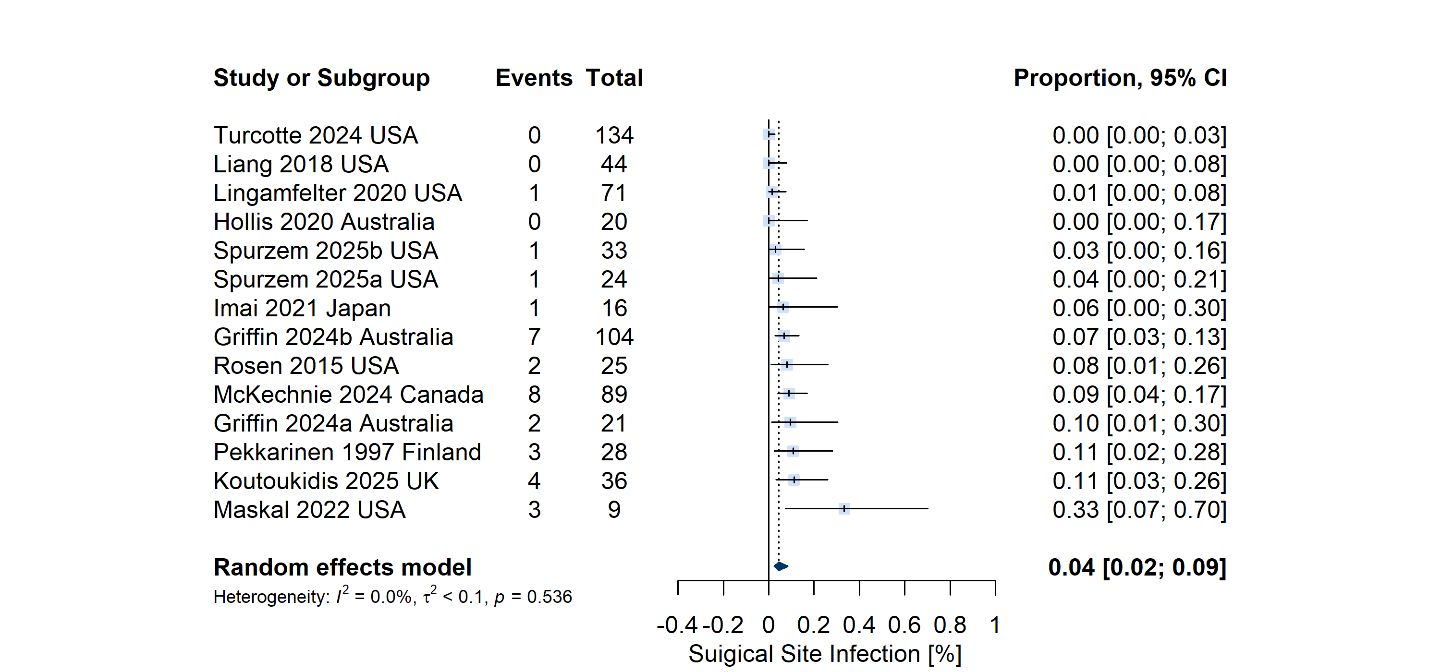


Figure S 13. Pooled surgical site infection rate in single-arm studies

| 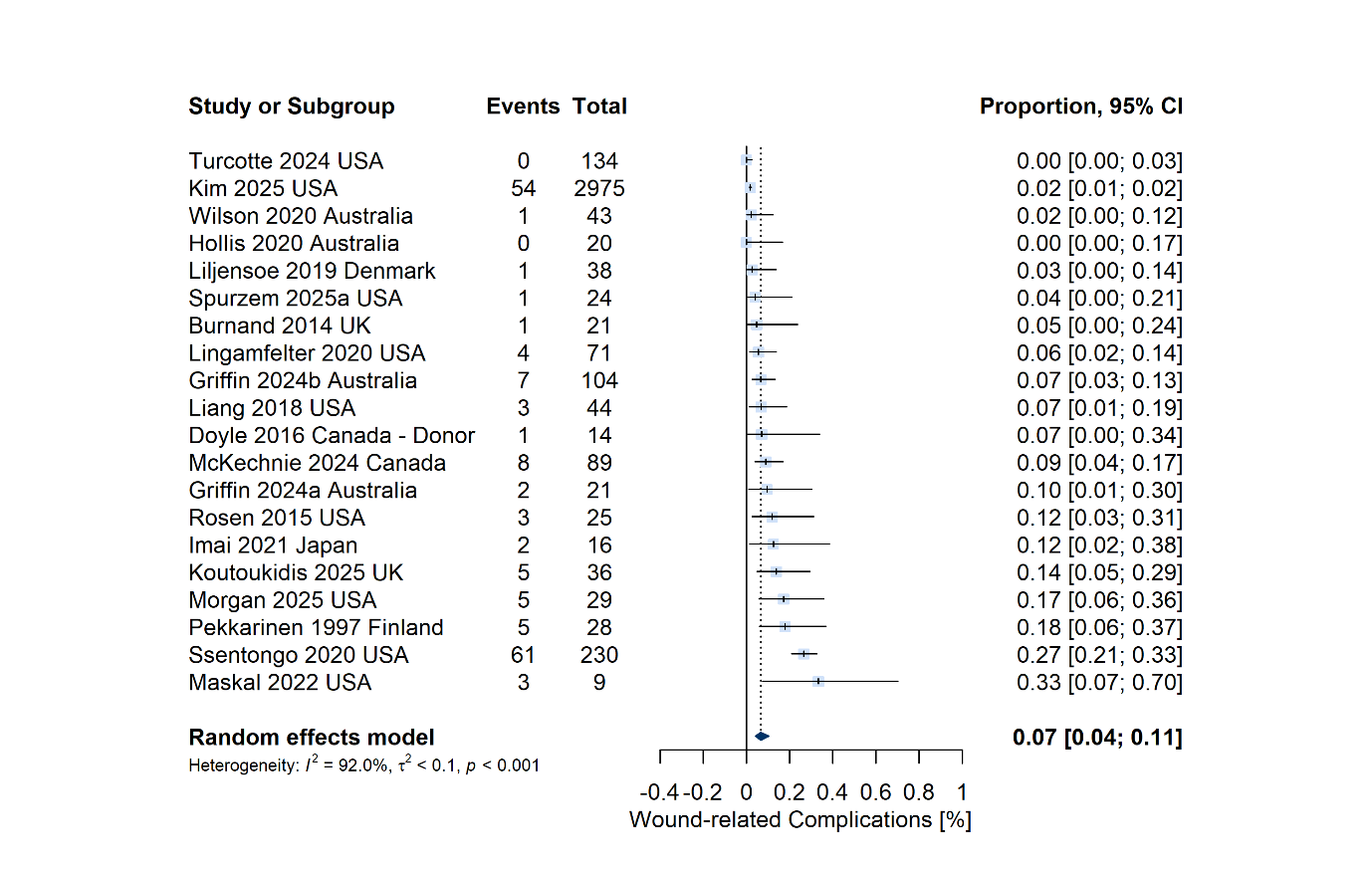 |
| --- |
| Figure S 14. Pooled wound-related complications rate in single-arm studies |
|  |
|  |

| 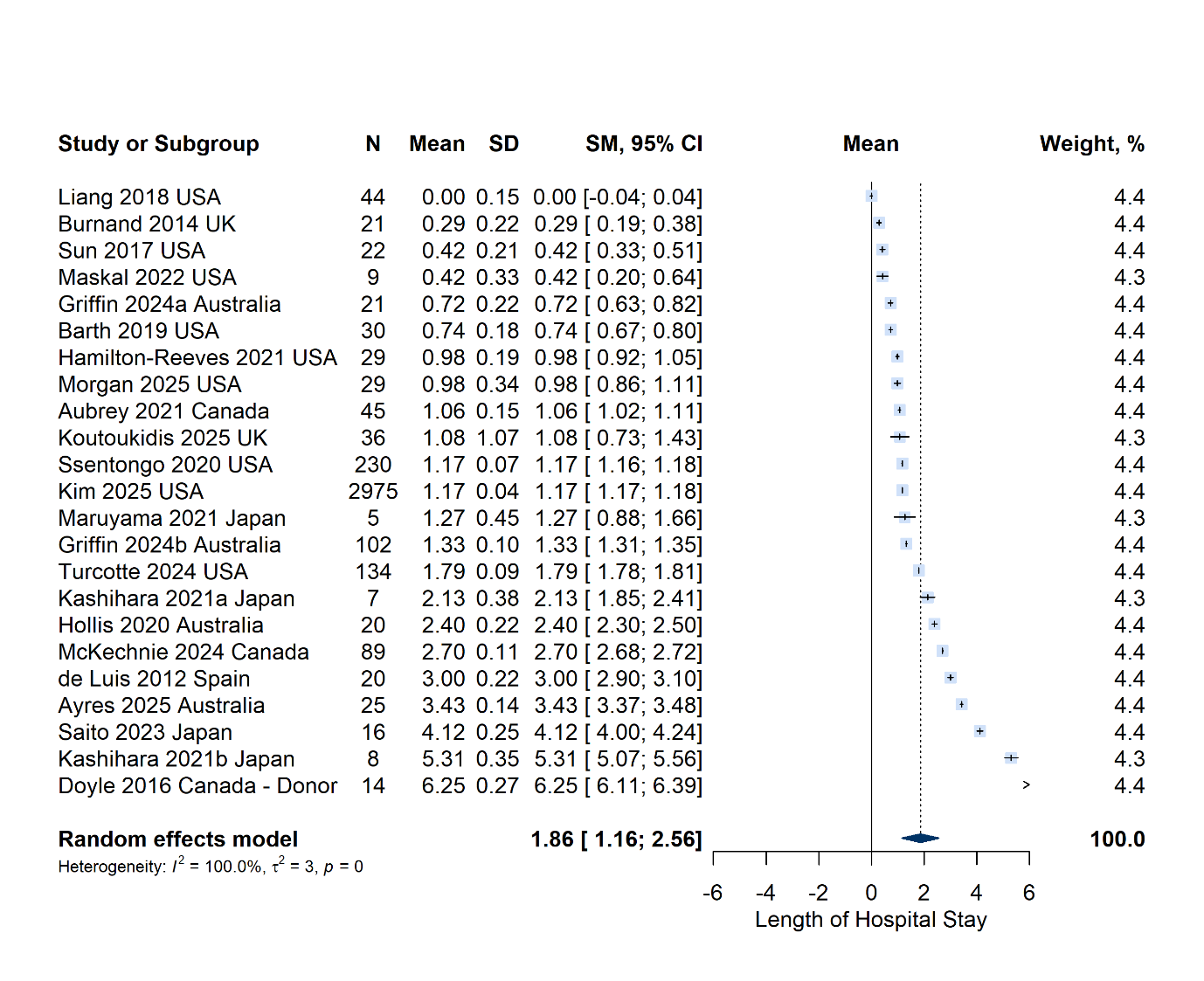 |
| --- |
| Figure S 15. Pooled length of hospital stay in single-arm studies |

| 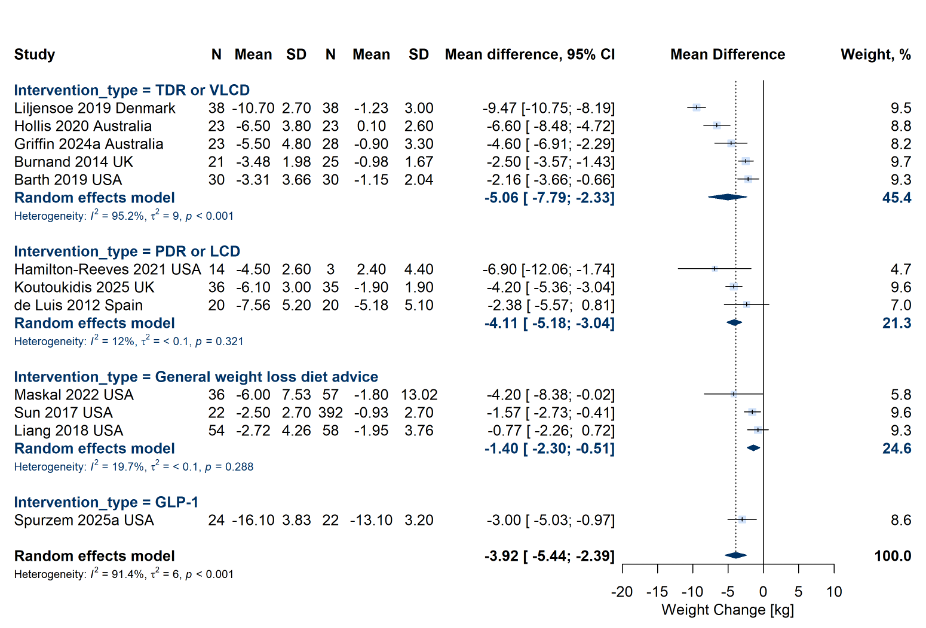Figure S 16. Association between weight loss interventions and weight change |
| --- |

| 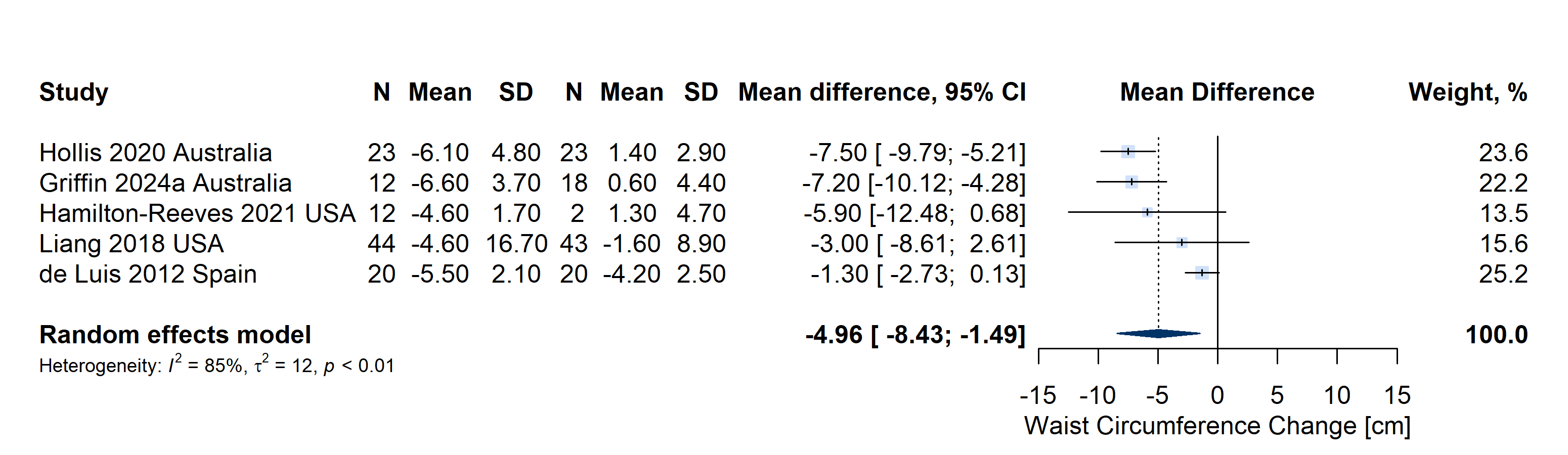 |
| --- |
| Figure S 17. Association between weight loss interventions and waist circumference change |
| 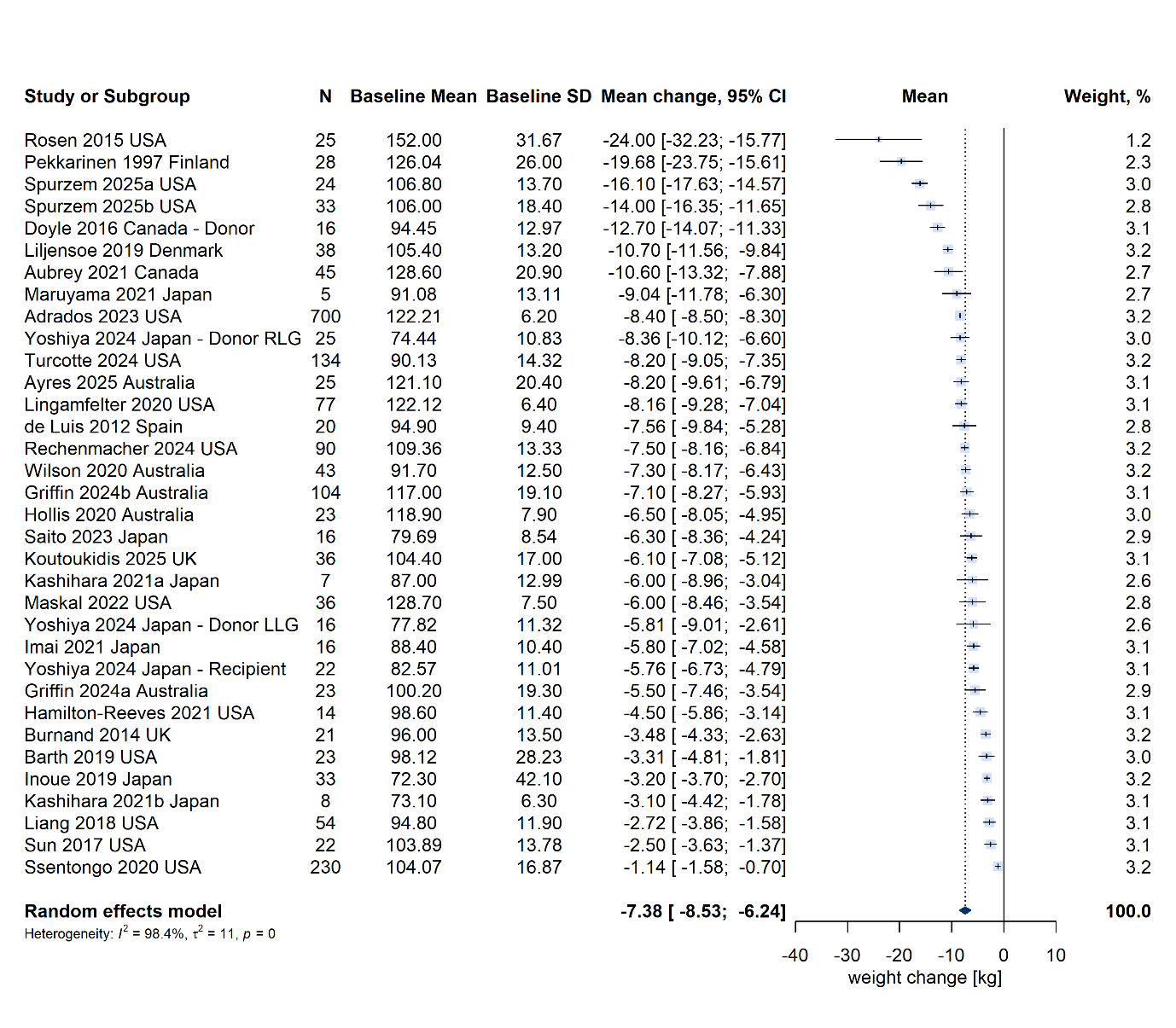 |
| Figure S 18. Association between weight loss interventions and weight change, analysis of single arms |

| 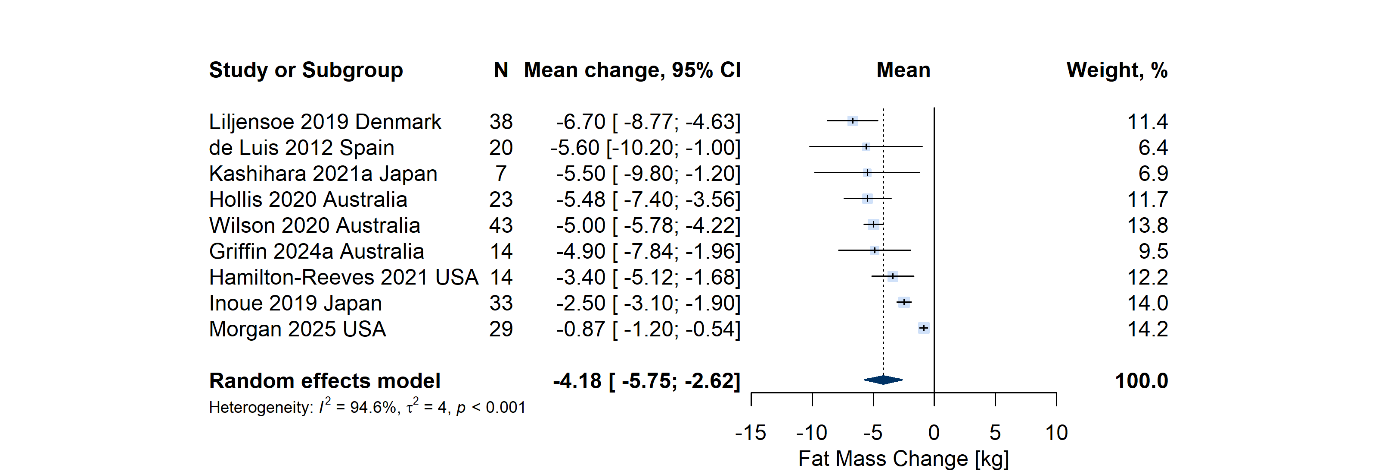 |
| --- |
| Figure S 19. Association between weight loss interventions and fat mass change, analysis of single arms |


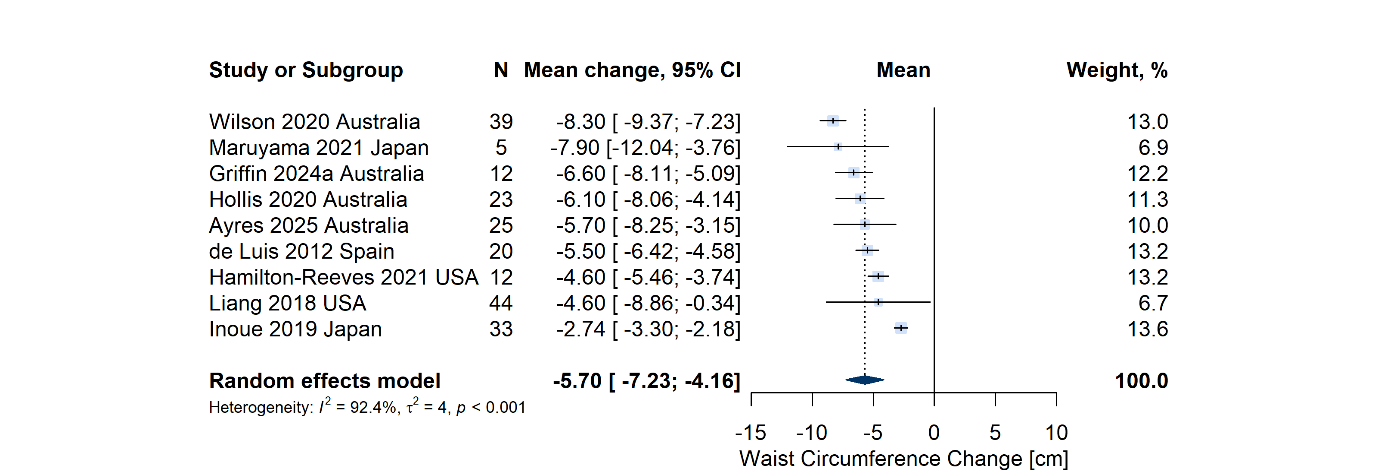


Figure S 20. Association between weight loss interventions and waist circumference change


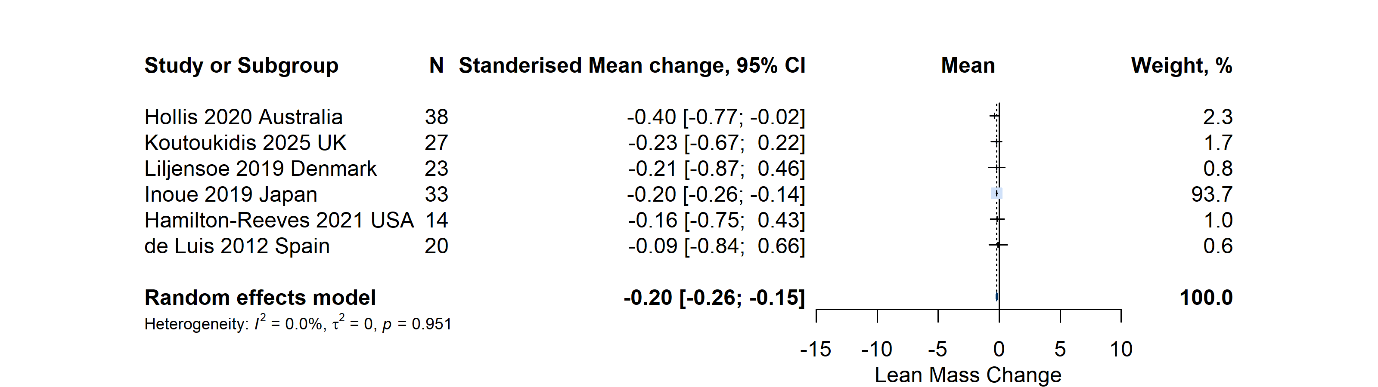


Figure S 21. Association between weight loss interventions and lean mass change, analysis of single arms

| 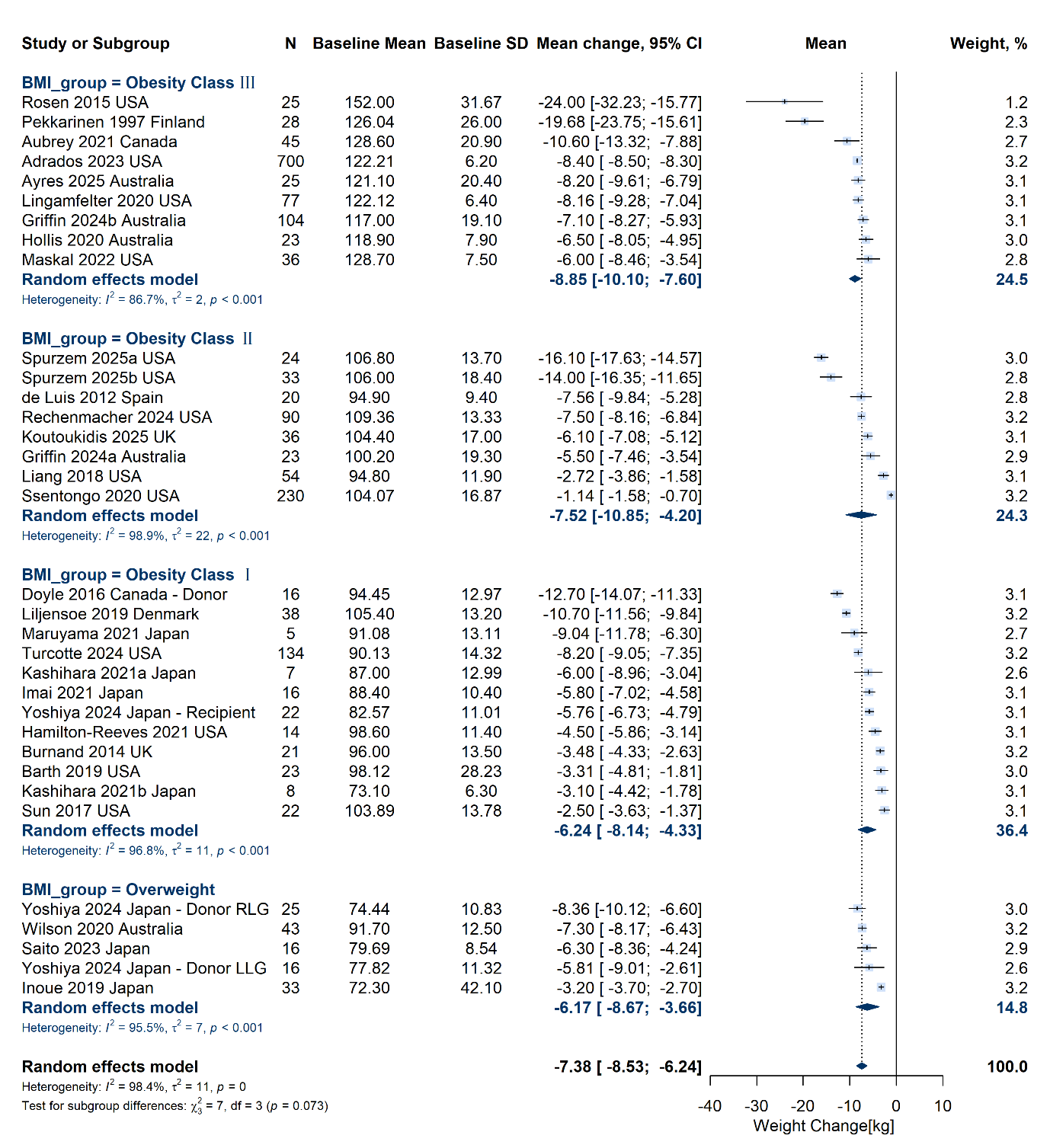 |
| --- |
| Figure S 22. Weight change in each study and by Baseline BMI (Test for subgroup differences: Q = 6.97, df = 3, P = 0.073) |
| 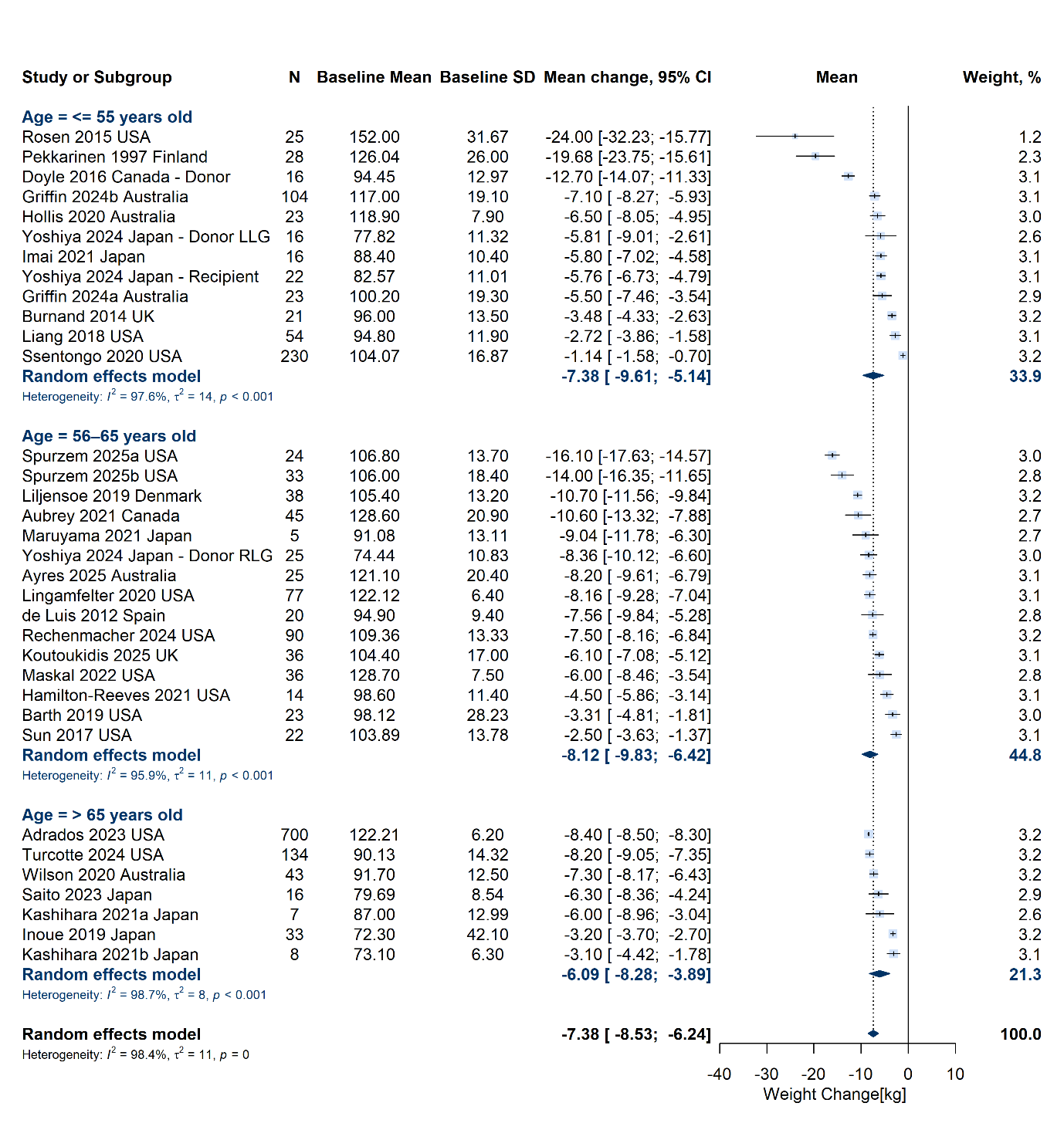 |
| Figure S 23. Weight change in each study by age groups: ≤55, 56–65, and >65 years (Test for subgroup differences: Q = 2.07, df = 2, P = 0.355) |

| 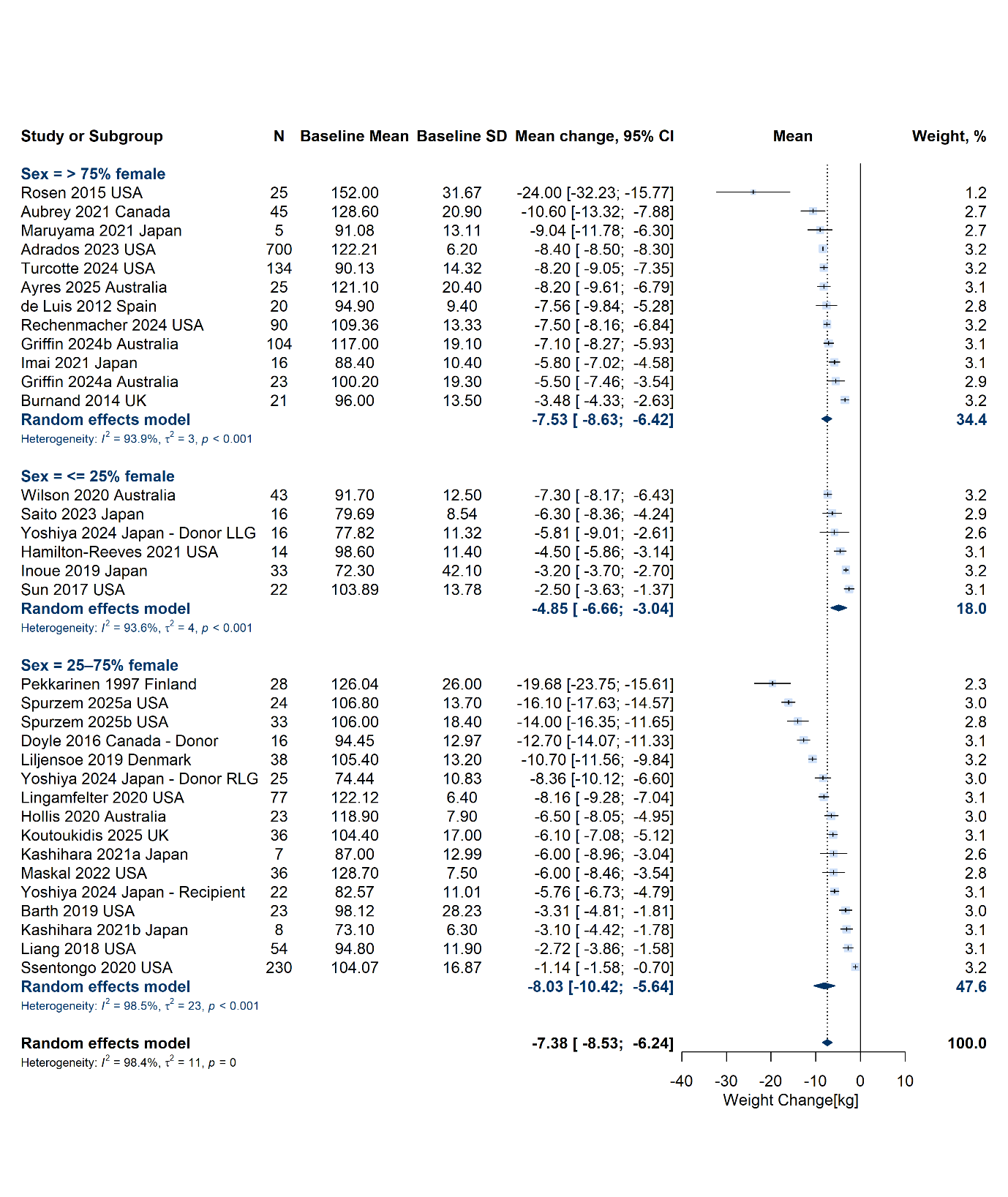 |
| --- |
| Figure S 24. Weight change in each study by sex groups: ＞75% female, 25–75% female, and ≤25% female (Test for subgroup differences: Q = 7.02, df = 2, P = 0.030) |

| 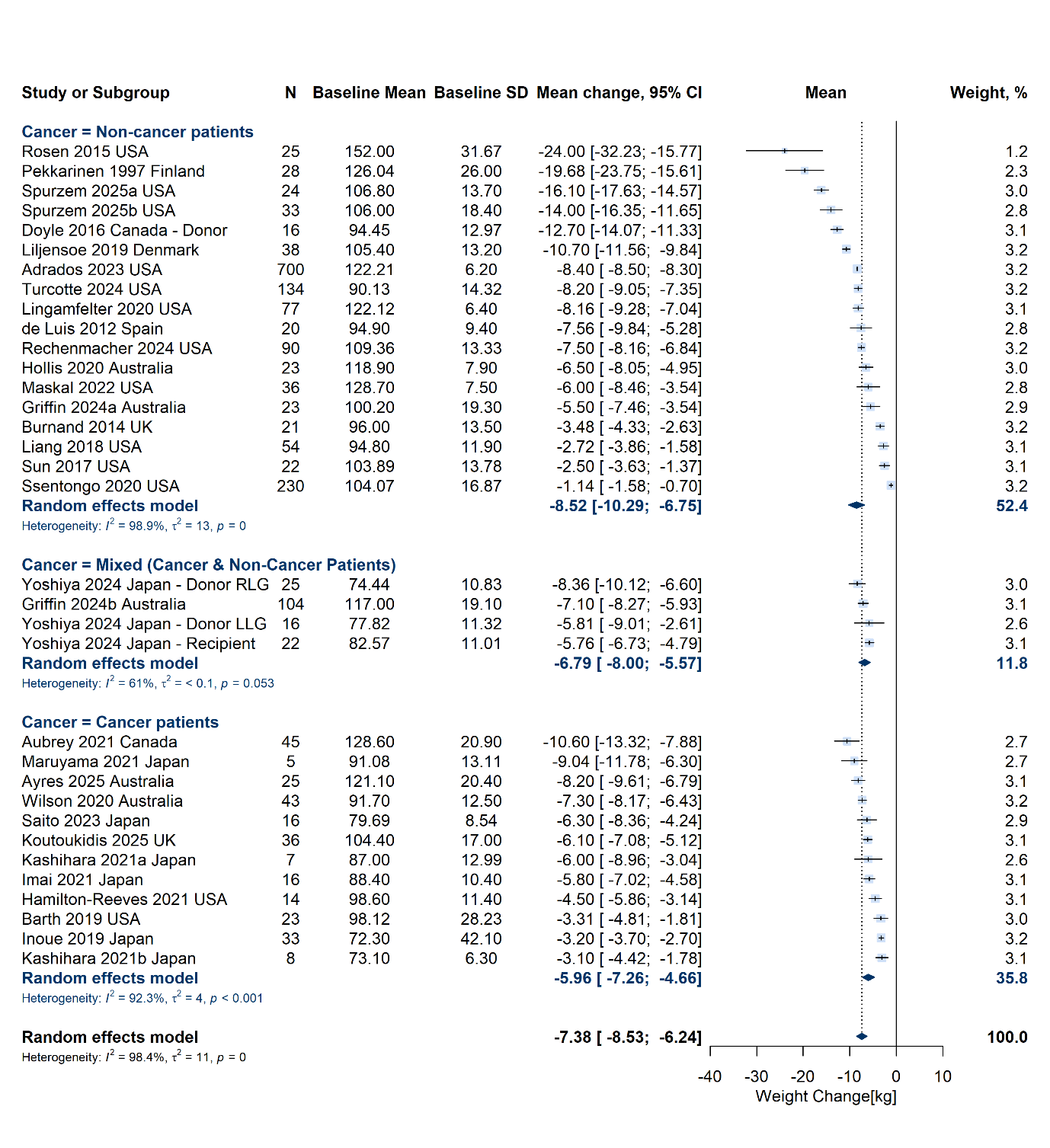 |
| --- |
| Figure S 25. Weight change in each study and by cancer and non-cancer patient groups (Test for subgroup differences: Q = 5.25, df = 2, P = 0.072) |

| Table S 4. Meta-regression estimates for weight change based on intervention duration | | | |
| --- | --- | --- | --- |
| **Predictor** | **Estimate (β)** | **SE** | **95% CI (Lower, Upper)** |
| Intercept | -0.68 | 1.76 | -4.13, 2.77 |
| Length of Intervention | -0.21 *** | 0.06 | -0.33, -0.09 |
| PDR or LCD (vs. Advice only) | -4.18 * | 1.69 | -7.48, -0.87 |
| TDR or VLCD (vs. Advice only) | -6.37 *** | 1.80 | -9.90, -2.85 |
| GLP-1 (vs. Advice only) | -7.76** | 2.67 | -12.98, -2.53 |
| SE = standard error; CI = confidence interval. Statistical significance: ***p < 0.001, **p < 0.01, *p < 0.05. | | | |

##
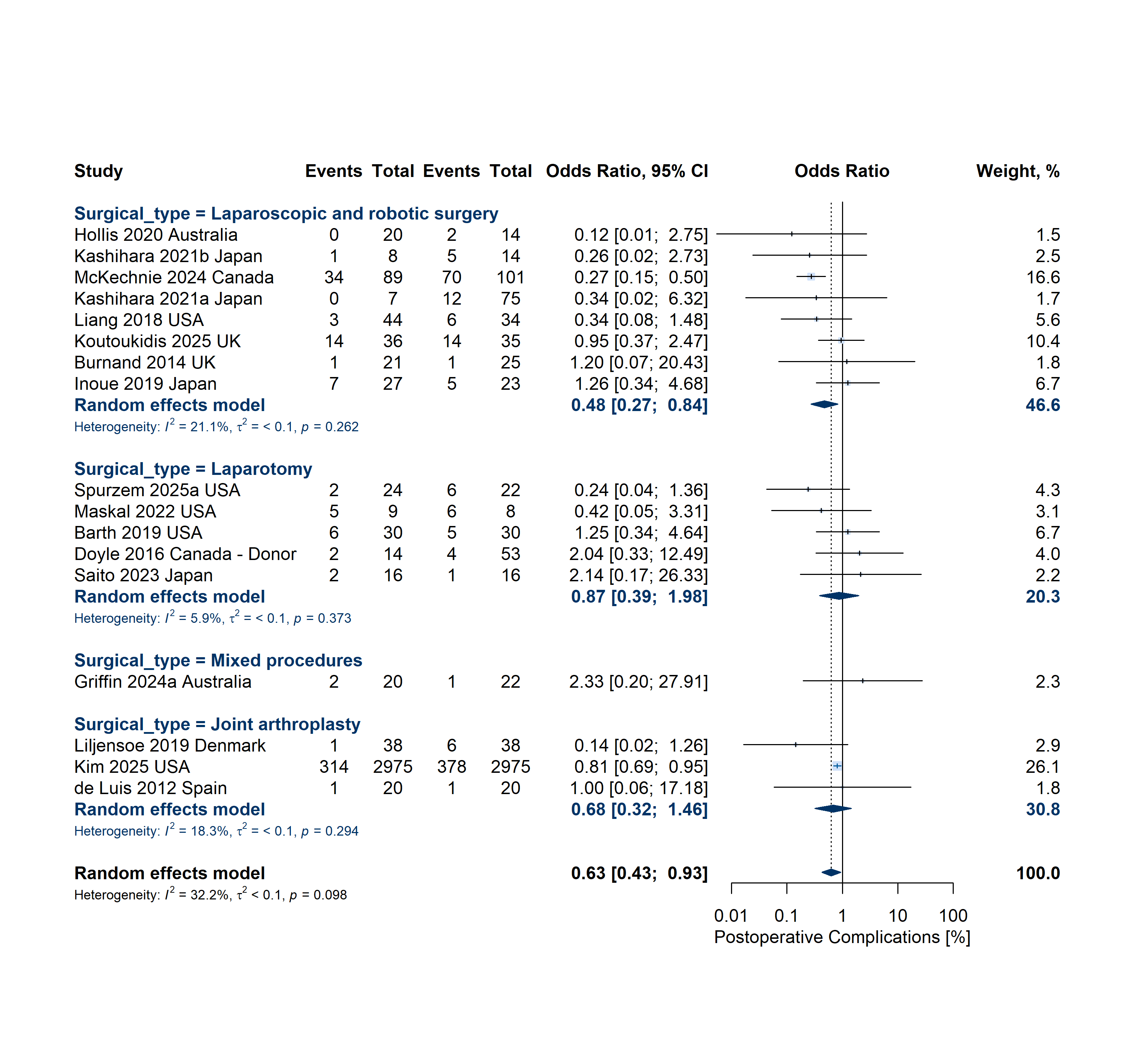


Figure S26. Association between weight loss interventions and any postoperative complication in subgroup analyses

| Table S 5. Risk of Bias assessed using the Cochrane Risk of Bias 2 tool (RCTs) and the ROBINS-I toll for all other trials | | | | | | | | | | | |
| --- | --- | --- | --- | --- | --- | --- | --- | --- | --- | --- | --- |
| **Date** | **Author year** | **D1** | **D2** | **D3** | **D4** | **D5** | **D6** | **D7** | **OVERALL** | **Bias tools** | **Combined Bias Risk Level** |
| 2024 | Griffin et al, 2024a Australia | Low | Low | Low | Low | Low | NA | NA | Low | RoB2 | Low Risk |
| 2024 | Griffin et al, 2024b Australia | Moderate | Low | Low | Low | Moderate | Moderate | Low | Moderate | ROBINS-I | Moderate Risk |
| 2021 | Hamilton-Reeves et al, 2021 USA | Moderate | Low | Moderate | Low | Low | Moderate | Low | Moderate | ROBINS-I | Moderate Risk |
| 2022 | Liang et al, 2018 USA | Low | Some concerns | Low | Low | Low | NA | NA | Some concerns | ROB2 | Moderate Risk |
| 2012 | de Luis et al, 2012 Spain | high | Low | Low | Low | Some concerns | NA | NA | high | ROB2 | High Risk |
| 2023 | Adrados et al, 2023 USA | Low | Moderate | Low | Low | Moderate | Low | Low | Moderate | ROBINS-I | Moderate Risk |
| 2015 | Rosen et al, 2015 USA | Moderate | Serious | Low | Low | Low | Moderate | Low | Serious | ROBINS-I | High Risk |
| 2021 | Kashihara et al, 2021 Japan | Moderate | Low | Low | Low | Low | Serious | Low | Serious | ROBINS-I | High Risk |
| 2017 | Sun et al, 2017 USA | Moderate | Serious | Moderate | Serious | Serious | Serious | Low | Serious | ROBINS-I | High Risk |
| 2016 | Burnand et al, 2014 UK | Some concerns | Low | Low | Low | Low | NA | NA | Some concerns | ROB2 | Moderate Risk |
| 2020 | Wilson et al, 2020 Australia | Low | Low | Low | Moderate | Low | Moderate | Low | Moderate | ROBINS-I | Moderate Risk |
| 2021 | Maruyama et al, 2021 Japan | Moderate | Critical | Moderate | Low | Serious | Moderate | Low | Critical | ROBINS-I | High Risk |
| 2019 | Liljensoe et al, 2019 Denmark | Low | Low | Low | Low | Low | NA | NA | Low | ROB2 | Low Risk |
| 2021 | Aubrey et al, 2021 Canada | Moderate | Serious | Serious | Low | Moderate | Moderate | Low | Serious | ROBINS-I | High Risk |
| 2023 | Saito et al, 2023 Japan | Low | Moderate | Moderate | Low | Low | Low | Low | Moderate | ROBINS-I | Moderate Risk |
| 2024 | Rechenmacher et al, 2024 USA | Moderate | Low | Moderate | Moderate | Moderate | Moderate | Low | Moderate | ROBINS-I | Moderate Risk |
| 2021 | Kashihara et al, 2021 Japan | Moderate | Moderate | Moderate | Moderate | Serious | Moderate | Low | Serious | ROBINS-I | High Risk |
| 2020 | Lingamfelter et al, 2020 USA | Moderate | Serious | Serious | Low | Moderate | Moderate | Low | Serious | ROBINS-I | High Risk |
| 2024 | Turcotte et al, 2024 USA | Moderate | Serious | Serious | Critical | Serious | Moderate | Low | Critical | ROBINS-I | High Risk |
| 2019 | Barth et al, 2019 USA | Low | Low | Low | Low | Low | NA | NA | Low | ROB2 | Low Risk |
| 2020 | Hollis et al, 2020 Australia | Low | Low | Low | Low | Low | NA | NA | Low | ROB2 | Low Risk |
| 2024 | Yoshiya et al, 2024 Japan | Moderate | Low | Low | Moderate | Moderate | Moderate | Low | Moderate | ROBINS-I | Moderate Risk |
| 2020 | Ssentongo et al, 2020 USA | Moderate | Moderate | Moderate | Moderate | Moderate | Low | Low | Moderate | ROBINS-I | Moderate Risk |
| 2022 | Maskal et al, 2022 USA | Moderate | Low | Moderate | Moderate | Moderate | Moderate | Low | Moderate | ROBINS-I | Moderate Risk |
| 2019 | Inoue et al, 2019 Japan | Low | Low | Low | Low | Low | Low | Low | Low | ROBINS-I | Low Risk |
| 2021 | Imai et al, 2021 Japan | Moderate | Low | Low | Moderate | Moderate | Moderate | Low | Moderate | ROBINS-I | Moderate Risk |
| 2016 | Doyle et al, 2016 Canada | Moderate | Low | Low | Low | Low | Moderate | Low | Moderate | ROBINS-I | Moderate Risk |
| 1997 | Pekkarinen et al, 1997 Finland | Moderate | Low | Low | Low | Moderate | Low | Low | Moderate | ROBINS-I | Moderate Risk |
| 2024 | McKechnie et al, 2024 Canada | Moderate | Moderate | Low | Serious | Low | Low | Low | Serious | ROBINS-I | High Risk |
| 2025 | Koutoukidis et al, 2025 UK | Low | Low | Low | Low | Low | NA | NA | Low | ROB2 | Low Risk |
| 2025 | Morgan et al, 2025 USA | Low | Serious | Moderate | Low | Moderate | Moderate | Low | Serious | ROBINS-I | High Risk |
| 2025 | Spurzem et al 2025a USA | Low | Low | Low | Low | Low | Low | Low | Low | ROBINS-I | High Risk |
| 2025 | Kim et al, 2025 USA | Low | Low | Moderate | Low | Low | Moderate | Low | Moderate | ROBINS-I | High Risk |
| 2025 | Spurzem et al, 2025b USA | Moderate | Moderate | Moderate | Moderate | Low | Low | Low | Moderate | ROBINS-I | High Risk |
| 2025 | Ayres et al, 2025 Australia | Moderate | Low | Low | Low | Low | Low | Low | Moderate | ROBINS-I | High Risk |
| To ensure consistency in risk of bias assessment across randomized and non-randomized studies, the RoB 2 and ROBINS-I categories were harmonized into three levels: low risk, moderate risk, and high risk. Studies rated as "Low risk" by both tools were classified as "Low risk". "Some concerns" in RoB 2 and "Moderate risk" in ROBINS-I were grouped as "Moderate risk". "High risk" in RoB 2 and "Serious" or "Critical risk" in ROBINS-I were classified as "High risk". | | | | | | | | | | | |

| 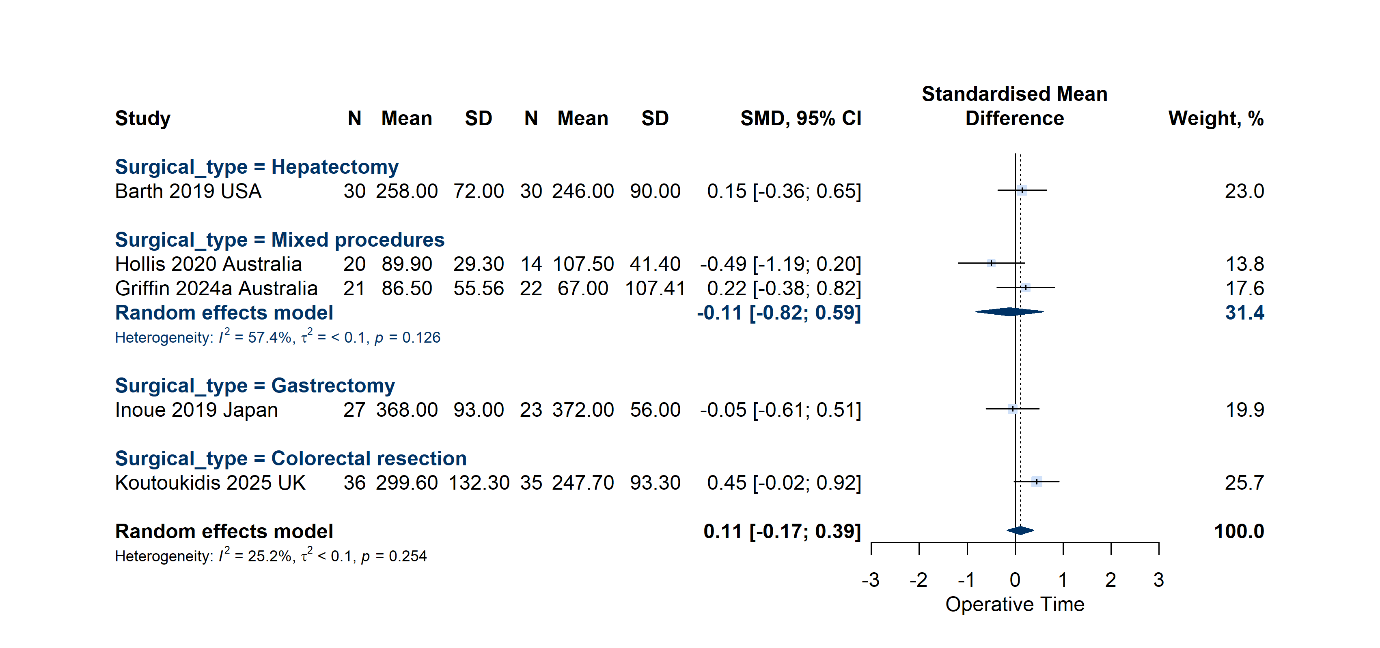 |
| --- |
| Figure S 27. Association between weight loss intervention and operative time among the studies with low risk of bias (sensitivity analysis) |

| 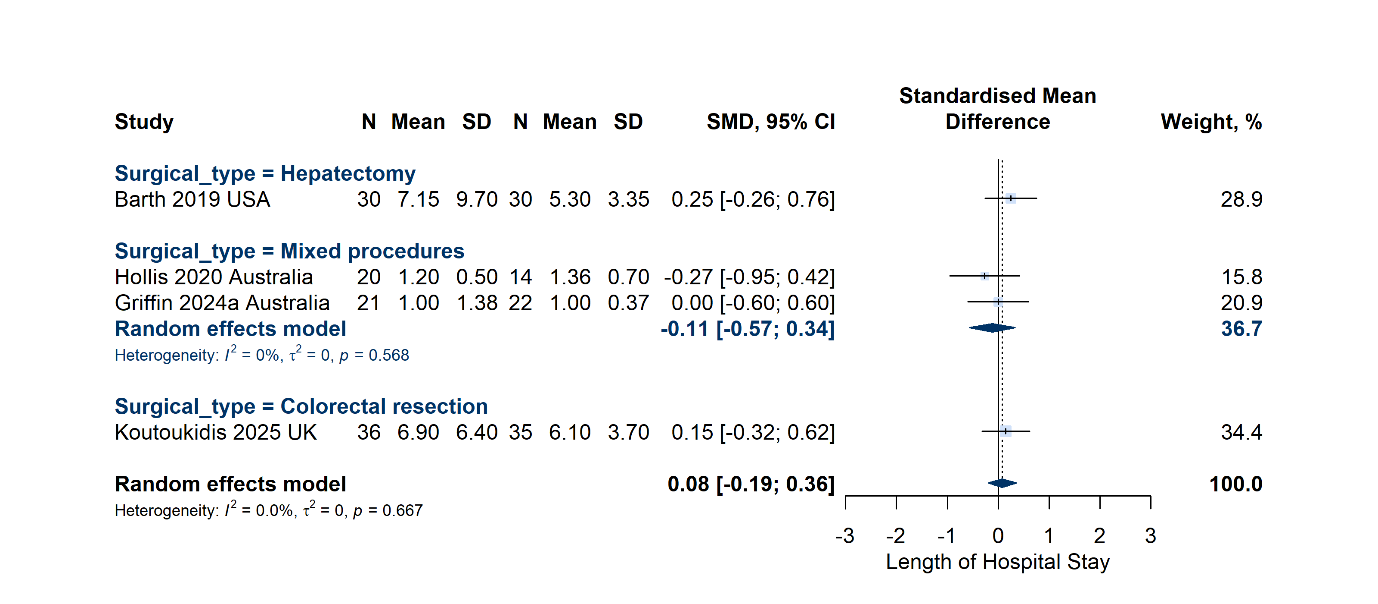 |
| --- |
| Figure S 28. Association between weight loss interventions and length of hospital stay among the studies with low risk of bias (sensitivity analysis) |

| 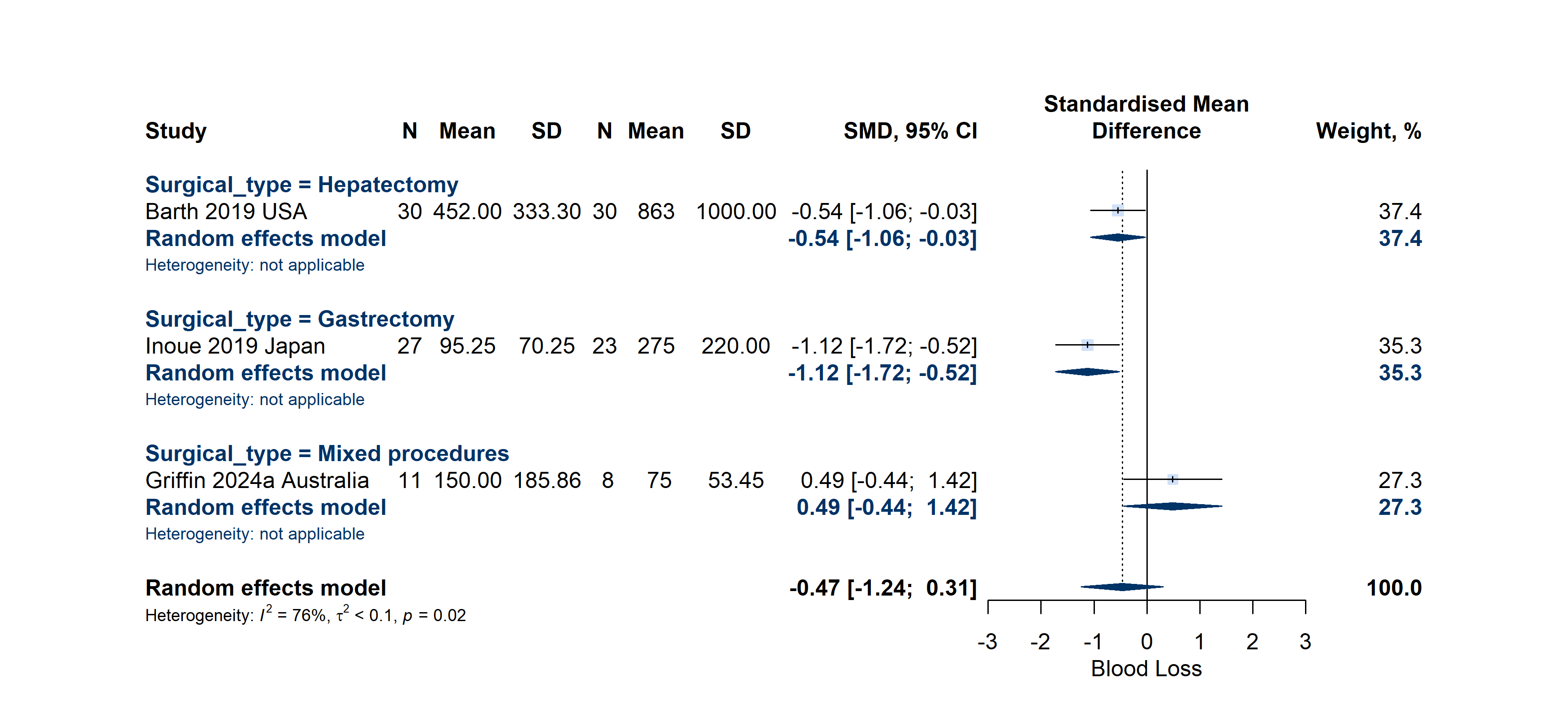 |
| --- |
| Figure S 29. Association between weight loss interventions and blood loss among the studies with low risk of bias (sensitivity analysis) |
| 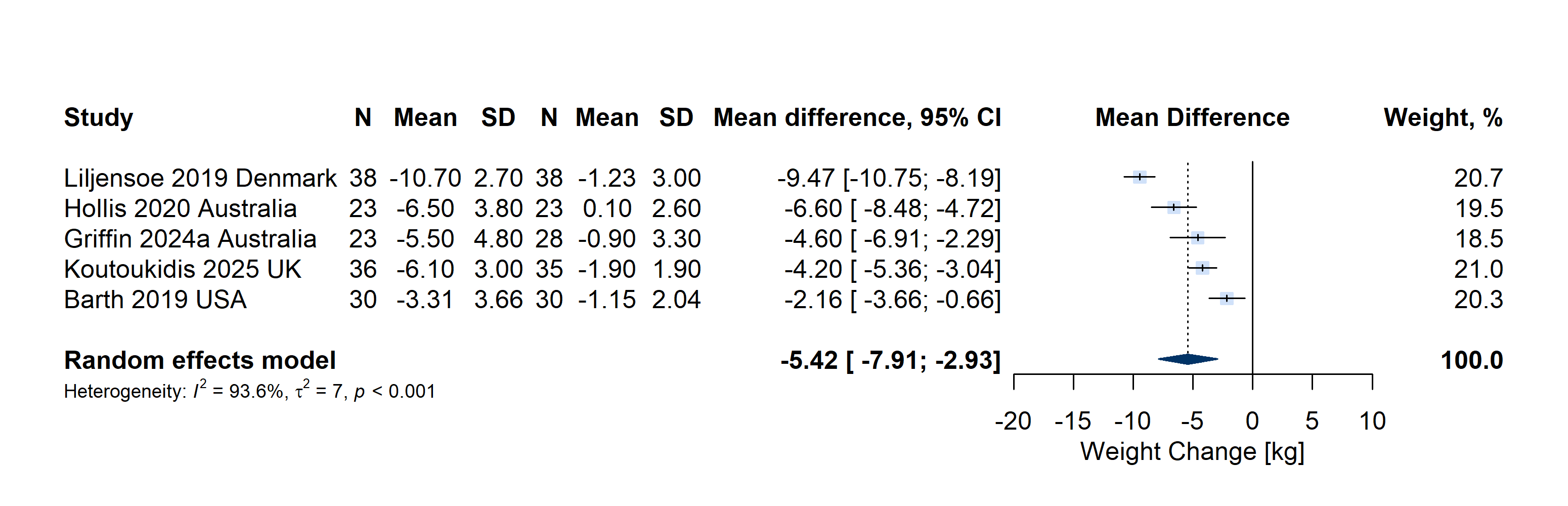 |
| Figure S 30. Association between weight loss interventions and weight change among the studies with low risk of bias (sensitivity analysis) |


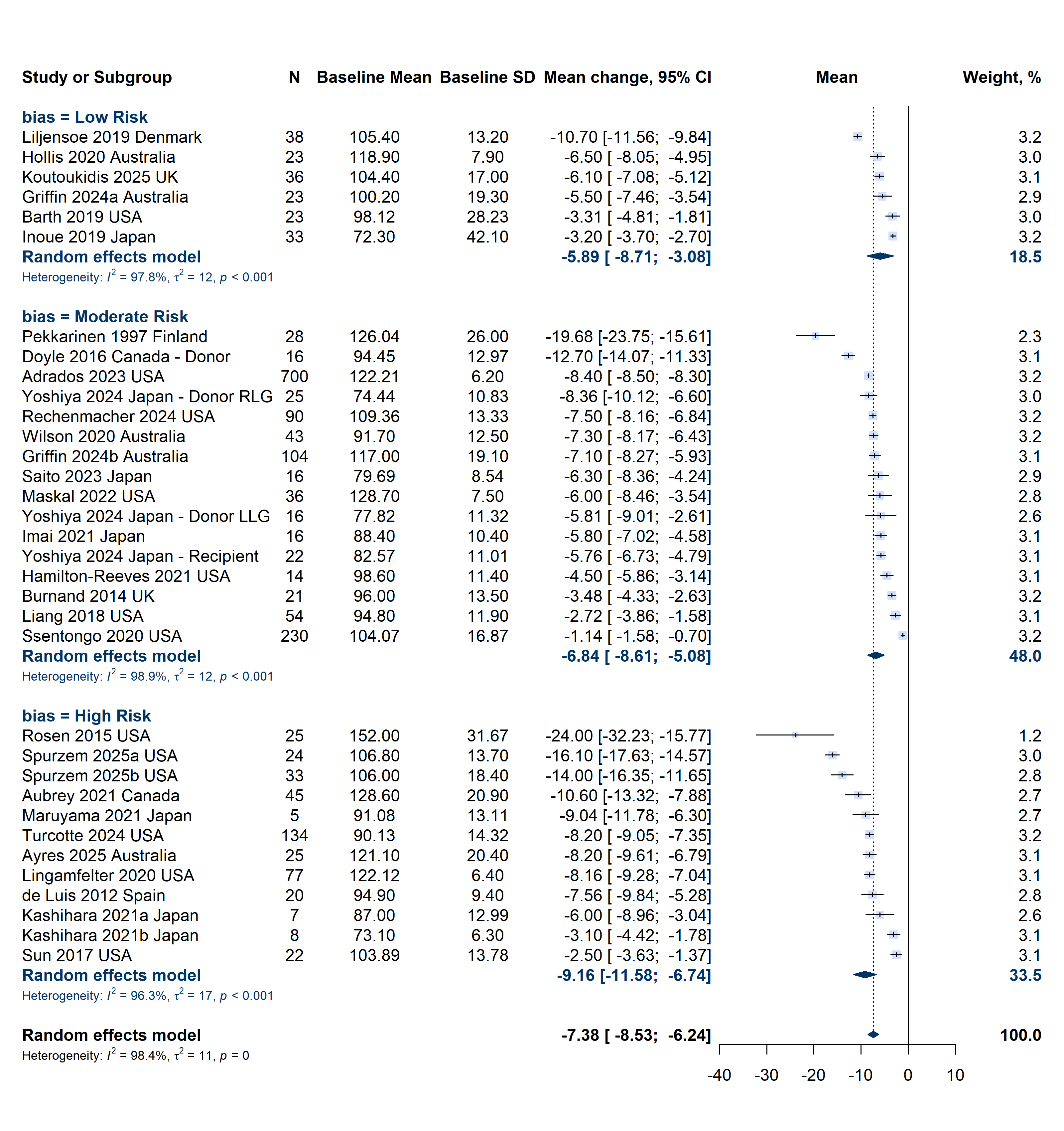


Figure S 31. Subgroup analysis of weight change stratified by risk of bias (sensitivity analysis)


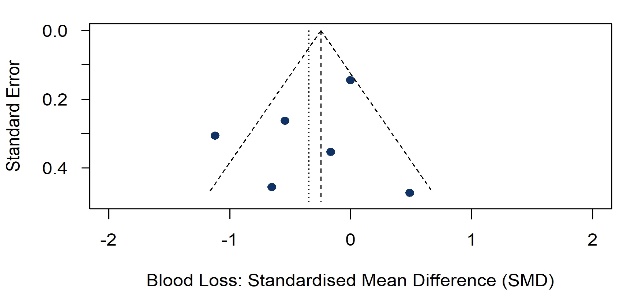

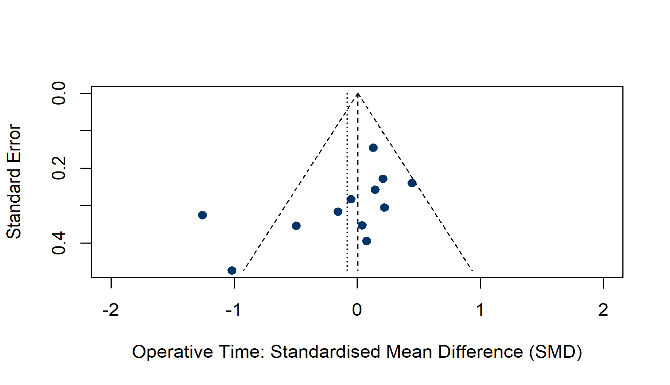


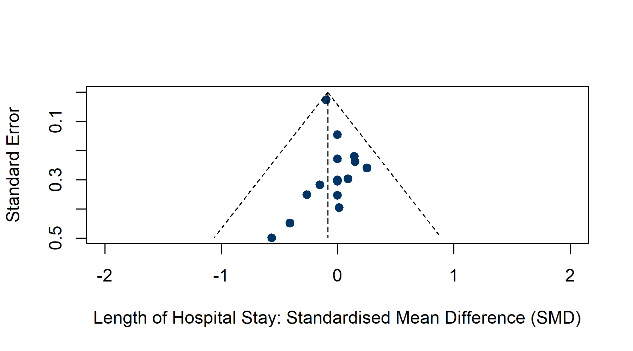

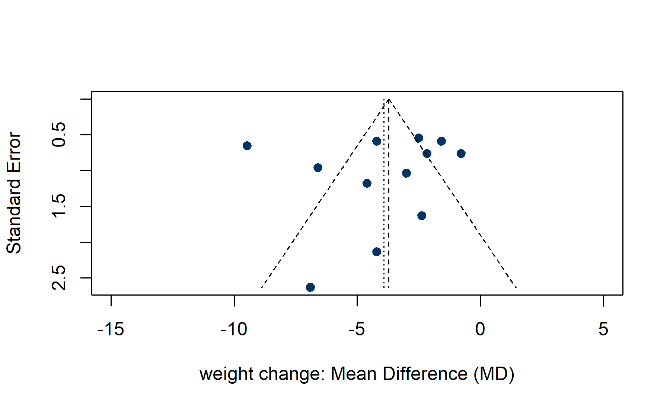


Figure S 32. Funnel plots for publication bias in comparative trials

Each plot shows the relationship between the standard error and effect size for the outcomes of blood loss, operative time, length of hospital stay, and weight change. Dashed lines represent 95% confidence limits. No significant publication bias was detected based on Egger’s test for blood loss (z = 0.08, *P* = 0.94), length of hospital stay (z = 0.09, *P* = 0.36), or weight change (z = –0.57, *P* = 0.57), whereas a significant small-study effect was observed for operative time (z = –2.12, P = 0.03)

Table 6 PRISMA Checklist

| **Section and Topic** | **Item #** | **Checklist item** | **Location where item is reported** |
| --- | --- | --- | --- |
| **TITLE** | | |  |
| Title | 1 | Identify the report as a systematic review. | Title |
| **ABSTRACT** | | |  |
| Abstract | 2 | See the PRISMA 2020 for Abstracts checklist. | Abstract |
| **INTRODUCTION** | | |  |
| Rationale | 3 | Describe the rationale for the review in the context of existing knowledge. | Introduction, page 4 |
| Objectives | 4 | Provide an explicit statement of the objective(s) or question(s) the review addresses. | Introduction, page 4 |
| **METHODS** | | |  |
| Eligibility criteria | 5 | Specify the inclusion and exclusion criteria for the review and how studies were grouped for the syntheses. | Methods, Eligibility criteria, page 5 |
| Information sources | 6 | Specify all databases, registers, websites, organisations, reference lists and other sources searched or consulted to identify studies. Specify the date when each source was last searched or consulted. | Methods, Process, page 5 |
| Search strategy | 7 | Present the full search strategies for all databases, registers and websites, including any filters and limits used. | Supplementary documents, Method S1 Search strategy |
| Selection process | 8 | Specify the methods used to decide whether a study met the inclusion criteria of the review, including how many reviewers screened each record and each report retrieved, whether they worked independently, and if applicable, details of automation tools used in the process. | Methods, Process, page 5 |
| Data collection process | 9 | Specify the methods used to collect data from reports, including how many reviewers collected data from each report, whether they worked independently, any processes for obtaining or confirming data from study investigators, and if applicable, details of automation tools used in the process. | Methods, Process, page 5-6 |
| Data items | 10a | List and define all outcomes for which data were sought. Specify whether all results that were compatible with each outcome domain in each study were sought (e.g. for all measures, time points, analyses), and if not, the methods used to decide which results to collect. | Methods, Data analysis, page 5-6 |
|  | 10b | List and define all other variables for which data were sought (e.g. participant and intervention characteristics, funding sources). Describe any assumptions made about any missing or unclear information. | Methods, Data analysis, page 6 |
| Study risk of bias assessment | 11 | Specify the methods used to assess risk of bias in the included studies, including details of the tool(s) used, how many reviewers assessed each study and whether they worked independently, and if applicable, details of automation tools used in the process. | Methods, Risk of bias (quality) assessment, page 6 |
| Effect measures | 12 | Specify for each outcome the effect measure(s) (e.g. risk ratio, mean difference) used in the synthesis or presentation of results. | Methods, Data analysis, page 6-7 |
| Synthesis methods | 13a | Describe the processes used to decide which studies were eligible for each synthesis (e.g. tabulating the study intervention characteristics and comparing against the planned groups for each synthesis (item #5)). | Methods, Data analysis, page 6-7 |
|  | 13b | Describe any methods required to prepare the data for presentation or synthesis, such as handling of missing summary statistics, or data conversions. | Methods, Data analysis, page 6-7 |
|  | 13c | Describe any methods used to tabulate or visually display results of individual studies and syntheses. | Methods, Data analysis, page 6-7 |
|  | 13d | Describe any methods used to synthesize results and provide a rationale for the choice(s). If meta-analysis was performed, describe the model(s), method(s) to identify the presence and extent of statistical heterogeneity, and software package(s) used. | Methods, Data analysis, page 6 |
|  | 13e | Describe any methods used to explore possible causes of heterogeneity among study results (e.g. subgroup analysis, meta-regression). | Methods, Data analysis, page 6 |
|  | 13f | Describe any sensitivity analyses conducted to assess robustness of the synthesized results. | Methods, Data analysis, page 6 |
| Reporting bias assessment | 14 | Describe any methods used to assess risk of bias due to missing results in a synthesis (arising from reporting biases). | Methods, Risk of bias (quality) assessment, page 6 |
| Certainty assessment | 15 | Describe any methods used to assess certainty (or confidence) in the body of evidence for an outcome. | Methods, Risk of bias, page 6 |
| **RESULTS** | | |  |
| Study selection | 16a | Describe the results of the search and selection process, from the number of records identified in the search to the number of studies included in the review, ideally using a flow diagram. | Figure S1 in the Supplement |
|  | 16b | Cite studies that might appear to meet the inclusion criteria, but which were excluded, and explain why they were excluded. | Study selection and characteristics, page 8 |
| Study characteristics | 17 | Cite each included study and present its characteristics. | Study selection and characteristics, page 8 |
| Risk of bias in studies | 18 | Present assessments of risk of bias for each included study. | Table S5 in the Supplement |
| Results of individual studies | 19 | For all outcomes, present, for each study: (a) summary statistics for each group (where appropriate) and (b) an effect estimate and its precision (e.g. confidence/credible interval), ideally using structured tables or plots. | Figure 1 to 6, page 22-23 |
| Results of syntheses | 20a | For each synthesis, briefly summarise the characteristics and risk of bias among contributing studies. | Risk of bias and sensitivity analyses, page 11 |
|  | 20b | Present results of all statistical syntheses conducted. If meta-analysis was done, present for each the summary estimate and its precision (e.g. confidence/credible interval) and measures of statistical heterogeneity. If comparing groups, describe the direction of the effect. | Results, page9-10 |
|  | 20c | Present results of all investigations of possible causes of heterogeneity among study results. | Results, page 10 |
|  | 20d | Present results of all sensitivity analyses conducted to assess the robustness of the synthesized results. | Results, page 10 -11 |
| Reporting biases | 21 | Present assessments of risk of bias due to missing results (arising from reporting biases) for each synthesis assessed. | Risk of bias and sensitivity analyses, page 11 |
| Certainty of evidence | 22 | Present assessments of certainty (or confidence) in the body of evidence for each outcome assessed. | Result, page 11 |
| **DISCUSSION** | | |  |
| Discussion | 23a | Provide a general interpretation of the results in the context of other evidence. | Discussion, page 12-13 |
|  | 23b | Discuss any limitations of the evidence included in the review. | Discussion, page 13 |
|  | 23c | Discuss any limitations of the review processes used. | Discussion, page 13-14 |
|  | 23d | Discuss implications of the results for practice, policy, and future research. | Discussion, page 14 |
| **OTHER INFORMATION** | | |  |
| Registration and protocol | 24a | Provide registration information for the review, including register name and registration number, or state that the review was not registered. | Methods, page 4. The Impact of Preoperative Weight Loss Interventions on Outcomes after Elective Non-bariatric Surgery, ID: CRD42024610636 |
|  | 24b | Indicate where the review protocol can be accessed, or state that a protocol was not prepared. | Methods, page 4 |
|  | 24c | Describe and explain any amendments to information provided at registration or in the protocol. | Methods, page 4 |
| Support | 25 | Describe sources of financial or non-financial support for the review, and the role of the funders or sponsors in the review. | Acknowledgements, page 15 |
| Competing interests | 26 | Declare any competing interests of review authors. | Conflicts of interest, page 15 |
| Availability of data, code and other materials | 27 | Report which of the following are publicly available and where they can be found: template data collection forms; data extracted from included studies; data used for all analyses; analytic code; any other materials used in the review. | Data availability, page 15 |
